# Eukaryotic tRNA sequences present conserved and amino acid-specific structural signatures

**DOI:** 10.1093/nar/gkac222

**Published:** 2022-04-05

**Authors:** Eric Westhof, Bryan Thornlow, Patricia P Chan, Todd M Lowe

**Affiliations:** Université de Strasbourg, Institut de Biologie Moléculaire et Cellulaire, Architecture et Réactivité de l’ARN, CNRS UPR 9002, 2, allée Konrad Roentgen, F-67084 Strasbourg, France; Department of Biomolecular Engineering, Baskin School of Engineering, University of California Santa Cruz, Santa Cruz, CA 95064, USA; UCSC Genomics Institute, University of California Santa Cruz, Santa Cruz, CA 95064, USA; Department of Biomolecular Engineering, Baskin School of Engineering, University of California Santa Cruz, Santa Cruz, CA 95064, USA; UCSC Genomics Institute, University of California Santa Cruz, Santa Cruz, CA 95064, USA; Department of Biomolecular Engineering, Baskin School of Engineering, University of California Santa Cruz, Santa Cruz, CA 95064, USA; UCSC Genomics Institute, University of California Santa Cruz, Santa Cruz, CA 95064, USA

## Abstract

Metazoan organisms have many tRNA genes responsible for decoding amino acids. The set of all tRNA genes can be grouped in sets of common amino acids and isoacceptor tRNAs that are aminoacylated by corresponding aminoacyl-tRNA synthetases. Analysis of tRNA alignments shows that, despite the high number of tRNA genes, specific tRNA sequence motifs are highly conserved across multicellular eukaryotes. The conservation often extends throughout the isoacceptors and isodecoders with, in some cases, two sets of conserved isodecoders. This study is focused on non-Watson–Crick base pairs in the helical stems, especially GoU pairs. Each of the four helical stems may contain one or more conserved GoU pairs. Some are amino acid specific and could represent identity elements for the cognate aminoacyl tRNA synthetases. Other GoU pairs are found in more than a single amino acid and could be critical for native folding of the tRNAs. Interestingly, some GoU pairs are anticodon-specific, and others are found in phylogenetically-specific clades. Although the distribution of conservation likely reflects a balance between accommodating isotype-specific functions as well as those shared by all tRNAs essential for ribosomal translation, such conservations may indicate the existence of specialized tRNAs for specific translation targets, cellular conditions, or alternative functions.

## INTRODUCTION

The set of all tRNA genes can be grouped into sets of isoacceptor tRNAs that are aminoacylated by the corresponding aminoacyl-tRNA synthetase, one per amino acid. The number of tRNA genes for the sets of tRNA isoacceptors can vary widely (1). Within each set of tRNA isoacceptors, the number of tRNA genes with the same anticodon triplet, the isodecoders (2,3), also varies (2,3). The central role of tRNAs in protein translation necessitates interactions with several other entities within the cell (4). tRNA transcription requires sequence-specific binding of transcription factors to their A- and B-box regions (5–10), and tRNA maturation requires interactions with RNases P and Z (11–13) plus a host of RNA modification enzymes (14). Fundamentally, native and mature tRNAs interact with the ribosome, mRNA codon, and corresponding aminoacyl tRNA synthetase during translation (15,16). Overall a multitude of factors act to shape or restrict tRNA sequences: the folding process, the 3D architecture (17–20), the interactions with enzymes involved in tRNA maturation (21–25), modification (26–29) and degradation (30–33), aminoacyl tRNA synthetases (34,35); initiation (36) and elongation factors (37), and ribosomal translation recognition sites (38–40), besides the non-canonical functions of tRNAs (41). To ensure that these interactions are not disrupted, tRNA gene sequences and structures are exceptionally well-conserved, even in the face of elevated mutation rates (42). However, in addition to pan-tRNA conservation, we observe isotype- and clade-specific motifs that are also strongly conserved. While these motifs likely play important structural or regulatory roles, the reasons for their isotype-specificity and conservation are unknown and are ripe for exploration.

Non-Watson–Crick base pairs frequently occur in RNA helical stems, especially GoU pairs (guanine paired with uridine). GoU pairs are structurally and functionally of singular interest (43). They display distinguishable molecular recognition features, especially the movement of the U in the major or minor groove (44). This movement leads to a change in helical twist between the framing base pairs from the normal helical angles (45). That helical twist variation can propagate away to the least constrained end of the helical region that contains the GoU pair (for overviews, see (45,46)). Thus, a GoU pair does not need to directly contact the protein or RNA ligand to exert an action on binding efficiency. In tRNAs, GoU pairs are important for well-established tertiary contacts that maintain tRNA fold and function, throughout their interactions with the aminoacyl-tRNA synthetases and the ribosomal machinery. We therefore seek to extend the analysis of tRNA sequences beyond the recently published study on tRNA-Ala and tRNA-Gly in eukaryotes (47).

Here, we attempt to identify base pairing signatures specific to each tRNA isotype that are conserved across several major clades of multicellular eukaryotes, and to relate these observations to known tRNA structures and interactions. To identify specific targets for experimental study in genetically pliable model metazoans, we leverage the broader distribution of tRNA genes currently known across hundreds of related species to ask: (i) are GoU pairs biased for specific stems and positions, and if so, for which amino acids or isodecoders? (ii) when a GoU pair is present, is the orientation, GoU versus UoG, also conserved? We have extracted and structurally aligned *Homo sapiens*, *Mus musculus* and *Bombyx mori* tRNA genes from the Genomic tRNA Database (48). We chose these three genomes as they represent well-studied model organisms from three distinct eukaryotic clades, namely primates, rodents, and insects, and therefore enable us to explore tRNA genomics across these clades in a simple and efficient manner. We generalized to other genomes within *Mammalia* and *Insecta* (especially *Drosophila* species) by tRNAviz (49). It is known that tRNA modifications are central to tRNA functions and that many uridines are replaced by pseudouridines (14,50,51). However, such a modification does not prevent the formation of a wobble pair (40–41) and, since these potential modifications are unknown in a large number of instances, they will not be discussed here.

## MATERIALS AND METHODS

The analyses presented here are based on the genomic database of transfer RNA genes, GtRNAdb 2.0 (48). The database contains alignments of tRNA genes based on the tRNAscan-SE prediction algorithm (52). This is the most used tRNA gene identifier, using covariance models to classify potential tRNA genes, assigning a bit score to each. The bit score can be understood as a measure of how much each tRNA resembles a prototypical tRNA, with higher scoring tRNAs more likely to be transcribed and functional in translation, and lower scoring tRNAs more likely to be non-functional or pseudogenes. The covariance model score can be broken down into components representing the primary sequence conservation and secondary structure conservation (52). Overall scores below 55.0 bits may indicate the presence of a pseudogene, increasing in likelihood of a non-functional gene as the score decreases. Indeed, low-scoring tRNA genes often display non-complementary Watson–Crick pairs in the stem regions, or lack highly conserved residues involved in the architectural fold of the tertiary structure. The sequences are organized according to this overall bit score. For our analyses in this study, we focus on tRNA genes with bit scores of at least 55.

There are generally several isodecoders for each isoacceptor tRNA, but the number varies among species and isotypes (2). For most genomes, a fraction of the predicted isodecoder tRNA gene transcripts have been experimentally observed, and the tRNA modifications are known for still a smaller fraction of those based on the MODOMICS database (8). We extracted the tRNA alignments from the GtRNAdb 2.0 and ensured known tertiary structures were aligned for three species: *Homo sapiens*, *Mus musculus*, and *Bombyx mori*. The tRNA structural alignments for *H. sapiens*, *M. musculus* and *B. mori* are given in Supplementary Supplementary Figure S2 together with the consensus cloverleaf structures of tRNAs of the *Mammalia* and *Insecta* (Supplementary_Data_1 and _2). These observations were supported by analyses of additional genomes of *Insecta* and *Mammalia* using tRNAviz (49). The observations using tRNAviz are provided in the supplementary material, organized and annotated by the types of residues, amino acids and anticodon triplets derived from tRNAs in *Insecta*, *Mammalia*, or both (Supplementary_Data_3). Here, the pairing positions are indicated by ‘:’ (*e.g*. 1:72), Watson–Crick pairs by ‘ = ’ for G = C and ‘-’ for A-U, and non-Watson–Crick pairs by ‘o’ (e.g. GoU or AoG).

For all analyses regarding gene counts in primate species, we used a whole-genome alignment containing 7 primate species (human, chimpanzee, gorilla, orangutan, rhesus macaque, grey mouse lemur, Nancy Ma's night monkey), among other species, from our previous work (53). We used tRNAscan-SE 2.0 on these seven genomes to count the number of high-confidence tRNA genes with each anticodon in each species, excluding those in segmental duplications. We then counted the number of unique sequences across these gene sets, and calculated the mean and standard deviations across these genomes for depiction in Figure 2.

For the analyses in Figure 4, we first aligned all high-confidence tRNA genes from the hg19 human reference genome and generated an alignment using cmalign. We then assigned each nucleotide a Sprinzl position based on these alignments (54). We then downloaded data from dbSNP release 153 (55–57) using the UCSC Table Browser (58). For each position in the genome corresponding to a GoU or UoG base pair in a tRNA in the reference genome, we compared the allele frequency of the most common SNP disrupting this base pair, to the allele frequency of the most common SNP disrupting a non-GoU or UoG base pair at an equivalent position in a different tRNA. We found for 20 of 24 comparisons that the minor allele frequency for the allele disrupting the GoU or UoG base pair was lower, and used a sign test to find *P* < 6.3 × 10^–4^. Similarly, we also collected phyloP data (59) for all positions within tRNAs across seven primate species using a Cactus graph from a previous study (53,60), and compared the minimum phyloP score across the positions contributing to a GoU or UoG base pair in a tRNA to the minimum phyloP score across the equivalent positions in tRNAs without GoU or UoG base pairs. We found that for 14 of 23 comparisons, the GoU base pairs had higher phyloP scores than equivalent positions in other tRNA genes, but this was not statistically significant based on the sign test (*P* = 0.202).

## RESULTS

**Figure 1.**
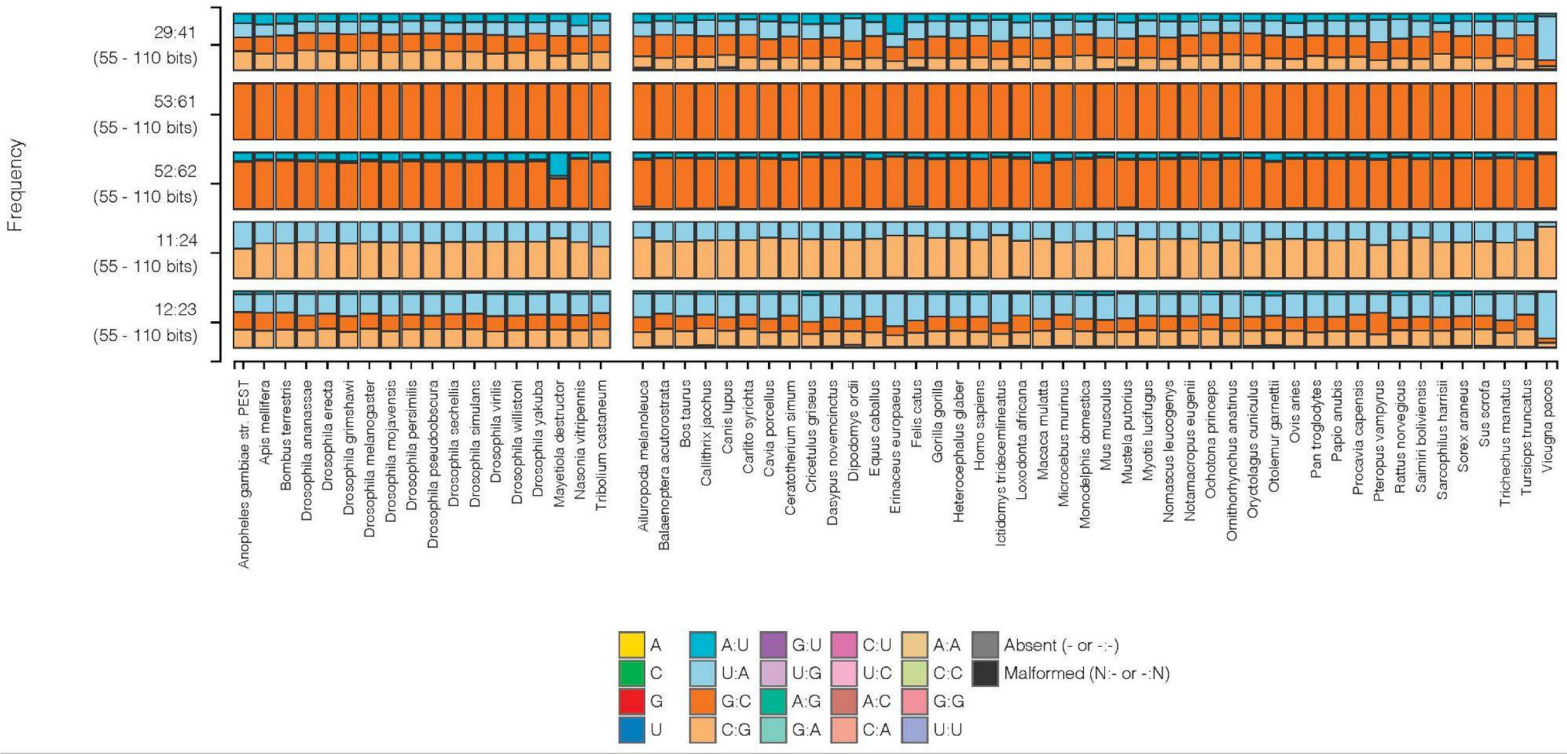
Examples of different levels of Watson–Crick base pair diversity in *Insecta* and *Mammalia*. Each column represents the distribution of nucleotides at the indicated stem position across all tRNAs for the species listed at the bottom (insects on left, mammals on right). This figure was generated using the Compare By Species tool in tRNAviz (49). Only tRNA genes with scores higher than 55 are considered. At the top, the 29:41 base pair in the AC-stem displays all four standard Watson–Crick pairs. Below, the last base pair of the T-stem is invariant and always G53 = C61. The 52:62 T-stem base pair prefers a purine:pyrimidine pair RoY. The pair 11:24 in the D-stem is either U-A or C = G. Finally, the base pair 12:23 in the D-stem is distributed among the four basic Watson–Crick pairs. Notably, these are the only four tRNA base pairs that do not exhibit GoU or UoG pairs in these species.

The long-established nucleotide conservations, or semi-conservations, imposed on tRNA sequences appear primarily in the loops and the portions of the A- and B-boxes in the D- and T-stems (5–8). Much of the variation in tRNAs occurs in the helical stems, but maintains the secondary structure (see Supplementary Figure S1 for some description of the code wheel with some general conservations in tRNA secondary structures). At each base pair position in the stems, four pairs (or six pairs considering GoU pairs) are possible. Thus, for the seven base pair AA-stem, there are close to 16 384 possible combinations (or 280 000 with GoU pairs) and, for the AC-stem with only five base pairs, the possibilities number 1024 (or 7776). Examples where the four types of base pairs occur can be seen on Figure 1 (29:41 or 12:23). The conservation constraints imposed by the B-box (5,6) and the tRNA fold are apparent in base pairs 52:62 and 53:61 (Figure 1).

An important characteristic of tRNA gene families is their diversity in number of loci, even across closely related species and across isoacceptors for the same amino acid (61). Those tRNA genes that share the same anticodon triplet can vary in complexity between species and for different amino acids – some may contain a unique sequence with many multiple exact copies throughout the genome, and others may have many genes with variable sequence differences, each of which may occur at a single or multiple copies. Because all of these share the same anticodon, it is unclear if these variations offer biologically advantageous traits, or are just benign evolutionary noise. We will try to discuss these variations according to their locations, since changes in single-stranded or double-stranded regions, in tertiary pairs or in conserved positions are not expected to have the same impact.

### Number and variations of tRNA genes

**Figure 2.**
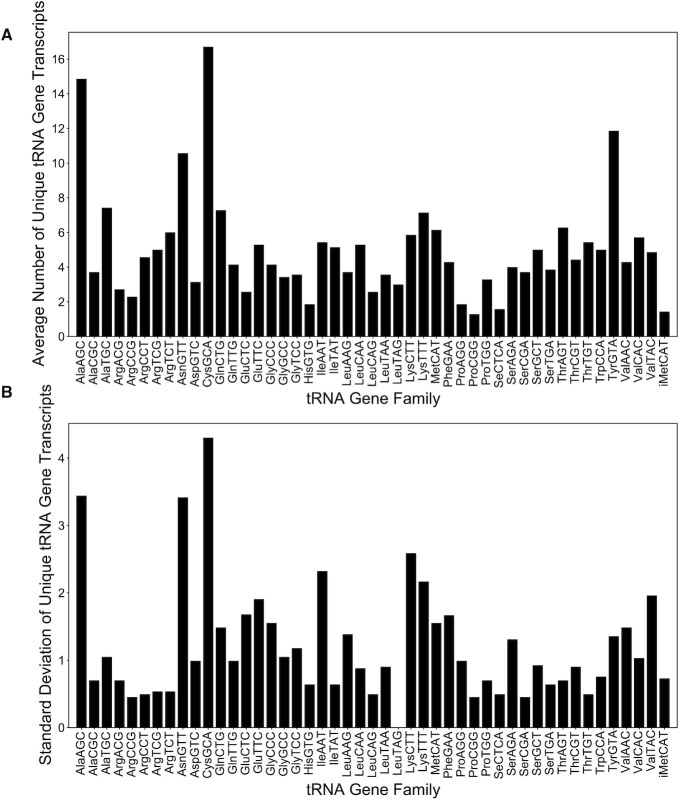
Averages and standard deviations of the number of unique tRNA gene transcripts in primates as deduced from tRNAscan-SE (52). The mean (**A**) and standard deviation (**B**) of the unique high-confidence tRNA gene transcript counts across the reference genomes of seven primate species: *Homo sapiens* (human), *Pan troglodytes* (chimpanzee), *Gorilla gorilla* (gorilla), *Pongo abelii* (orangutan), *Macaca mulatta* (rhesus macaque), *Microcebus murinus* (gray mouse lemur), and *Aotus nancymaae* (Nancy Ma's night monkey). On these plots, only the 47 anticodons expected to be functional in primates are shown, including tRNASeC.

Before focusing on individual tRNA nucleotide features, we first performed a top-level statistical analysis of gene variation among multiple clades to gain context on overall variation among the different isotypes. Within primates, the number of unique tRNA gene transcripts varies significantly with the amino acid type and anticodon, as illustrated in Figure 2A (average counts across species) and 2B (standard deviation of counts across species). Four tRNA isodecoders stand out for the large number of unique genes: tRNA-Cys-GCA (16.7), tRNA-Ala-AGC (14.9), tRNA-Tyr-GUA (11.9) and tRNA-Asn-GUU (10.5). In terms of standard deviation of unique tRNA genes, tRNA-Cys-GCA (4.6) is the highest and tRNA-Tyr-GUA (1.5) the lowest. This analysis shows that even for a fairly closely related group of metazoans such as primates, there is an ever constant, but variable amount of mutational and selective pressure at work.

Alternatively, one may examine total gene copy number, irrespective of the uniqueness of the transcripts they produce. This can capture cases where high gene dosage effect is needed for certain tRNAs to amplify the protein production capacity of the cell. For example, in the silkworm *B. mori*, the numbers of tRNA genes for the tRNAs Ala-AGC, Gly-GCC, Gly-UCC, Asp-GUC are conspicuously high compared to other insects like *Drosophila* species (see Supplementary Table S1). These amino acids are among the main components of silk (62).

### Sequence conservation in the helical stems

Two tRNA families have a conspicuously high number of tRNA genes with the same anticodon triplet in the analyzed phylogeny: tRNA-Ala-AGC and tRNA-Cys-GCA (see Supplementary Table S1). A molecular explanation for such redundancy is as yet unclear. For both tRNA gene families, the 5′ end of the amino acid acceptor (AA) stem is 5′-GGGGR, which is unique among metazoan tRNAs (Table 1). It has been shown that such G-rich sequences promote the formation of intermolecular G quadruplexes at high concentrations for stable small RNAs derived from tRNA-Ala and tRNA-Cys (63). tRNA-Gly-GCC also forms homodimers (64). Additional unknown clade-specific factors should be investigated given that the number of tRNA genes for Cys in *Mammalia* is twice as high as in *Insecta* (see Supplementary Table S1).

**Table 1.** A map of conserved motifs for each stem in each tRNA isotype. The summary is based on the sequence alignments of tRNAs of *H. sapiens*, *M. musculus*, and *B. mori* shown in Supplementary Figure S1. The four main stems of the tRNA secondary structure are abbreviated as follows: the amino acid (AA), the dihydrouridine (D), the anticodon (AC), and the thymine (T) stems. Motifs are shown in the 5′ to 3′ direction for the first strand encountered in the secondary structure, in monospaced font for ease of alignment across rows. All bases follow standard IUPAC conventions, but K is intended to signify ‘G or U’, rather than the standard ‘G or T’. The GGGG(R) motif, specific to only Ala- and Cys-tRNAs, is highlighted in bold. (M) denotes that the motifs are relatively constant across *Mammalia*. Only five tRNAs have four nucleotides in the variable loop: Gly, Glu, Gln, Asp, and His. Leu (with a preference for 5′YYY…GGG3′) and Ser (with a preference for 5′GGG…CCC3′) have a long variable loop with an additional helix (long-arm tRNAs) (see Supplementary Figure S2 where tRNAs with introns are also indicated, the insertion site is always between nucleotides 37 and 38 (1). The amino acids are organized by decreasing number of isoacceptor families with the color code indicating the strength of the codon-anticodon triplet (blue, high GC-content, red, high AU-content, and black in-between). Note that in the 2-codon boxes NNY only one tRNA is used for decoding (with G34 in the anticodon triplet) and in the 2-codon boxes NNR two tRNAs are used (with both C34 and U34 in the anticodon triplet)

Amino acid with anticodon triplet	Isoacceptor families	AA motif	D motif	AC motif	T motif
Ala	3	**GGGG**UUG	GCUC	C..GC	CCGGG
Ala AGC (2 states)		**GGGGR**AU			GYGGG
Gly	3	GCRYUGG	GU.C	…GC	CCGGG
Pro	3	GGCKCGU	GUCU	CUCGC	CCGGG
Val	3	GUUUCCG	GUGU	UYYGC	CCCGG
					CUGGG
Thr	3	GGCGCCG	GCY.	YYKGU	CUGGG
					RCGGG
Arg	3 + 2	G.CC..G	GC.Y	YYKGM	..GGG
Arg ACG		G.GC..G			CYAGG
Leu UAA (M)	3 + 2	ACC.G.A	GCCG	UUGGA	GUGGG
Leu (all others)		G..AG.R	GCCG	YYR..	GUGGG
Ser	3 + 1	G..G..R	GCCG	WUGGA	GYRGG
Glu	2	UCCCW.R	GUCU	CCUGG	CGGCG
Gln	2	GGYYCCA	GUGU	CUGGA	CCGAG
Ile AAU	2	GGGCC.R	GCUC	UGGUG	GCGGG
Ile UAU		GCUCCAG	GCGC	CGGUA	GUGAG
Lys CUU	2	GCCCGGC	GCUC	UGAGA	GUGGG
Lys UUU		GCCCGGA	GCUC	UYRGA	CAGGG
Asp GUC	1	UCYUCGU	GUAU	CCCGC	CGGGG
His GUG	1	GCCGUGA	GUMU	CURCG	CYMGG
Cys GCA	1	**GGGGR**UA	GCUC	WUYGA	CCCGG
Trp CCA	1	GACYYCG	GCGC	UCUGA	GCGUG
Asn GUU	1	GYCUCYG	GCGC	UUCGG	GGUGG
Met CAU	1	GCCYYSK	GCGC	UMAGU	SUGAG
Met^i^ CAU	1	AGCAGAG	GCGC	CUGGG	GRUGG
Phe GAA	1	GCCGAAA	GCUC	UUAGA	CCYGG
Tyr GUA	1	CCUUCGA	GCUC	GWGGA	GCUGG

The first base pair of the amino acid stem is often a recognition element of tRNA aminoacyl synthetases (34,35) and participates in the anchoring of the pre-tRNA to the RNase P complex (65). As expected, only four specific tRNAs lack G1: Asp and Glu have U1-A72, Tyr has C1 = G72 (66), and Met has A1-U72 (67) (Supplementary Figure S3). However, tRNA-Leu-UAA has A1-U72 in *Mammalia* but G1 = C72 for the other four Leu isodecoders, excluding this as a possible aminoacyl transferase recognition element; conversely, *Insecta* uniformly has G1-C72 for all Leu tRNAs (Supplementary Figure S4). In *Bacteria*, Asn and Gln frequently have U1-A72 and Trp has A1-U72 (not shown). Interestingly, the 1:72 base pair is recognized by a direct contact with the RNase P RNA in *Bacteria* (64) and *via* the N-terminal segment of the POP1 protein (a protein subunit of RNase P) in *Eukarya* (65). It is possible that the protein-rich eukaryotic RNase P has a greater latitude in recognition of the 1:72 base pair thus allowing greater sequence drift (66). Because tRNA-Leu-UAA decodes one of the least-used Leu codons (TTA), this may hint at U1-A72 as a distinguishing regulatory feature for this isodecoder. Regardless, the biological basis for the transition to U1-A72 for Leu-UAA in mammals and other vertebrates (data not shown) is an intriguing question.

While the above sequence motifs are unique to specific isotypes or clades, other tRNA families have highly conserved stem motifs (Table 1). These are found in isoacceptor families with variations within isoacceptor families (e.g. Ala, Arg, Leu, Ile) or between isodecoders (as marked in Table 1 by Y, R, M, K, W, S). However, the conservation between human and silkworm is striking: 5′-GUUUCCG for the AA-stem in all isoacceptors of tRNA-Val and 5′-GGYYCCA for all isoacceptors of tRNA-Gln. The restricted variations in the dihydrouridine (D) stem can be in part explained by the A-box internal promoter for RNA polymerase III (Pol III) (5,6) and the tertiary pairs (see below). Even in the AC-stem, there are several conserved motifs associated with specific isodecoder families. For example, 5′-CCCGC is specific to tRNA-Asp and 5′-CCUGG is specific to tRNA-Glu. The last pair of the T-stem (always G53 = C61) is constrained by the B-box internal promoter and the three-dimensional fold of the T-loop (17), but the first four nucleotides of the 5′ strand of the T-stem are not constrained and again they are typical of a given amino acid and highly conserved. For example, in tRNA-Asn, the T-stem motif is 5′-GGUGG and, in tRNA-Tyr, 5′-GCUGG (Table 1). Also, the last three residues of the 5′-strand of the T-stem are often a series of three Gs in the strong anticodon-codon pairs (the Northern side of the genetic code wheel) and more often a series of two Gs in the Southern part. Even the additional helix in long-arm tRNAs (YYY…GGG in Leu and GGG…CCC in Ser) present unusually strong conservation throughout the genomes and clades of the three species analyzed in depth (Table 1, Supplementary Figure S2), relative to analogous observations in bacteria and yeast.

### Non-Watson–Crick pairs in helical stems

**Figure 3.**
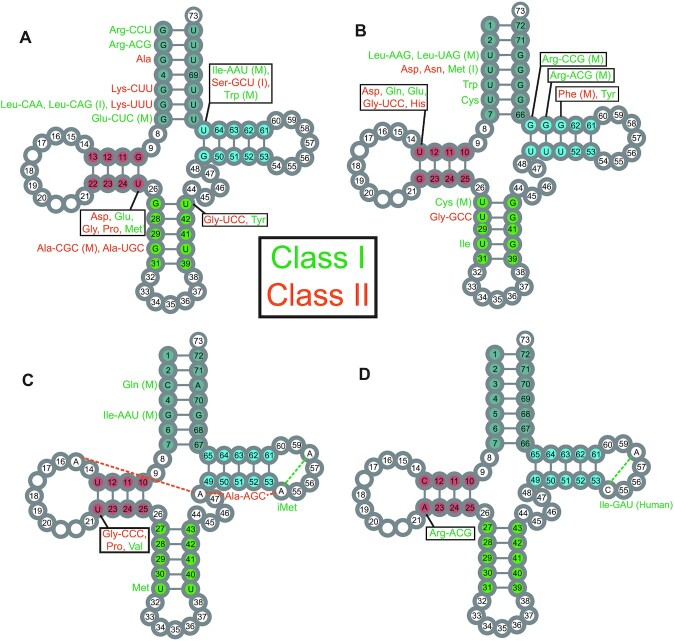
Summary of GoU base pairs and other non-Watson–Crick base pairs in helical stems of tRNAs. Isotype- and position-specific conserved GoU (**A**) and UoG (**B**) base pairs in tRNA stems, as well as other non-Watson–Crick pairs (**C, D**) are shown by Sprinzl position. The long-arm tRNAs are characterized by a G13oA22. (**C**) In the AA-stem, Gln-UUG tRNAs present often in *Mammalia* a CoA opposition at positions 3:70 and Ile-AAU tRNAs have GoG at positions 5:68. Gly-CCC, Pro, and Val tRNAs have a UoU at positions 13:22 in the D-stem. In addition, Ala-AGC tRNAs have an RoA tertiary interaction at positions 15:48 together with A54oA58 (47), and Met tRNAs have AoA at positions 54:58. (**D**) Arg-ACG tRNAs have a CoA at positions 13:22 in the D-stem, and *H. sapiens* Ile-GAU tRNAs have the unusual CoA tertiary interaction at positions 54:58 in the T-loop. Numbers in stem positions indicate that no non-Watson–Crick interactions are observed at that position in insect or mammalian tRNAs (except for tRNA-Thr at positions 4:69 where both GoU and UoG are observed). Amino acids are colored based on aminoacyl tRNA synthetase class (Class I in green and Class II in orange). (M) indicates that the base pair is observed in *Mammalia* and (I) indicates that the base pair is observed in *Insecta* (all valid for *Drosophila* species). Isodecoders without either letter are conserved in both insects and mammals.

The most frequently observed non-Watson–Crick pair in helical stems of structured RNAs is the wobble GoU. Each of the four helical stems may contain a GoU pair. Of the total 21 base pairing positions found in helical stems (7 + 4 + 5 + 5, for the amino acid (AA), the dihydrouridine (D), the anticodon (AC), the thymine (T) stems, respectively), 15 base pairing positions present GoU pairs (Figure 3A, B). Thus, out of 30 possibilities for a GoU or UoG pair, we found 10 positions with GoU in one or more tRNAs and 11 positions with at least one UoG; and of these, six positions had both GoU or UoG pairs (Figure 3). Some base pair positions do not show any GoU pairs: 11:24 and 12:23 in the D-stem, 29:41 in the AC-stem, and 53:61 in the T-stem (Figure 1). Notably, position 29:41 interacts with the rRNA in the P-state, and a Watson–Crick base pair is highly favored (68–70). Note that uridines in the AC-stem are generally modified into pseudouridines and this modification does not prevent formation of the wobble pair (37,71). The pair 53:61 in the T-stem does not form a GoU pair, presumably due to structural (17) and B-box constraints (5,6). Two other positions present a GoU pair rarely and, then, not specifically attached to a tRNA type: 52:62 and importantly the last base pair of the AC-stem, 31:39.

Structurally, a GoU pair is not equal to a UoG pair (45) and, depending on the amino acid, the occurrence *and* orientation of a GoU pair may be conserved throughout an isoacceptor family. Importantly, a GoU pair may be conserved in position and orientation in only a subset of the isodecoders of an isoacceptor family. Nine base-pairing positions occur in a single orientation (Figure 3A, B): these comprise four in the D-stem (G10oU25, U13oG22) and T-stem (U50oG64, U51oG63) with one in the AC-stem (U28:G42) and four in the AA-stem (G1oU72, G2oU71, U4oG69, G7oU66). Six pairs show both orientations of GoU pairs: three in the AA-stem (3:70, 5:68, 6:67), one in the T-stem (49:65), and two in the AC-stem (27:43, 30:40).

For the D-stem, the internal A-box promoter for Pol III transcription may restrict the alternate GoU pairs (5,6). In the T-stem, interactions with elongation factor Tu may also restrict alternate GoU pairs (72). The GoU pairs in the D- and T-stems are shared among several amino acids. Interestingly, U51oG63 is found in mammalian tRNA-Phe and tRNA-Tyr, two close amino acids not easily distinguished (73). Conversely, in the AA- and AC-stems, a given GoU orientation is attached to a specific amino acid. For example, in the AA-stem, there is the well-known identity element G3oU70 for Ala (74,75), as well as U3oG70 for Leu-NAG and G6oU67 for Leu-YAA, U5oG68 for Trp, and U6oG67 for Cys. In the AC-stem, we observe G27oU43 for Tyr and tRNA-Gly-UCC, U27oG43 for Cys, G30oU40 for Ala and tRNA-Arg-UCU(with intron), and U30oG40 for Ie (Figure 3A). In bacteria, the nature of the 27:43 pair has been correlated with the accommodation of a non-Watson–Crick base pair at the first codon:anticodon triplet position (76). Finally, in the AC-stem, two pairs, 29:41 and 30:40, are recognized by the ribosome during translocation at the P-state (68–70). As shown in Figure 3A and B, 29:41 is overwhelmingly a complementary Watson–Crick, while 30:40 does occur as either a GoU or UoG pair in tRNA-Ala-YGC and tRNA-Ile. Also, Arg-UCU tRNAs present a G30oU40 pair when the transcript contains an intron (in absence of intron, like in *B. mori*, it is a G30 = C40 like in all *Drosophila*). Because the pairs 29:41 and 30:40 interact with ribosomal elements in the P-state (68), a GoU pair at 30:40 may play a role during translocation (see discussion in (47)). However, a comparison between yeast tRNA-Asp free and complexed with its cognate aminoacyl synthetase show that deviations between the tRNAs occur at a hinge point formed by the yeast-specific G30oU40 pair in the AC-stem (77,78). Interestingly, the suppression efficiency of the yeast amber tRNA-Ile in *E. coli* is modulated by the presence of U30oG40 (79). Further, the yeast amber tRNA-Ile is charged by bacterial glutaminyl and lysyl tRNA synthetases and the G30oU40 mutant only by LysRS (79).

Other non-Watson–Crick pairs are conserved in the tRNA stems (Figure 3C, D), with none in the T-stem, one at one end of the D- and AC-stems, and two in the AA-stem. Except for U13oU22 (which occurs in Gly-CCC, Pro, Val), these non-Watson–Crick pairs are attached to a family or sub-family of isodecoders. *Homo sapiens* tRNA-Ile-GAU displays the very unusual C54oA58 opposition in the T-loop (other cases include *D. willistoni* and five among eight genomes in *Primates*). tRNA-Ile-AAU also presents a G5oG68 pair in *Mammalia*. Met-tRNAs present a U31oU39 pair while Met^i^-tRNAs have a A54oA58 pair with a C33 residue instead of the highly conserved U33 (80–82).

### GoU base pairs in tRNAs are unlikely to be evolutionary intermediates

**Figure 4.**
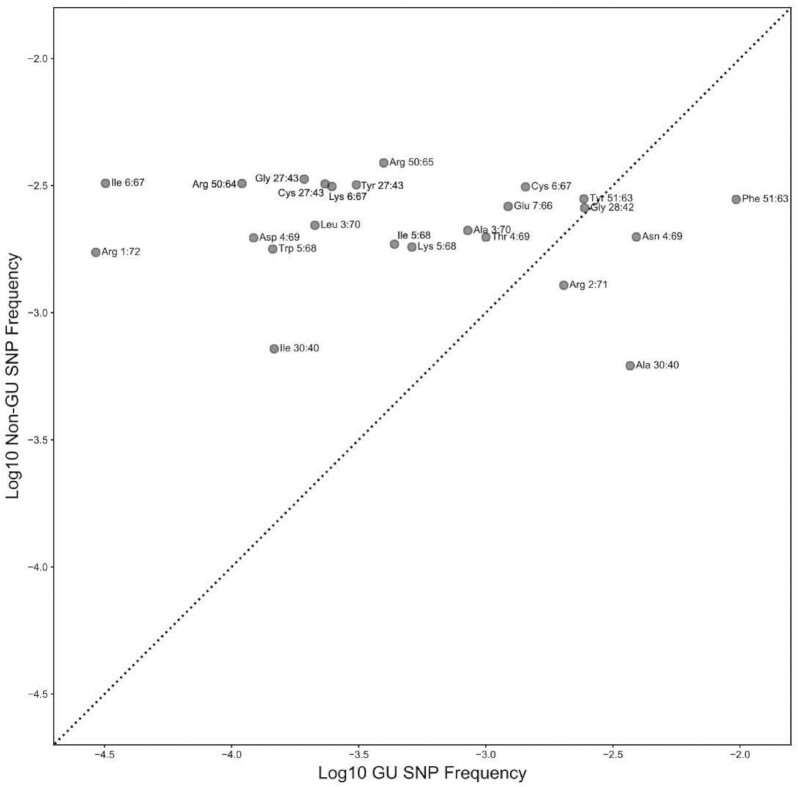
Polymorphisms disrupting GoU base pairs reach lower frequencies than other SNPs at identical positions. At each GoU position listed in Figure 3, we compare the average allele frequency for SNPs disrupting GoU base pairs to the average allele frequency of SNPs at the same Sprinzl positions across all other tRNAs. We find that for 20 of 24 comparisons, SNPs disrupting GoU base pairs reach a lower frequency (sign test, *P* < 6.3 × 10^–4^) and are above the diagonal line shown here. We used data from dbSNP for these comparisons (see Methods) (55).

The occurrence of non-Watson–Crick base pairs in helical stems of functional RNAs is not surprising in itself, and such pairs are regularly observed in sequence alignments of many RNAs, as in ribosomal RNAs (see for analysis (83,84)). In the evolution of RNA molecules, GoU pairs are often considered as intermediates between G = C and A–U pairs (or vice versa). However, this is not likely to be the case for the GoU pairs described here in tRNAs. When conserved throughout a large phylogeny, a given non-Watson–Crick pair most likely harbors a folding constraint or a key point of contact with an interacting partner molecule. Consistent with this idea, we find that single nucleotide polymorphisms (SNPs) in human tRNA genes that disrupt GoU base pairs for each isotype and position shown in Figure 3A and B reach lower frequencies on average than SNPs disrupting non-GoU base pairs at the same positions in other isotypes (sign test, *P* < 6.3 × 10^–4^, Figure 4), based on human population data from dbSNP (55) (see Materials and Methods). Similarly, isotype-specific GoU base pairs in tRNA stems have higher phyloP scores across seven primate genomes than non-GoU base pairs at the same positions (sign test, *P* = 0.202) (53,59,85). Although this test is not statistically significant, the observation that GoU base pairs are *more* conserved than non-GoU pairs at the same positions indicates that these GoU base pairs are unlikely to be transient.

### Are the GoU pairs correlated with other tertiary or critical pairs?

To analyze structural consequences of the molecular signatures associated with each isotype, we suggested an organization of the genetic code according to the strength (or free energy of the triplet minihelix) of the codon/anticodon triplet that must form in the ribosomal decoding site translation (86). The code is represented as a wheel with the strong triplets in the North and the weak triplets in the South regions. Such a representation displays the ‘oldest’ amino acids in the North and the more highly modified tRNA anticodons in the South region. This representation stresses the point that the free energy of triplet formation encompasses several complex interactions and contributes to our understanding of decoding in translation.

**Figure 5.**
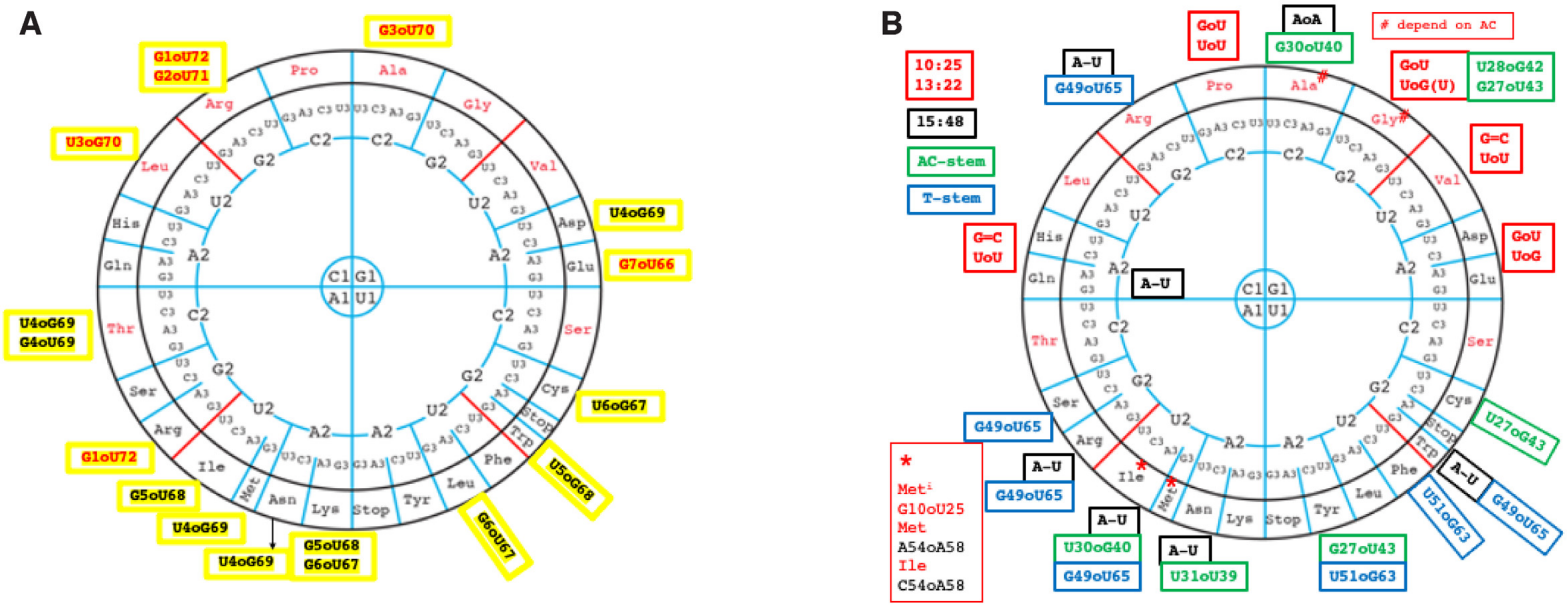
Distribution along the code wheel of GoU pairs. The triplets are organized from the center to the periphery so that the GC-rich codon:anticodon triplets are at the top (North) and the AU-rich codon:anticodon triplets at the bottom (South) of the wheel. Along the (red-line) diagonals are those triplets with intermediate energies (86). (**A**) The GoU pairs occurring in the AA-stem are shown next to the amino acid and those in red occur only in tRNAs specific for that amino acid. (**B**) The GoU pairs occurring in the D- (red), AC- (green) and T-stems (blue). Positions 15:48 (black) are G = C, except where indicated. When the specificity is attached to an isodecoder subfamily it is indicated in red.

Figure 5A shows the distribution of the GoU pairs, for the AA-stem and the other three stems respectively, around the code wheel (Figure 5B). For the AA-stem, there are many more variations in the South part than in the North. Also, GoU pairs specifically attached to an amino acid are all in the North region (Arg is a six-codon box with a single aminoacyl tRNA synthetase (aaRS)). The GoU pairs in the D-stem occur only in the North or GC-rich region. The variations in the other stems are more frequent in the South part, with more diversity for Ala and Gly in the North. Interestingly, the tertiary A15oU48 pair occurs frequently with G49oU65, with both being very close to each other in the folded tRNA (Figure 5B).

Several positions in the tRNA architecture are key for tRNA folding, recognition of protein cofactors, or stability of the codon-anticodon triplet in the decoding site. Exceptions to these conservations can be observed in annotated tRNA genes, but they generally occur together with other point mutations, have low tRNAscan-SE bit scores, and most likely correspond to pseudogenes. We note some exceptions, such as C33 instead of U33 in tRNA-Met^i^. Residue 9 is always a purine (R9), except in tRNA-His where it is a C9 (residue 9 precedes the invariant G10 that starts the D-stem and forms a triple with 12:23 of the D-stem).

Several non-Watson–Crick pairs are key to the maintenance of the function or folding of the tRNA (17) and conservations are expectedly observed. In the D-loop, A14 interacts with U8 and A21. The loop-loop interactions between the D- and T-hairpins are maintained through the conserved G18G19 in the D-loop and U54, Ψ55, C56 in the T-loop. All these nucleotides and contacts are conserved in all analyzed tRNAs. Interestingly, other tertiary contacts where variations may occur stay identical for each tRNA isotype, as observed above for the secondary structure pairs (see Sup_Data_1_Align.docs and Sup_data_2_2D_Struct). Some exceptions are: (i) the pair 13:22 varies between the isoacceptor families of tRNA-Arg, tRNA-Gly, and tRNA-Thr; (ii) the long-range pair between residues 15 and 48 is always either G15oC48 or A15oU48 in all tRNAs of an isoacceptor family, except in the subclass of tRNA-Ala-AGC containing a A54oA58 where A15oA48 is also present (Figure 3C, D), and in tRNA-Ile-RAU (G15oC48) and tRNA-Ile-UAU (A15oU48). Also, there are three exceptions to the internal *trans* Watson–Crick/Hoogsteen pair U(T)54oA58: there is an A54oA58 in a class of tRNA-Ala-AGC and in all tRNA-Met^i^ sequences, and a C54oA58 pair in Ile-GAU for several primates.

## DISCUSSION

Here, starting from a systematic alignments of tRNA genes in *H. sapiens*, *M. musculus*, and *B. mori*, we extended the analysis to *Mammalia* and *Insecta*, although the great majority of what is known biochemically and molecularly is based on studies on *Bacteria* and *Archaea* (e.g. (82)). We have also excluded eukaryotic microbial species. There is a great diversity in tRNA and code variations in microorganisms (87–89), in contrast to the conservation among mammals and insects described here. We have observed several conserved isotype-specific motifs in these genomes that were extended and often generalized to other genomes within *Mammalia* and *Insecta* (especially *Drosophila* species), indicating that sequence motifs discussed here are not species-specific but often clade-specific or more deeply conserved.

We show that each of the four tRNA stems contains at least one GoU pair with conservation in positions that depends on the amino acid specificity of the tRNA and that, of the total 21 usual stem pairs, only four pairs never present a GoU pair: the two middle ones in the D-stem; the middle one in the AC-stem; and the last pair in the T-stem. Of the seven GoU pair positions that are amino acid specific, four are attached to a single amino acid (G3oU70 for Ala, U5oG68 for Trp, U6oG67 for Cys, U30oG40 for Ile), which could point to a role as a molecular identity element for their cognate amino acid tRNA synthetases, as is established for the G3oU70 in the Ala system (74,90). The U4oG69 pair occurs in tRNA-Asp and tRNA-Asn could also be part of the synthetase identity elements. The three other pairs are shared between some amino acids (G10oU25, U13oG22, U4oG69). For the first two in the D-stem, only a single orientation for each of the two GoU pairs is found (G10oU25 and U13oG22); they occur in tRNAs with specific amino acids corresponding to class II tRNA synthetases (Gly, Pro, Asp, His) or class I tRNA synthetases (Glu, Gln, Met^i^)), which includes the five tRNAs with 4 nts in the V-loop (Gly, Asp, Glu, His, Gln). This type of conservation could be instead related to the tight structural fold of the core of the tRNAs. However, for the six pairs that show both orientations of the GoU pairs (AA-stem, 3:70, 5:68, 6:69, T-stem, 49:65, and AC-stem, 27:43, 30:40), each orientation of the GoU pair is attached to a different amino acid, and such GoU pairs could be synthetase identity elements. Indeed, the position of a GoU pair at the ends of a helix maximizes the long-distance effect from the change in twist introduced by the GoU pair. In addition, the angle of change is different for GoU and UoG, which leads to additional molecular discrimination. Further, in mammals nine aminoacyl tRNA synthetases (Arg, Asp, Gln, Glu, Ile, Leu, Lys, Met, Pro) form a multi-synthetase complex (91,92) and the corresponding tRNAs are among the most frequent ones carrying a conserved GoU pair.

Interestingly, some GoU pairs are anticodon-specific throughout *Mammalia* and *Insecta*, while some are restricted to a clade. Among the first category are Arg and Leu which, as they belong to the 6-codon boxes, demand subtle recognition by their cognate synthetases (34,93). However, some anticodons of Ala, Gly, and Lys are also in that category. Additional conservation of GoU pairs occurs in an anticodon-specific fashion in *Mammalia*. The roles of such conservations are difficult to understand but they could indicate that some tRNAs have specialized functions either within the translation process or outside like aminoacyl tRNA synthetases do (41,94–96). Still, some tRNAs, specific for certain amino acids, do not present any conserved GoU pairs: Gly-CCC, Val of class I (but both with U13oU22), the Ser 4-codon box YCN of class II (but with G13oA22 and long variable loop, Supplementary Figure S2), and Arg-ACG with C13oU22.

Conservation in tRNA sequences is driven by the free energy of all intra- and inter-molecular interactions made by tRNAs during their biological functions. These functional contacts occur sequentially: the maturation and modification enzymes, the aminoacyl tRNA synthetases, the elongation factors, and the ribosomal grips in the three states of the translation process. The sequentiality of the interactions could imply that a particular tRNA-protein complex forms an interaction bottleneck (like the rate-limiting step in enzyme kinetics) that shapes some elements of the tRNA sequence. However, importantly, the molecular recognition modes are different in the various sequential states. This is especially noticeable when similar tRNA regions are recognized by different interactants: the whole range of physico-chemical interactions is taken advantage of and in a differential manner. The A- and B-boxes of the Pol III promoters are transcribed as linear (DNA) sequences by the polymerase but fold in the conserved T-loop with precise contacts between conserved nucleotides of the T- and D-loops in the three-dimensional tRNA architecture (17,37). Within the RNase P complex, the 1:72 base pair is recognized by a direct RNA-RNA contact in *Bacteria* (64) and *via* a protein subunit in *Eukarya (**65**)*. In the -CCA end or the anticodon loop regions, the molecular recognition modes by the synthetases and the ribosomal recognition sites are not identical: sequence-guide base pairing dominates in the ribosome (61), while multiple and various contacts with amino acid side chains and peptide linkages occur in the synthetases (93). Similarly, the ribosome interacts with pre-organized anticodon loops and with -CCA ends where base stacking is extensive (38–40,68), while the synthetases often distort and destructure the anticodon loop to access directly the identity elements of the bases (34,93,97–99). Further, the pair 30:40 interacts with ribosomal elements in the P-state, and could play a role in translocation (68–70) and it has been involved in recognition by aminoacyl tRNA synthetases (77,79). Additionally, as discussed above, because a GoU pair induces a variation in helical twist within a helical fragment, direct contacts between the GoU pair and the protein or RNA ligand are not required for measurable effects on the binding efficiency (as in the paradigmatic case of Ala (74,90)). In the end, the variation in the distribution of conserved nucleotides and GoU pairs point to necessary but subtle trade-offs between the multiple and diverse tRNA molecular recognition events (19), which suggests that despite the singularity of each tRNA, globally they all tend to behave similarly, as shown for Pol III (100).

Conversely, deviations in tRNA sequences compared to standard conservations may indicate an alternative pathway, or a specific bias, during the translation processes in which tRNAs are involved. To assess that possibility, the establishment of ‘expected conservation’, with restraints on the lineages, is therefore a prerequisite. As discussed above, the many cases of conservation described here reflect the many contacts between tRNA molecules and their multiple interacting partners during maturation and translation, as well as for additional biological functions (41,101). Indeed, some deviations, especially among isodecoders, stand out (e.g. Ala, Gly) and may indicate the existence of ‘specialized tRNAs’ for specific translation or alternative functions, such as one tRNA-Arg that is involved in N-terminal arginylation (102), tRNAs that produce regulatory tRNA-derived small RNAs (103–105) or the brain-essential tRNA-Arg-UCU which is impaired by a G50 = C64 mutation to a GoU pair, causing ribosome stalling that can lead to neurodegeneration in mice (106). In bacteria, aminoacyl tRNAs are involved in nonribosomal biosynthesis (peptidoglycan (107), natural products (108), lipid modification (109,110), or protein degradation (111)); however, these unfrequently require special dedicated tRNA sequences (107). Finally, considering the high mutation rates of tRNA genes (42,53), the GoU conservations described here may be also useful for identifying differential tRNA gene expression during normal cell differentiation (112) or better recognizing damaging and disease-prone variants in humans (113–115).

## DATA AVAILABILITY

The datasets generated during and/or analyzed during the current study are in the Supplementary Data or available from the corresponding author.

## Supplementary Material

gkac222_Supplemental_Files
