# Supplementary material for: Eukaryotic tRNA sequences present conserved and amino acid-specific structural signatures
Source: Nucleic Acids Res. 2022 Apr 5;50(7):4100–12. doi: 10.1093/nar/gkac222 (PMC9023262; doi:10.1093/nar/gkac222)
Supplement: gkac222_Supplemental_Files [file gkac222_supplemental_files.zip › Revised2 Sup Mat.docx]

**Title:**

**Eukaryotic tRNA sequences present conserved and amino acid-specific structural signatures**

**Authors:**

Eric Westhof^1^, Bryan Thornlow^2,3^, Patricia P. Chan^2,3^, Todd M. Lowe^2,3^

**Affiliations:**

1. Université de Strasbourg, Institut de Biologie Moléculaire et Cellulaire, *Architecture et Réactivité de l’ARN*, CNRS UPR 9002, 2, allée Konrad Roentgen, F-67084 Strasbourg (France)
2. Department of Biomolecular Engineering, Baskin School of Engineering, University of California Santa Cruz, Santa Cruz, CA 95064, USA
3. UCSC Genomics Institute, University of California Santa Cruz, Santa Cruz, CA 95064, USA

**Supplementary figures**

**Figure S1:** The structural alignments of the tRNAs from *H. sapiens*, *M. musculus*, and *B. mori* are organized by decreasing tRNAscan-SE bit scores and by anticodon triplets (separate file Supplementary_Data_1_Align.docx). The cloverleaf structures of the 2D structures of the tRNAs based on the alignments are given in the separate file Supplementary_Data_2_2D_Struct.pptx.

**
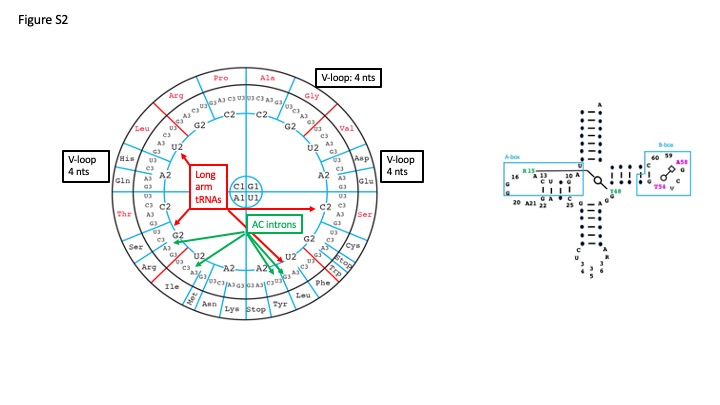
**

**Figure S2:** (Left) Some elements of the conserved secondary structure of tRNAs in the eukaryotic genomes studied are represented within the code wheel [(86)](https://paperpile.com/c/Ov53aw/KkoRx). In this representation, the codon triplets are organized from the center to the periphery so that the GC-rich codon:anticodon triplets are at the top (North) and the AU-rich codon:anticodon triplets at the bottom (South) of the wheel. Along the diagonal are those triplets with intermediate energies [(86)](https://paperpile.com/c/Ov53aw/KkoRx). The V-loop is between the 3’end of the AC-stem and the 5’end of the T-stem. In most tRNAs, it comprises five nucleotides, except in the five tRNAs indicated on the coding wheel (black). Two of the six-codon tRNAs, Leu and Ser, have a very long V-loop forming an additional helix (red). Four tRNAs have intronic sequences in the AC-loop (between 37 and 38). (Right) Regions on the cloverleaf 2D structure of the pol III promoter elements A- and B-boxes (enclosed in blue lines). In hemiascomycetes, residue 21 is included in the A-box and residue 52 is excluded [(7)](https://paperpile.com/c/Ov53aw/L2qvG).


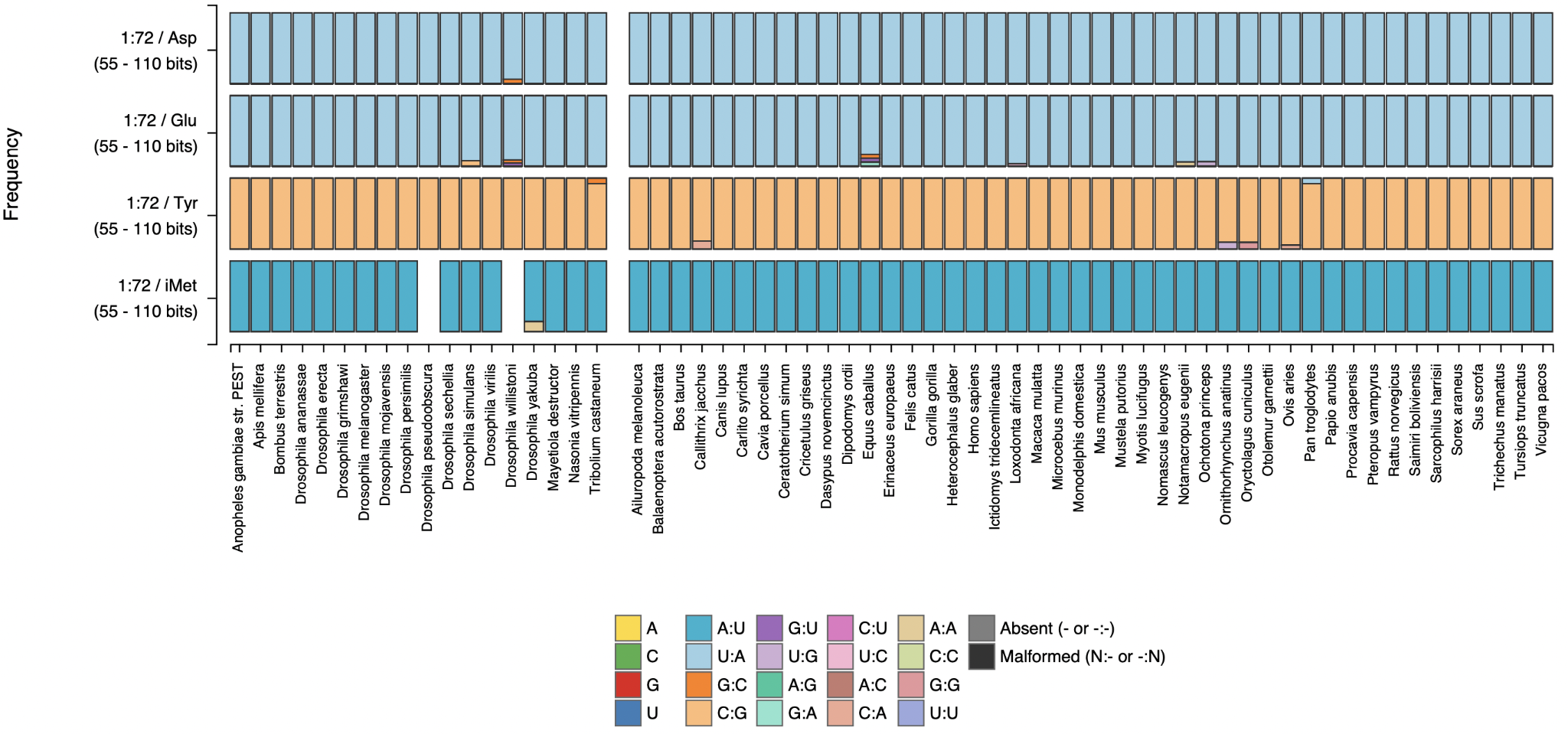


**Figure S3:** Extracts from tRNAviz [(49)](https://paperpile.com/c/Ov53aw/xaRV) showing the conservation for base pair 1:72 in tRNAs from *Insecta* and *Mammalia*.

**
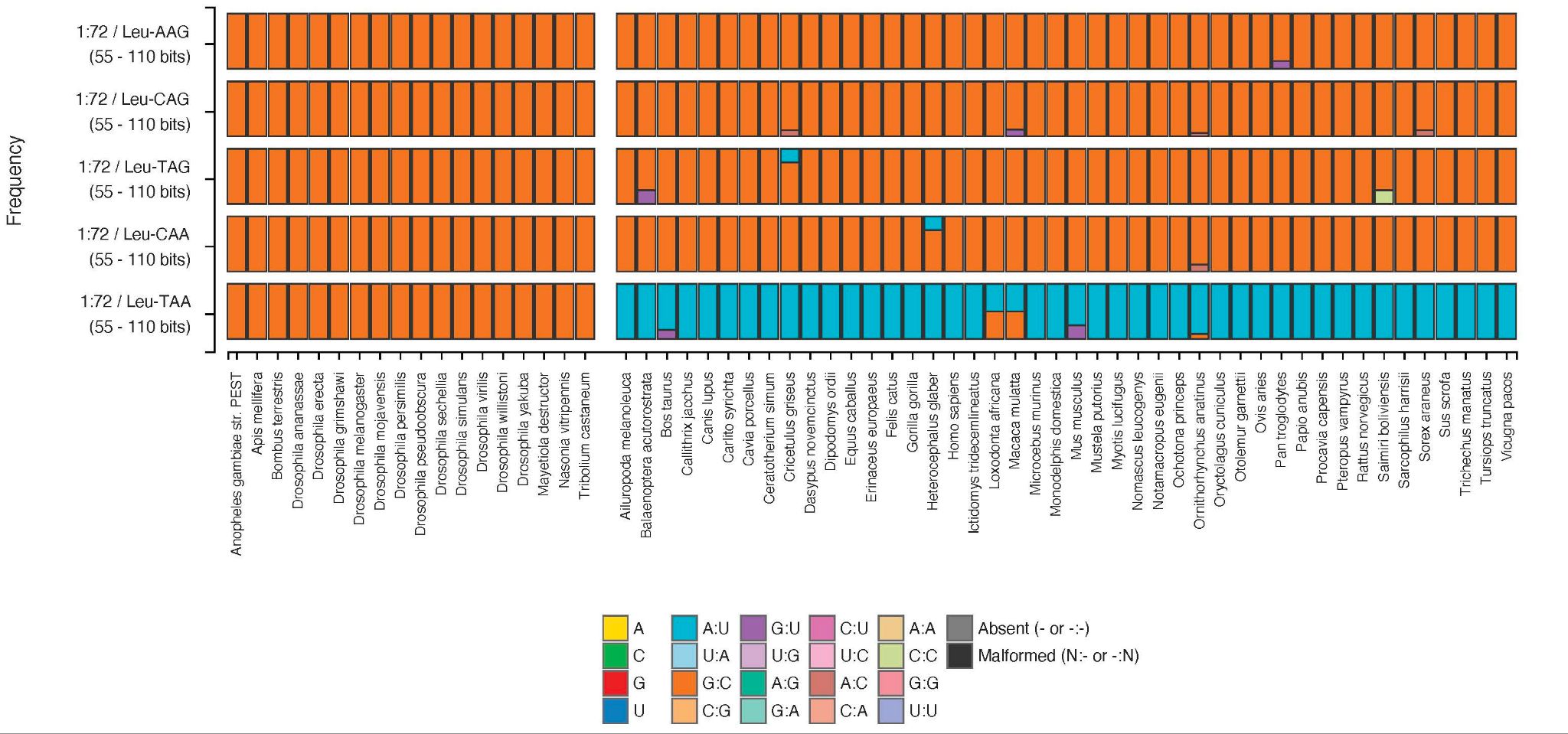
**

**Figure S4:** Extracts from tRNAviz [(49)](https://paperpile.com/c/Ov53aw/xaRV) showing the conservation for base pair 1:72 in tRNA-Leu from *Insecta* and *Mammalia*.

|  | *H.s./M.m./B.m.(D.m.)* | | | *H.s./M.m./B.m.(D.m.)* | | | *H.s./M.m./B.m.(D.m.)* | | |
| --- | --- | --- | --- | --- | --- | --- | --- | --- | --- |
| Amino acid | Number of anticodons  for amino acid | | | Total number of tRNA  genes | | | Nbr of isodecoder  families | | |
| Ala-AGC | 3 | 3 | 3 | 8 | 5 | - | 6 | 4 | - |
| Ala-AGC* |  |  |  | 17 | 6 | 28 (12) | 8 | 4 | 5 (2) |
| Ala-UGC |  |  |  | 8 | 11 | 10 (2) | 6 | 7 | 2 (2) |
| Ala-CGC |  |  |  | 4 | 3 | 6 (3) | 3 | 3 | 2 (1) |
| Arg4-ACG | 3 | 3 | 2 | 7 | 6 | 8 (10) | 2 | 3 | 3 (1) |
| Arg4-UCG |  |  |  | 6 | 5 | 8 (5) | 6 | 4 | 2 (3) |
| Arg4-CCG |  |  |  | 4 | 3 | - (-) | 2 | 1 | -(-) |
| Gly-GCC | 3 | 3 | 3(2) | 14 | 13 | 30 (13) | 5 | 4 | 4 (1) |
| Gly-UCC |  |  |  | 9 | 7 | 15 (7) | 3 | 1 | 5 (2) |
| Gly-CCC |  |  |  | 5 | 5 | 3 (-) | 3 | 3 | 1 (-) |
| Pro-AGG | 3 | 3 | 3 | 9 | 6 | 9 (7) | 2 | 1 | 2 (1) |
| Pro-UGG |  |  |  | 7 | 5 | 4 (5) | 3 | 2 | 1 (1) |
| Pro-CGG |  |  |  | 4 | 3 | 4 (5) | 2 | 1 | 2 (2) |
| Leu4-AAG | 3 | 3 | 3 | 9 | 5 | 6 (4) | 3 | 3 | 1 (1) |
| Leu4-UAG |  |  |  | 3 | 3 | 3 (2) | 3 | 1 | 2 (1) |
| Leu4-CAG |  |  |  | 9 | 10 | 6 (8) | 2 | 4 | 1(1) |
| Val-AAC | 3 | 3 | 4(3) | 8 | 7 | 9 (6) | 3 | 5 | 3 (2) |
| Val-UAC |  |  |  | 4 | 3 | 6 (2) | 3 | 1 | 1 (1) |
| Val-CAC |  |  |  | 12 | 8 | 7 (7) | 5 | 3 | 4 (2) |
| Val-GAC |  |  |  | - | - | 1 (-) | - | - | 1 (-) |
| Ser4-AGA | 3 | 3 | 3 | 9 | 7 | 9 (8) | 3 | 2 | 2 (3) |
| Ser4-UGA |  |  |  | 4 | 3 | 7 (6) | 1 | 2 | 3 (2) |
| Ser4-CGA |  |  |  | 4 | 3 | 4 (4) | 1 | 3 | 2 (1) |
| Thr-AGU | 3 | 3 | 3 | 9 | 9 | 8 (8) | 6 | 7 | 4 (2) |
| Thr-UGU |  |  |  | 6 | 4 | 4 (6) | 6 | 3 | 1 (2) |
| Thr-CGU |  |  |  | 4 | 4 | 3 (3) | 4 | 4 | 1 (1) |
| Gln-UUG | 2 | 2 | 2 | 6 | 5 | 7 (4) | 4 | 4 | 3 (2) |
| Gln-CUG |  |  |  | 12 | 9 | 7 (8) | 5 | 3 | 2 (4) |
| Glu-UUC | 2 | 2 | 2 | 8 | 8 | 14 (6) | 5 | 3 | 3 (1) |
| Glu-CUC |  |  |  | 8 | 10 | 11 (13) | 2 | 2 | 6 (3) |
| Arg2-UCU^i^ | 2 | 2 | 2 | 4 | 4 | 3 (3) | 4 | 4 | 1 (3) |
| Arg2-CCU |  |  |  | 5 | 5 | 6 (3) | 5 | 4 | 2 (1) |
| Ile-AAU | 3 | 2 | 2 | 14 | 11 | 17 (9) | 7 | 4 | 7 (1) |
| Ile-GAU(H.s.) |  |  |  | 3 | - | -(-) | 1 | - | - |
| Ile-UAU^i^ |  |  |  | 5 | 4 | 6 (2) | 5 | 4 | 4 (1) |
| Lys-UUU | 2 | 2 | 2 | 10 | 9 | 9 (6) | 6 | 3 | 2 (1) |
| Lys-CUU |  |  |  | 9 | 10 | 10 (13) | 4 | 3 | 1 (1) |
| Leu2-UAA | 2 | 2 | 2 | 4 | 4 | 6 (4) | 4 | 4 | 2 (4) |
| Leu2-CAA^i^ |  |  |  | 5 | 4 | 7 (4) | 5 | 4 | 5 (3) |
| Asp-GUC | 1 | 1 | 1 | 13 | 15 | 31 (12) | 3 | 3 | 10 (1) |
| His-GUG | 1 | 1 | 1 | 9 | 10 | 14 (5) | 1 | 3 | 1 (1) |
| Ser2-GCU | 1 | 1 | 1 | 7 | 7 | 9 | 5 | 5 | 3 (2) |
| Cys-GCA | 1 | 1 | 1 | 27 | 22 | 11 (7) | 19 | 8 | 4(3) |
| Trp-CCA | 1 | 1 | 1 | 7 | 7 | 9 | 5 | 5 | 1 (2) |
| Tyr-GUA^i^ | 1 | 1 | 1 | 18 | 10 | 12 | 7 | 7 | 3 (3) |
| Met-CAU | 1 | 1 | 1 | 11 | 10 | 10 (6) | 6 | 6 | 3 (2) |
| Met^i^-CAU | 1 | 1 | 1 | 9 | 9 | 12 (6) | 2 | 3 | 4 (2) |
| Asn-GUU | 1 | 1 | 1 | 24 | 12 | 22 (10) | 17 | 4 | 6 (1) |
| Phe-GAA | 1 | 1 | 1 | 8 | 7 | 8 (8) | 3 | 3 | 3 (1) |
|  | 47 | 46 | 46(44) | 414 | 344 | 421 (290) |  |  |  |

**Table S1**: Distribution of the number of anticodons for each coded amino acid, the total number of tRNA genes for each codon and the number of isodecoder families in *H. sapiens*, *M. musculus*, *B. mori* (and partially *D. melanogaster*). In the table, AGC* corresponds to those tRNA-Ala-AGC with an unusual pair at either positions 15:48, 30:40, or 54:58 (see Table 1 in [(47)](https://paperpile.com/c/Ov53aw/64TCx)). Arg(2)-UCUi means that in the 2-codon box of Arg, tRNA-UCU has an intron. The sequence variations in introns contribute to the number of isodecoders. The amino acids highlighted in blue form GC-rich codon:anticodon and in red AU-rich codon:anticodon. They are ranked according to the number of different anticodon triplets used for that amino acid. Large numbers (some of which are discussed in the main text) are in green.
