# Supplementary material for: Eukaryotic tRNA sequences present conserved and amino acid-specific structural signatures
Source: Nucleic Acids Res. 2022 Apr 5;50(7):4100–12. doi: 10.1093/nar/gkac222 (PMC9023262; doi:10.1093/nar/gkac222)
Supplement: gkac222_Supplemental_Files [file gkac222_supplemental_files.zip › Sup_Data_1_Align.docx]

*H.sapiens*

*M.musculus*

*B.mori*

>>>>>>>..>>>>........<<<<.>>>>>.......<<<<<.....>>>>>.......<<<<<<<<<<<<.

5’AA...**U**.5’D.**A**...**GG**.A3’D..5’AC..**U**.AC...3’AC......5’T.TP......3’T...3’AA.

**8** **1**1 2 2 **3**333 4 4 5 5 6 6 7

**4**5 1 6 **3**456 4 8 4 8 1 6 2

*H. sapiens tRNA-Ala*

5’AA...**U**.5’D.**A**...**GG**.A3’D..5’AC..**U**.AC...3’AC......5’T.TP......3’T...3’AA.

**8** **1**1 2 2 **3**333 4 4 5 5 6 6 7

**4**5 1 6 **3**456 4 8 4 8 1 6 2

GGGGGUA**U**AGCUC**A**GU.**GG**UAGAGCGCGUGCU**U**AGCAUGCACGAGGUCCUGGGU**UC**GAUCCCCAGUACCUCCA Sc: 84.9  GGGGGUG**U**AGCUC**A**GU.**GG**UAGAGCGCGUGCU**U**AGCAUGCACGAGGcCCCGGGU**UC**AAUCCCCGGCACCUCCA (3) Sc: 84.7  GGGGGUG**U**AGCUC**A**GU.**GG**UAGAGCGCGUGCU**U**AGCAUGCACGAGGcCCUGGGU**UC**AAUCCCCAGCACCUCCA (2) Sc: 84.2  GGGGGUG**U**AGCUC**A**GU.**GG**UAGAGCGCGUGCU**U**AGCAUGUACGAGGUCCCGGGU**UC**AAUCCCCGGCACCUCCA Sc: 80.3  GGGGAUG**U**AGCUC**A**GU.**GG**UAGAGCGCAUGCU**U**AGCAUGCAUGAGGUCCCGGGU**UC**GAUCCCCAGCAUCUCCA Sc: 76.1  GGGGGUA**U**AGCUC**A**GC.**GG**UAGAGCGCGUGCU**U**AGCAUGCACGAGGUCCUGGGU**UC**AAUCCCCAAUACCUCCA Sc: 74.1  GGGGAAU**U**AGCUC**AA**GU**GG**UAGAGCGCUUGCU**U**AGCACGCAAGAGGU**A**GUGGG**AUC**G**A**UGCCCACAUUCUCCA (5) Sc: 60.1  GGGGGAU**U**AGCUC**AA**AU**GG**UAGAGCGCUCGCU**U**AGCAUGCGAGAGGU**A**GCGGG**AUC**G**A**UGCCCGCAUCCUCCA (2) Sc: 59.7  GGGGAAU**U**AGCUC**AA**AU**GG**UAGAGCGCUCGCU**U**AGCAUGCGAGAGGU**A**GCGGG**AUC**G**A**UGCCCGCAUUCUCCA (3) Sc: 58.9 GGGGAAU**U**AGCUC**AA**GC**GG**UAGAGCGCUUGCU**U**AGCAUGCAAGAGGU**A**GUGGG**AUC**G**A**UGCCCACAUUCUCCA (3) Sc: 58.7  GGGGAAU**U**AGCUC**AG**GC**GG**UAGAGCGCUCGCU**U**AGCAUGCGAGAGGU**A**GCGGG**AUC**G**A**CGCCCGCAUUCUCCA (2) Sc: 58.3  GGGGAAU**U**AGCUC**AA**GC**GG**UAGAGCGCUUGCU**U**AGCAUGCAAGAGGU**A**GCAGG**AUC**G**A**UGCCUGCAUUCUCCA Sc: 57.2  GGGGAAU**U**AGCUC**AA**GU**GG**UAGAGCGCUCGCU**U**AGCAUGCGAGAGGU**A**GUGGG**AUC**G**A**UGCCCGCAUUCUCCA Sc: 56.7  GGGGAAU**U**AGCCC**AA**GU**GG**UAGAGCGCUUGCU**U**AGCAUGCAAGAGGU**A**GUGGG**AUC**G**A**UGCCCACAUUCUCCA Sc: 53.1

GGGGAUG**U**AGCUC**A**GU.**GG**UAGAGCGCAUGCU**U**CGCAUGUAUGAGGUCCCGGGU**UC**GAUCCCCGGCAUCUCCA (2) Sc: 79.7  GGGGGUG**U**AGCUC**A**GU.**GG**UAGAGCGCGUGCU**U**CGCAUGUACGAGGcCCCGGGU**UC**GACCCCCGGCUCCUCCA Sc: 76.1  GGGGAUG**U**AGCUC**A**GU.**GG**UAGAGCGCGCGCU**U**CGCAUGUGUGAGGUCCCGGGU**UC**AAUCCCCGGCAUCUCCA Sc: 74.4

GGGGGUG**U**AGCUC**A**GU.**GG**UAGAGCGCAUGCU**U**UGCAUGUAUGAGGUCCCGGGU**UC**GAUCCCCGGCACCUCCA Sc: 80.5  GGGGAUG**U**AGCUC**A**GU.**GG**UAGAGCGCAUGCU**U**UGCAUGUAUGAGGUCCCGGGU**UC**GAUCCCCGGCAUCUCCA (3) Sc: 79.7  GGGGAUG**U**AGCUC**A**GU.**GG**UAGAGCGCAUGCU**U**UGCACGUAUGAGGcCCCGGGU**UC**AAUCCCCGGCAUCUCCA Sc: 78.8  GGGGGUG**U**AGCUC**A**GU.**GG**UAGAGCGCAUGCU**U**UGCAUGUAUGAGGcCUCGGGU**UC**GAUCCCCGACACCUCCA Sc: 75.3  GGGGGUG**U**AGCUC**A**GU.**GG**UAGAGCACAUGCU**U**UGCAUGUGUGAGGcCCCGGGU**UC**GAUCCCCGGCACCUCCA Sc: 74.2  GGGGGUG**U**AGCUC**A**GU.**GG**UAGAGCGCAUGCU**U**UGCAUGUAUGAGG.CCUCGGU**UC**GAUCCCCGACACCUCCA Sc: 60.0

**

*H. sapiens tRNA-Gly*

5’AA...**U**.5’D.**A**..**GG**..A3’D..5’AC..**U**.AC...3’AC.....5’T.**TPC**.....3’T...3’AA.

**8** **1**1 2 2 **3**333 4 4 **5**  5 6 6 7

**4**5 1 6 **3**456 4 8 **4**  8 1 6 2

>>>>>>>**.**.>>>>**.**..**..**...<<<<.>>>>>.**.**.....<<<<<....>>>>>**...**....<<<<<<<<<<<<. GCAUUGGUGGUUCAGUGGU.AGAAUUCUCGCCUGCCACGCGGGAGG**C**CCGGG**U**UCG**A**UUCCCGGCCAAUGCA (6) Sc: 81.0

GCAUGGGUGGUUCAGUGGU.AGAAUUCUCGCCUGCCACGCGGGAGG**C**CCGGG**U**UCG**A**UUCCCGGCCCAUGCA (5) Sc: 80.9 GCAUUGGUGGUUCAGUGGU.AGAAUUCUCGCCUGCCACGCGGGAGG**C**CCGGG**U**UUG**A**UUCCCGGCCAGUGCA Sc: 75.0 GCAUAGGUGGUUCAGUGGU.AGAAUUCUUGCCUGCCACGCAGGAGG**C**CCAGG**U**UUG**A**UUCCUGGCCCAUGCA Sc: 69.4 GCAUUGGUGGUUCAGUGGU.AGAAUUCUCGCCUGCCAUGCGGGCGG**C**CGGGC**U**UCG**A**UUCCUGGCCAAUGCA Sc: 54.7

GCAUUGGUGGUUCAGUGGU.AGAAUUCUCGCCUCCCACGCGGGAGA**C**CCGGG**U**UCA**A**UUCCCGGCCAAUGCA (2) Sc: 78.3 GCGCCGCUGGUGUAGUGGU.AUCAUGCAAGAUUCCCAUUCUUGCGA**C**CCGGG**U**UCG**A**UUCCCGGGCGGCGCA (2) Sc: 75.1

GCAUUGGUGGUUCAAUGGU.AGAAUUCUCGCCUCCCACGCAGGAGA**C**CCAGG**U**UCG**A**UUCCUGGCCAAUGCA Sc: 68.3

GCGUUGGUGGUAUAGUGGUUAGCAUA**G**CUGCCUUCCAAGCAG**U**UGA**C**CCGGG**U**UCG**A**UUCCCGGCCAACGCA (7) Sc: 73.8 GCGUUGGUGGUAUAGUGGUaAGCAUA**G**CUGCCUUCCAAGCAG**U**UGA**C**CCGGG**U**UCG**A**UUCCCGGCCAACGCA Sc: 73.8 GCGUUGGUGGUAUAGUGGUgAGCAUA**G**UUGCCUUCCAAGCAG**U**UGA**C**CCGGG**C**UCG**A**UUCCCGCCCAACGCA Sc: 56.0

*B. mori Gly tRNAs*

5’AA...**U**.5’D.**A**...**GG**..A3’D..5’AC..**U**.AC...3’AC.....5’T.**TPC**.....3’T...3’AA.

**8** **1**1 2 2 **3**333 4 4 **5**  5 6 6 7

**4**5 1 6 **3**456 4 8 **4**  8 1 6 2

>>>>>>>**.**.>>>>**.**........<<<<.>>>>>.......<<<<<....>>>>>**...**....<<<<<<<<<<<<. GCAUCGG**U**GGUUC**A**GU.**GG**U.AGAAUGCUCGCCUGCCACGCGGGCGGCCCGGG**UUC**GAUUCCCGGCCGAUGCA (27) Sc: 80.1 GCAACGG**U**AGUUC**A**GU.**GG**U.AGAACACUCGCUUGCCAAGCGGGUAGCUCGGG**UUC**GAUUCCCGGCCGUUGCA Sc: 71.4  GCAUCGG**U**GGUUC**A**GU.**GG**U.AGAAUACUCGCUUGCCAAGCGGGCGACUCGGG**UUC**GAUUCCCGGCCGAUGCA Sc: 70.7

GCAUCGG**U**GGUUC**A**GU.**GG**U.AGAAUGCUCGCCUGCCACGCGGGCAGCCCGGG**UUC**GAUUCCCGGCCAAUGCA Sc: 68.1

GCACUGC**U**GGUG**UA**GU.**GG**U.A**U**CAUGCAAGAUUCCCAUUCUUGCGACCCGGG**UUC**GAUUCCCGGGCAGUGCA (3) Sc: 75.2

GCGUUGG**U**GGUGU**A**AU.**GG**UcAGCAUAGUUGCCUUCCAAGCAAUUGAUCCGGG**UUC**GAUUCCCGGCCAACGCA Sc: 74.2  GCGUUGG**U**GGUGU**A**AU.**GG**UcAGCAUAGUUGCCUUCCAAGCAGUUGAUCCGGG**UUC**GAUUCCCGGCCAACGCA (10) Sc: 70.4  GCGAUGG**U**GGUGU**A**AU.**GG**UcAGCAUAGUUGCCUUCCAAGCAGUUGAUCCGGG**UUC**GAUUCCCGGCCAUCGCA (2) Sc: 69.6  GCGAUGG**U**GGUGU**A**AU.**GG**UcAGCAUAGUUGCCUUCCAAGCAGUUGAUCCGGG**UUC**GAUUCCCGGCCAUCGCA Sc: 69.6  GCGUUGG**U**GGUGU**A**AU.**GG**UcAGCAUAGUUGCCUUCCAAGCAGUUGAUCCGGG**UUC**GAUUCCCGGCCUACGCA Sc: 65.7

Pro tRNAs

>>>>>>>..>>>>.........<<<<.>>>>>.......<<<<<.....>>>>>.......<<<<<<<<<<<<.

5’AA...**U**.5’D.**A**..**GG.**..A3’D..5’AC..**U**.AC...3’AC......5’T.TP......3’T...3’AA.

**8** **1**1 2 2 **3**333 4 4 5 5 6 6 7

**4**5 1 6 **3**456 4 8 4 8 1 6 2

GGCUCGUUG**G**UC**U**AG.GGGU.A**U**GA**U**UCUCGCUUAGGGUGCGAGAGGUCCCGGGUUCAAAUCCCGGACGAGCCC Sc: 70.2 8

GGCUCGUUG**G**UC**U**AG.GGGU.A**U**GA**U**UCUCGCUUCGGGUGCGAGAGGUCCCGGGUUCAAAUCCCGGACGAGCCC Sc: 70.2 3

GGCUCGUUG**G**UC**U**AG.GGGU.A**U**GA**U**UCUCGCUUUGGGUGCGAGAGGUCCCGGGUUCAAAUCCCGGACGAGCCC Sc: 70.3 5

GGCUCGUUG**G**UC**U**AG.GGGU.A**U**GA**U**UCUCGCUUAGGGUGCGAGAGGUCCCGGGUUCAAAUCCCGGACGAGCCC Sc: 70.2 6

GGCUCGUUG**G**UC**U**AG.GGGU.A**U**GA**U**UCUCGCUUCGGGUGCGAGAGGUCCCGGGUUCAAAUCCCGGACGAGCCC Sc: 70.2 3

GGCUCGUUG**G**UC**U**AG.GGGU.A**U**GA**U**UCUCGCUUUGGGUGCGAGAGGUCCCGGGUUCAAAUCCCGGACGAGCCC Sc: 70.3 4

GGCUCGUUG**G**UC**U**AG.GGGU.A**U**GA**U**U**U**UCGCUUAGGGUGCGA**G**AGGUCCCGGGUUCAAAUCCCGGACGAGCCC Sc: 68.3 8

GGCUCGUUG**G**UC**U**AG.GGGU.A**U**GA**U**UCUCGCUUCGGGUGCGAGAGGUCCCGGGUUCAAAUCCCGGACGAGCCC Sc: 70.2 3

GGCUCGUUG**G**UC**U**AG.GGGU.A**U**GA**U**UCUCGCUUUGGGUGCGAGAGGUCCCGGGUUCAAAUCCCGGACGAGCCC Sc: 70.3 4

Val tRNAs

GUUUCCGUAGUG**U**AGUGGUU.A**U**CACGUUCGCCUAACACGCGAAAGGUCCCCGGUUCGAAACCGGGCGGAAACA Sc: 79.4 6

GUUUCCGUAGUG**U**AGUGGUU.A**U**CACGUUCGCCUCACACGCGAAAGGUCCCCGGUUCGAAACCGGGCGGAAACA Sc: 79.4 8

GGUUCCAUAGUG**U**AGUGGUU.A**U**CACGUCUGCUUUACACGCAGAAGGUCCUGGGUUCGAGCCCCAGUGGAACCA Sc: 82.9 2

GUUUCCGUAGUG**U**AGUGGUU.A**U**CACGUUCGCCUAACACGCGAAAGGUCCCUGG**A**UCA**A**AACCAGGCGGAAACA Sc: 72.8 ?

GUUUCCGUAGUG**U**AGUGGUU.A**U**CACGUUCGCCUAACACGCGAAAGGUCCCCGGUUCGAAACCGGGCGGAAACA Sc: 79.4 4

GUUUCCGUAGUG**U**AGUGGUU.A**U**CACGUUCGCCUCACACGCGAAAGGUCCCCGGUUCGAAACCGGGCGGAAACA Sc: 79.4 7

GGUUCCAUAGUG**U**AGCGGUU.A**U**CACGUCUGCUUUACACGCAGAAGGUCCUGGGUUCGAGCCCCAGUGGAACCA Sc: 80.5 3

GGUG**A**UAUAGCU**C**AGUGGUg.A**G**AGCAUUUGCCUAACAUGCAAAA.GUCCUGGGUUCAAUUCCCAGUA**C**CAUAG Sc: 68.9 ?

GUUUCCGUGGUG**U**AGCGGUU.A**U**CACAUCUGCCUAACACGCAGAAGGUCCCCGGUUCGAUCCCGGGCGGAAACA Sc: 79.5 7

GUUUCCGUGGUG**U**AGUGGUU.A**U**CACAUCUGCCUGACACGCAGAAGGUCCUCGGUUCGAUCCCGAGCGGAAACA Sc: 81.5

GUUUCCGUAGUG**U**AGCGGUU.A**U**CACGUGUGCUUCACACGCACAAGGUCCCCGGUUCGAUCCCGGGCGGAAACA Sc: 77.7 4

GGUUCCGUGGUG**U**AGUGGUU.A**U**CACAUCUGCUUUACACGCAGAAGGcCGCCAGUUCGAUCCUGGCCGGAAUCA Sc: 75.8 6

Thr tRNAs

>>>>>>>..>>>>.........<<<<.>>>>>.......<<<<<.....>>>>>.......<<<<<<<<<<<<.

5’AA...**U**.5’D.**A**..**.GG**..A3’D..5’AC..**U**.AC...3’AC......5’T.TP......3’T...3’AA.

**8** **1**1 2 2 **3**333 4 4 5 5 6 6 7

**4**5 1 6 **3**456 4 8 4 8 1 6 2

GGC**G**CCGUGGC**U**UAGUUGGUUAA**A**GCGCCUGUCUAGUAAACAGGAGAUCCUGGGUUCGAAUCCCAGCGG**U**GCCU Sc: 82.6 3

GGC**U**CCGUGGC**U**UAGCUGGUUAA**A**GCGCCUGUCUAGUAAACAGGAGAUCCUGGGUUCGAAUCCCAGCGG**G**GCCU Sc: 81.4 2

GGC**G**CGGUGGC**CA**AGU.GGU.A**AG**GCGUCGGUCUCGUAAACCGAAGAUCACGGGUUCGAACCCCGUCCG**U**GCCU Sc: 79.6

GGC**U**CUAUGGC**U**UAGUUGGUUAA**A**GCGCCUGUCUCGUAAACAGGAGAUCCUGGGUUCGACUCCCAGUGG**G**GCCU Sc: 78.4

GGC**U**CCAU**A**GC**U**CAGU.GGUUAG**A**GCACUGGUCUUGUAAACCAGGG**G**UCGCGAGUUCGAUCCUCGCUGG**G**GCCU Sc: 84.1

GGCUCUAUGGC**U**UAGUUGGUUAA**A**GCGCCUGUCUUGUAAACAGGAGAUCCUGGGUUCGAAUCCCAGUAGAGCCU Sc: 83.3

GGC**G**CCGUGGCUUAGUUGGUUAAAGCGCCUGUCUAGUAAACAGGAGAUCCUGGGUUCGAAUCCCAGCGG**U**GCCU Sc: 82.6 3

GGC**U**CCGUGGCUUAGCUGGUUAAAGCGCCUGUCUAGUAAACAGGAGAUCCUGGGUUCGAAUCCCAGCGG**G**GCCU Sc: 81.4

GGC**G**CGGUGGCC**A**AGU.GGU.A**A**GGCGUCGGUCUCGUAAACCGAAGAUCACGGGUUCGAACCCCGUCCG**U**GCCU Sc: 79.6

GGC**U**CCAUGGCUUAGCUGGUUAAAGCGCCUGUCUCGUAAACAGGAGAUCCUGGGUUCGACUCCCAGUGG**G**GCCU Sc: 78.2

GGC**U**CCAUGGCUUAGUUGGUUAAAGCGCCUGUCUUGUAAACAGGAGAUCCUGGGUUCGAAUCCCAGUGG**G**GCCU Sc: 83.6

GGC**U**CCAUAGCUCAGG.GGUUAGAGCACUGGUCUUGUAAACCAGGGGUCGCGAGUUCAAAUCUCGCUGG**G**GCCU Sc: 78.8 2

GGC**G**CCGUGGCUUAGUUGGUUAAAGCGCCUGUCUAGUAAACAGGAGAUCGGGGGUUCGAAUCCCCCCGG**G**GCCU Sc: 76.4 4

GCCUC**U**UUAGCUCAGU.GGU.AGAGCACUGGUCUCGUAAACCAGGGGUCGUGAGUUCAAUCCUCACA**G**GAGGCA Sc: 82.7 3

GCC**U**CCAUAGCUCAGG.GGUUAGAGCACUGGUCUUGUAAACCAGGGGUCGAGAGUUCAAAUCUCUCUGG**G**GGCA Sc: 78.9 4

Arg tRNAs

G**G**GCCAGUGGCG**C**AAU.GGAU.A**A**CGCGUCUGACUACGGAUCAGAAGAUUC**U**AGGUUCGACUCCU**G**GCUGGC**U**CG Sc: 64.2 4

G**G**CCGCGUGGCCUAAU.GGAU.AAGGCGUCUGAUUCCGGAUCAGAAGAUU**G**AGGGUUCGAGUCCCU**U**CGUGG**U**CG Sc: 67.0 3

**G**ACCACGUGGCCUAAU.GGAU.AAGGCGUCUGACUUCGGAUCAGAAGAUU**G**AGGGUUCGAAUCCCU**U**CGUGGU**U**A Sc: 66.8

**G**CCCCAGUGGCCUAAU.GGAU.AAGGCACUGGCCUCCUAAGCCAGGGAUUGUGGGUUCGAGUCCCACCUGGGG**U**A Sc: 72.3

**G**UCUCUGUGGCGCAAU.GGAcgAGCGCGCUGGACUUCUAAUCCAGAGGUUCCGGGUUCGAGUCCCGGCAGAGA**U**G Sc: 78.5

G**G**CUCCGUGGCGCAAU.GGAU.AGCGCAUUG**G**ACUUCUA.Intron.AU**U**CAAAGGUUCCGGGUUCGAGUCCCGGCGGAG**U**CG Sc: 71.1

G**G**GCCAGUGGCG**C**AAU.GGAU.A**A**CGCGUCUGACUACGGAUCAGAAGAUUCCAGGUUCGACUCCUGGCUGGC**U**CG Sc: 67.6 (3)

GACCCAGUGGCCUAAU.GGAU.AAGGCA**U**CAGCCUCCGGAGCUG**G**GGAUU**G**UGGGUUCGAGUCCCA**U**CUGGGUCG Sc: 64.9

**G**ACCACGUGGCCUAAU.GGAU.AAGGCGUCUGACUUCGGAUCAGAAGAUU**G**AGGGUUCGAAUCCCU**U**CGUGGU**U**G Sc: 65.5 (2)

**G**CCCCAGUGGCCUAAU.GGAU.AAGGCACUGGCCUCCUAAGCCAGGGAUUGUGGGUUCGAGUCCCACCUGGGG**U**G Sc: 71.1 (2)

**G**UCUCUGUGGCGCAAU.GGAcgAGCGCGCUGGACUUCUAAUCCAGAGGUUC**U**GGGUUCGAGUCCC**G**GCAGAGA**U**G Sc: 75.1

G**G**CUCCGUGGCGCAAU.GGAU.AGCGCAUUG**G**ACUUCUA.Intron.AU**U**CAAAGGUUCCGGGUUCGAGUCCCGGCGGAG**U**CG Sc: 71.1

G**G**UCCUGUGGCG**C**AAU.GGAU.A**A**CGCGUCUGACUACGGAUCAGAAGAUUCCAGGUUCGAGUCCUGGCAGGA**U**CG Sc: 71.4 5

GACCGUGUGGCCUAAU.GGAU.AAGGCGUCGGACUUCGGAUCCGAAGAUU**G**CAGGUUCGAGUCCUG**U**CACGGUCG Sc: 70.8 7

**G**CCCCUGUGGCCUAAU.GGAU.AAGGCA**U**CGGCCUCCUAAGCCG**G**GGAUUGUGGGUUCGAGUCCCACCAGGGG**U**A Sc: 70.3 5

**G**UCCCUGUGGCGCAGA.GGAU.AGCGCGUUGGACUUCUAAUCCAAAGGUCGUGGGUUCGAUCCCCACCAGGGA**U**G Sc: 81.2 3

Leu tRNAs

>>>>>>>..>>>>..........<<<<.>>>>>...............<<<<<...............>>>>>.......<<<<<<<<<<<<.

5’AA...**U**.5’D.**A**..**.GG**...A3’D..5’AC..**U**.AC...........3’AC................5’T.TP......3’T...3’AA.

**8** **1**1 2 2 **3**333 4 4 5 5 6 6 7

**4**5 1 6 **3**456 4 8 4 8 1 6 2

GG**U**AGCGUGGCC**G**AGC.GGUcUA**A**GGCGCUGGAUUAAGG. ......CUCCAGU*CUCU*UC..*GGGG*GCGUGGGUUCGAAUCCCACCGCU**G**CCA 74.8 4

GUCAGGAUGGCC**G**AGC.GGUcUA**A**GGCGCUGCGUUCAGG. ......UCGCAGU*CUCC*CCU.*GGAG*GCGUGGGUUCGAAUCCCACUCCUGACA 78.2 7

GG**U**AGCGUGGCC**G**AGC.GGUcUA**A**GGCGCUGGAUUUAGG. ......CUCCAGU*CUCU*UC..*GGAG*GCGUGGGUUCGAAUCCCACCGCU**G**CCA 75.2

GUCAG**G**AUGGCC**G**AGU.GGUcUA**A**GGCGCCAGACUCAAG.Intron.UUCUGGU*CUCC*GUAU*GGAG*GCGUGGGUUCGAAUCCCACU**U**CUGACA 78.0

ACCG**G**GAUGGCC**G**AGU.GGU.UA**A**GGCGUUGGACUUAAG........AUCCAAU*GGGC*UGGU*GCCC*GCGUGGGUUCGAACCCCACUC**U**CGGUA 90.8

GG**U**AGCGUGGCC**G**AGC.GGUcUA**A**GGCGCUGGAUUAAGG........CUCCAGU*CUCU*UC..*GGGG*GCGUGGGUUCGAAUCCCACCGCU**G**CCA 74.8 3

GUCAGGAUGGCC**G**AGC.GGUcUA**A**GGCGCUGCGUUCAGG........UCGCAGU*CUCC*CCU.*GGAG*GCGUGGGUUCGAAUCCCACUCCUGACA 78.2 5

GG**U**AGCGUGGCC**G**AGC.GGUcUA**A**GGCGCUGGAUUUAGG........CUCCAGU*CUCU*UC..*GGAG*GCGUGGGUUCGAAUCCCACCGCU**G**CCA 75.2

GUCAG**G**AUGGCC**G**AGU.GGUcUA**A**GGCGCCAGACUCAAG.Intron.UUCUGGU*CUCC*GUAU*GGAG*GCGUGGGUUCGAAUCCCACU**U**CUGACA 78.1

ACCAGAAUGGCC**G**AGU.GGU.UA**A**GGCGUUGGACUUAAG........AUCCAAU*GGAU*UUAU*AUCC*GCGUGGGUUCGAACCCCACUUCUGGUA 86.4

GG**U**AGCGUGGCC**G**AGC.GGUcUA**A**GGCGCUGGUUUAAGG........CACCAGU*CUCU*UC..*GGAG*GCGUGGGUUCGAAUCCCACCGCU**G**CCA 71.6 6

GUCAG**G**AUGGCC**G**AGC.GGUcUA**A**GGCGCUGCGUUCAGG........UCGCAGU*CCAC*UUCU*GUGG*GCGUGGGUUCGAAUCCCACU**U**CUGACA 74.6 6

G**G**CAGCGUGGCC**G**AGU.GGUcUA**A**GGCGCUGGUUUUAGG........CACCAGU*CCGA*....*AAGG*GCGUGGGUUCGAAUCCCACCGCUG**U**CA 71.6 2

GUCAG**G**AUGGCC**G**AGC.GGUcUA**A**GGCGCCAGACUCAAG.Intron.UUCUGGU*CCUC*UCU.*GAGG*GCGUGGGUUCGAAUCCCACU**U**CUGACA 74.2 4

GUCA**G**GUUGGCC**G**AGU.GGUcUA**A**GGCGCCAGAUUUAAG........CUCUGGU*UCCC*GAGA*GGGA*GCGUGGGUUCGAACCCCACACC**U**GACA 73.6 4

Ser tRNAs

>>>>>>>..>>>>.........<<<<.>>>>>.......<<<<<..............>>>>>.......<<<<<<<<<<<<.

5’AA...**U**.5’D.**A**..**.GG**..A3’D..5’AC..**U**.AC...3’AC...............5’T.TP......3’T...3’AA.

**8** **1**1 2 2 **3**333 4 4 5 5 6 6 7

**4**5 1 6 **3**456 4 8 4 8 1 6 2

GUAGUCGUGGCC**G**AGU.GGUUA**A**GGCGAUGGACUAGAAAUCCAUU*GGGG*UCU*CCCC*GCGCAGGUUCGAAUCCUGCCGACUACG 89.6 6

GCUGUGAUGGCC**G**AGU.GGUUA**A**GGCGUUGGACUCGAAAUCCAAU*GGGG*UCU*CCCC*GCGCAGGUUCGAAUCCUGCUCACAGCG 95.1

GCAGCGAUGGCC**G**AGU.GGUUA**A**GGCGUUGGACUUGAAAUCCAAU*GGGG*UCU*CCCC*GCGCAGGUUCGAACCCUGCUCGCUGCG 94.0

GACGAGGUGGCC**G**AGU.GGUUA**A**GGCGAUGGACUGCUAAUCCAUU*GUGC*UCU*GCAC*GC**G**UGGGUUCGAAUCCCA**U**CCUCGUCG 88.4 3

GUAGUCGUGGCC**G**AGU.GGUUA**A**GGCGAUGGACUAGAAAUCCAUU*GGGG*UCU*CCCC*GCGCAGGUUCGAAUCCUGCCGACUACG 89.6 6

GCUGUGAUGGCC**G**AGU.GGUUA**A**GGCGUUGGACUCGAAAUCCAAU*GGGG*UCU*CCCC*GCGCAGGUUCGAAUCCUGCUCACAGCG 95.1

GUAGUCGUGGCC**G**AGU.GGUUA**A**GGCGAUGGACUUGAAAUCCAUU*GGGG*UUU*CCCC*GCGCAGGUUCGAAUCCUGCCGACUACG 90.4 2

GACGAGGUGGCC**G**AGU.GGUUA**A**GGCGAUGGACUGCUAAUCCAUU*GUGC*UCU*GCAC*GC**G**UGGGUUCGAAUCCCA**U**CCUCGUCG 88.4 3

GCAGUCGUGGCC**G**AGC.GGUUA**A**GGCGUCUGACUAGAAAUCAGAU*UCCC*UCU*GGGA*GCGUAGGUUCGAAUCCUACCGACUGCG 85.0 8

GCAG**U**CGUGGCC**G**AGU.GGUUA**A**GGCGUCUGACUCGAAAUCAGAU*UCCC*UCU*GGGA*GCGUAGGUUCGAAUCCUACCG**G**CUGCG 84.6 3

GCUGAGGUGUCC**G**AGU.GGUUA**A**GGAGUUGGACUUGAAAUCCAAU*GGGU*UAU*ACCC*GC**G**CAGGUUCGAAUCCUG**U**CCUCAGCG 82.6 5

GACGAGGUGGCC**G**AGU.GGUUA**A**GGCGUUGGACUGCUAAUCCAAU*GUGC*UCU*GCAC*GC**G**UGGGUUCGAAUCCCA**U**CCUCGUCG 90.9 7

Glu tRNAs

>>>>>>>..>>>>.........<<<<.>>>>>.......<<<<<....>>>>>.......<<<<<<<<<<<<.

5’AA...**U**.5’D.**A**..**.GG**..A3’D..5’AC..**U**.AC...3’AC.....5’T.TP......3’T...3’AA.

**8** **1**1 2 2 **3**333 4 4 5 5 6 6 7

**4**5 1 6 **3**456 4 8 4 8 1 6 2

UCCCUG**G**UG**G**UC**U**AGU.GGUUA**G**GA**U**UCGGCGCUCUCACCGCCGCGGCCCGGGUUCGAUUCCCGG**U**CAGGGAA 73.2 7

UCCCAUAUG**G**UC**U**AGC.GGUUA**G**GA**U**UCCUGGUUUUCACCCAGGUGGCCCGGGUUCGACUCCCGGUAUGGGAA 72.5 2

UCCCUG**G**UG**G**UC**U**AGU.GGUUA**G**GA**U**UCGGCGCUCUCACCGCCGCGGCCCGGGUUCGAUUCCCGG**U**CAGGGAA 73.2 9

UCCCACAUG**G**UC**U**AGC.GGUUA**G**GA**U**UCCUGGUUUUCACCCAGGCGGCCCGGGUUCGACUCCCGGUGUGGGAA 71.2 4

UCCGAUAUG**G**UC**U**AGU.GGCUA**G**GA**U**ACCUGGCUCUCACCCAGGAGGC**U**CGGGUUCGAUUCCCG**G**UAUCGGAA 69.8 6

UCCCGUAUG**G**UC**U**AGU.GGCUA**G**GA**U**ACCUGGCUUUCACCCAGGAGGC**U**CGGGUUCGAUUCCCG**G**UACGGGAA 70.9 12

Gln tRNAs

GGUUCCAUGGUG**U**AAU.GGUUA**G**CACUCUGGACUCUGAAUCCAGCGAUCCGAGUUCAAAUCUCGGUGGAACCU 71.5 5

GG**C**CCCAUGGUG**U**AAU.GGUUA**G**CACUCUGGACUUUGAAUCCAGCGAUCCGAGUUCAAAUCUCGGUGGG**A**CCU 66.9 3

GGU**U**CCAUGGUG**U**AAU.GGUUA**G**CACUCUGGACUCUGAAUCCAGCGAUCCGAGUUCAAAUCUCGGUGG**G**ACCU 71.1 4

GG**C**CCCAUGGUG**U**AAU.GGUUA**G**CACUCUGGACUUUGAAUCCAGCGAUCCGAGUUCAAAUCUCGGUGGG**A**CCU 66.9 2

GGUUCCAUGGUG**U**AAU.GGUUA**G**CACUCUGGACUCUGAAUCCAGCGAUCCGAGUUCAAAUCUCGGUGGAACCU 71.5 6

GGUUCCAUGGUG**U**AAU.GGUUA**G**CACUCUGGACUUUGAAUCCAGCGAUCCGAGUUCAAAUCUCGGUGGAACCU 71.5 5

Asp tRNAs

UCC**U**CGUUAGUA**U**AGU.GGUgA**G**UAUCCCCGCCUGUCACGCGGGAGACCGGGGUUCGAUUCCCCGACG**G**GGAG Sc: 66.5 11

UCC**U**CGUUA**G**UA**U**AGU.GGUgA**G**UA**U**CCCCGCCUGUCACGCGGGAGACCGGGGUUCGAUUCCCCGACG**G**GGAG Sc: 66.5 13

UCU**U**CGGUA**G**UA**U**AGU.GGUcA**G**UA**U**CCCCGCCUGUCACGCGGGAGACCGGGGUUCGAUUCCCCGCCG**G**AGAG Sc: 66.3 13

His tRNAs

GCCGUGAUCGUA**U**AGU.GGUUA**G**UACUCUGCGUUGUGGCCGCAGCAACCUCGGUUCGAAUCCGAGUCACGGCA 60.1 9

GCCGUGAUCGUA**U**AGU.GGUUA**G**UACUCUGCGUUGUGGCCGCAGCAACCUCGGUUCGAAUCCGAGUCACGGCA 60.1 8

GCCGUGAUCGUC**U**AGU.GGUUA**G**GACCCUACGUUGUGGCCGUAGUAACCCAGGUUCGAAUCCUGGUCACGGCA 63.5 14

Ile tRNAs

GCUCCAGUGGCGCAAUCGGUUAGCGCGCGG**U**ACUUAUA.Intron.AU**G**CCGAGGUUGUGAGUUCGAUCCUCACCUGGAGCA 73.8 5

GCUCCAGUGGCGCAAUCGGUUAGCGCGCGG**U**ACUUAUA.Intron.AU**G**CCGAGGUUGUGAGUUCGAUCCUCACCUGGAGCA 73.7 4

GCUCCAGUGGCGCAAUUGGUUAGCGCACGG**U**ACUUAUA.Intron.AU**G**CCGGGGUUGUGAGUUCGAGCCUCACCUGGAGCA 70.4 6

GGCC**G**GUUAGCUCAGUUGGUUAGAGCGUGG**U**GCUAAUAAC**G**CCAAGGUC**G**CGGGUUCGAACCCCG**U**AC**G**GGCCA 81.2 8

GGCC**G**GUUAGCUCAGUUGGUUAGAGCGUGG**U**GCUAAUAAC**G**CCAAGGUC**G**CGGGUUCGAUCCCCG**U**AC**G**GGCCA 81.2 8

GGCCCAUUAGCUCAGUUGGUUAGAGCGUCG**U**GCUAAUAAC**G**CGAAGGUCG**C**GGGUUCGAUCCCC**U**CAUGGGCCA 81.7 10

H.s.

GGCCGGUUAGCUCAGUUGGUaAGAGCGUGGUGCUGAUAACACCAAGGUCGCGGG**C**UCG**A**CUCCCGCACCGGCCA 75.5 6

Lys tRNAs

GCCC**GG**CUAGCUCAGUCGGU.AGAGCAUGAGACUCUUAAUCUCAGGGUCGUGGGUUCGAGCCCCACG**UU**GGGCG 87.8 5

GCCC**GG**CUAGCUCAGUCGGU.AGAGCAUGAGACUCUUAAUCUCAGGGUCGUGGGUUCGAGCCCCACG**UU**GGGCG 87.8 7

GCCC**GG**CUAGCUCAGUCGGU.AGAGCAUGAGACUCUUAAUCUCAGGGUCGUGGGUUCGAGCCCCACG**UU**GGGCG 87.8 10

GCCCG**G**AUAGCUCAGUCGGU.AGAGCAUCAGACUUUUAAUCUGAGGGUCCAGGGUUCAAGUCCCUGU**U**CGGGCG 87.8 5

GCCCG**G**AUAGCUCAGUCGGU.AGAGCAUCAGACUUUUAAUCUGAGGGUCCAGGGUUCAAGUCCCUGU**U**CGGGCG 87.8 6

GCCCG**G**AUAGCUCAGUCGGU.AGAGCAUUGGACUUUUAAUCCAAGGGUCCAGGGUUCAAGUCCCUGU**U**CGGGCG 88.5 7

Cys tRNAs

GGGGG**U**AUAGCUCAGU.GGU.AGAGCA**U**UUGACUGCAGAUCAA**G**AGGUCCCCGGUUCAAAUCCGGGU**G**CCCCCU 81.9 5

GGGGG**U**AUAGCUCAGU.GGU.AGAGCA**U**UUGACUGCAGAUCAA**G**AGGtCCCCGGUUCAAAUCCGGGU**G**CCCCCU 81.9 4

GGGGA**U**AUAGCUCAGU.GGU.AGAGCAAUCGACUGCAGAUCGAUAGGcCCCCGGUUCAAACCCGGGU**G**UCCCCU 80.0 5

Trp tRNAs

GACC**U**CGUGGCGCAACGGU..AGCGCGUCUGACUCCAGAUCAGAAGGUU**G**CGUGUUCAAAUCACG**U**CG**G**GGUCA 73.3 3

GACC**U**CGUGGCGCAACGGU..AGCGCGUCUGACUCCAGAUCAGAAGGUU**G**CGUGUUCAAAUCACG**U**CG**G**GGUCA 73.3 2

GAC**U**CCGUGGCGCAACGGU..AGCGCGUCUGACUCCAGAUCAGAAGGUU**G**CGUGUUCAAAUCACG**U**CGG**G**GUCA 74.9 9

Asn tRNAs

GUC**U**CUGUGGCGCAAUCGGUUAGCGCGUUCGGCUGUUAACCGAAAGGUUGGUGGUUCGAGCCCACCCAG**G**GACG 80.9 8

GUC**U**CUGUGGCGCAAUCGGUUAGCGCGUUCGGCUGUUAACCGAAAGGUUGGUGGUUCGAGCCCACCCAG**G**GACG 80.9 9

GCC**U**CCGUGGCGCAAUCGGUUAGCGCGUUCGGCUGUUAACCGAAAGGUUGGUGGUUCGAGUCCACCCGG**G**GGCG 83.8 11

Phe tRNAs

GCCGAAAUAGCUCAGUUGGG.AGAGCGUUAGACUGAAGAUCUAAAGGUCCC**U**GGUUCGAUCCC**G**GGUUUCGGCA 88.9 6

GCCGAAAUAGCUCAGUUGGG.AGAGCGUUAGACUGAAGAUCUAAAGGUCCC**U**GGUUCGAUCCC**G**GGUUUCGGCA 88.9 5

GCCGAAAUAGCUCAGUUGGG.AGAGCGUUAGACUGAAGAUCUAAAGGUCCCCGGUUCAAUCCCGGGUUUCGGCA 88.0 5

Met tRNAs

GCCCUCUUAGCGCAGC.GGG.AGCGCGUCAG**U**CUCAUAA**U**CUGAAGGUCCUGAGUUCGAGCCUCAGAGAGGGCA 80.2 3 GCC**U**CCUUAGCGCAGUAGGC.AGCGCGUCAG**U**CUCAUAA**U**CUGAAGGUCCUGAGUUCGAACCUCAGAGG**G**GGCA 77.4 3

GCC**U**CCUUAGCGCAGUAGGC.AGCGCGUCAG**U**CUCAUAA**U**CUGAAGGUCCUGAGUUCGAACCUCAGAGG**G**GGCA 77.4 3

GCC**U**CGGUAGCGCAGUAGGC.AGCGCGUAAG**U**CUCAUAA**U**CUUAAGGUCGUGAGUUCGAUCCUCACCCG**G**GGCA 77.9 7

Met^i^ tRNAs

AGCAGAGUG**G**CGCAGC.GGA.AGCG**U**GCUGGGC**C**CAUAACCCAGAGGUCGAUGG**A**UCG**A**AACCAUCCUCUGCUA 60.4 8

AGCAGAGUG**G**CGCAGC.GGA.AGCG**U**GCUGGGC**C**CAUAACCCAGAGGUCGAUGG**A**UCG**A**AACCAUCCUCUGCUA 60.4 7

AGCAGAGUG**G**CGCAGU.GGA.AGCG**U**GCUGGGC**C**CAUAACCCAGAGGUCCGUGG**A**UCG**A**AACCACGCUCUGCUA 63.4 5

AGCAGAGUG**G**CGCAGU.GGA.AGCG**U**GCUGGGC**C**CAUAACCCAGAGGUCC**G**UGG**A**UCG**A**AACCA**U**GCUCUGCUA 59.6 6

Tyr tRNAs

CCU**U**CGAUAGCUCAGUUGGU.AGAGCG**G**AGGACUGUAG.Intron.AUCCU**U**AGGUCGC**U**GGUUCGAAUCC**G**GCUCG**G**AGGA 77.5 7

CCUUCGAUAGCUCAGUUGGU.AGAGCG**G**AGGACUGUAG.Intron.AUCCU**U**AGGUCGC**U**GGUUCGAAUCC**G**GCUCGAAGGA 78.0 3

CCUUCGAUAGCUCAGUUGGU.AGAGCG**G**UGGACUGUAG.Intron.AUCCA**U**AGGUCGC**U**GGUUCAAAUCC**G**GCUCGAAGGA 79.0 7

*H.s.*

CCUUCAAUAGUUCAGCUGGU.AGAGCA**G**AGGACUAUAG.Intron.GUCCU**U**AGGUUGCUGGUUCGAUUCCAGCUUGAAGGA 57.6
