# Supplementary material for: Eukaryotic tRNA sequences present conserved and amino acid-specific structural signatures
Source: Nucleic Acids Res. 2022 Apr 5;50(7):4100–12. doi: 10.1093/nar/gkac222 (PMC9023262; doi:10.1093/nar/gkac222)
Supplement: gkac222_Supplemental_Files [file gkac222_supplemental_files.zip › Sup_Data_2_2D_Struct.pptx]

## Slide 1
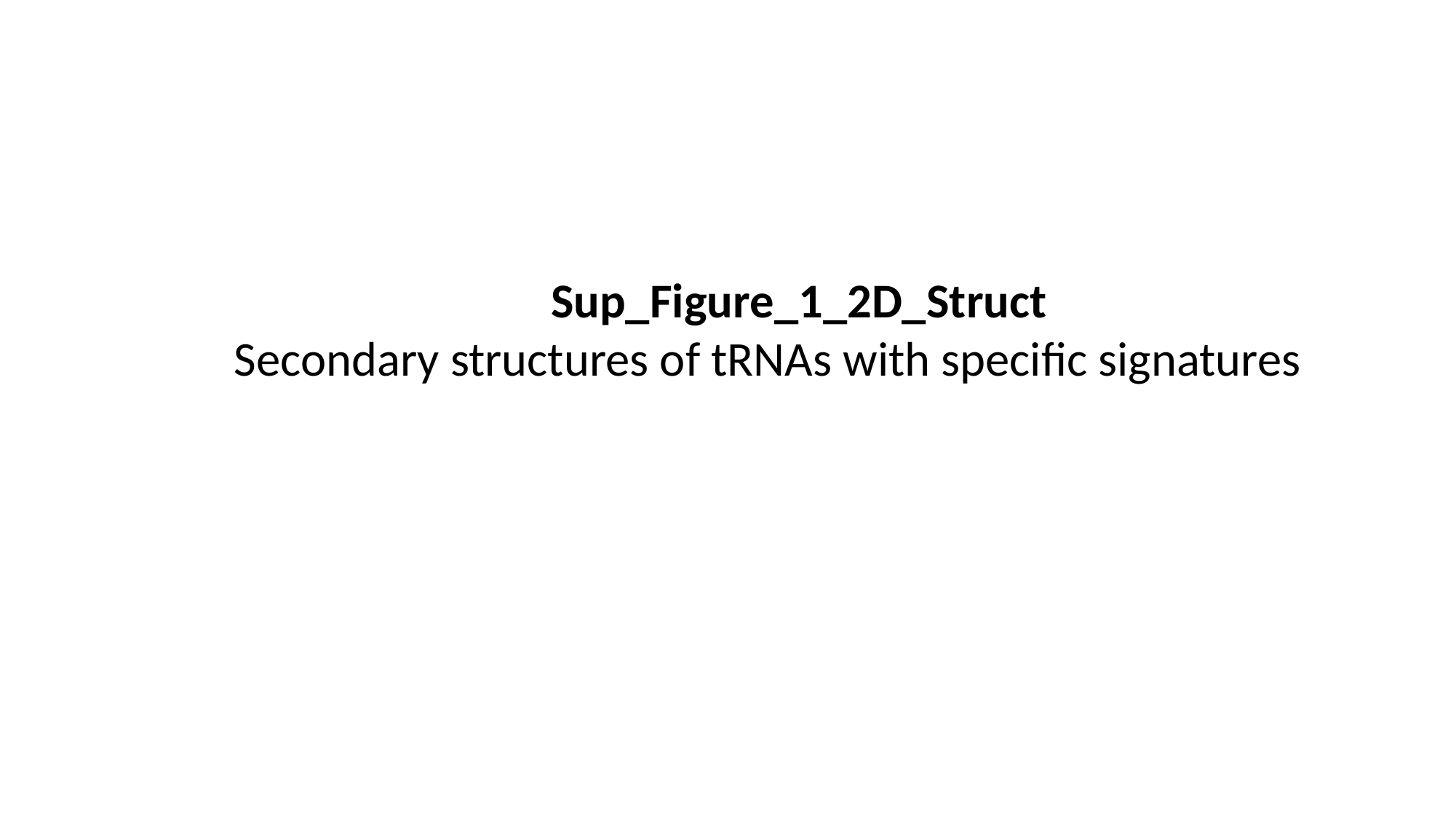

Sup_Figure_1_2D_Struct
Secondary structures of tRNAs with specific signatures

## Slide 2
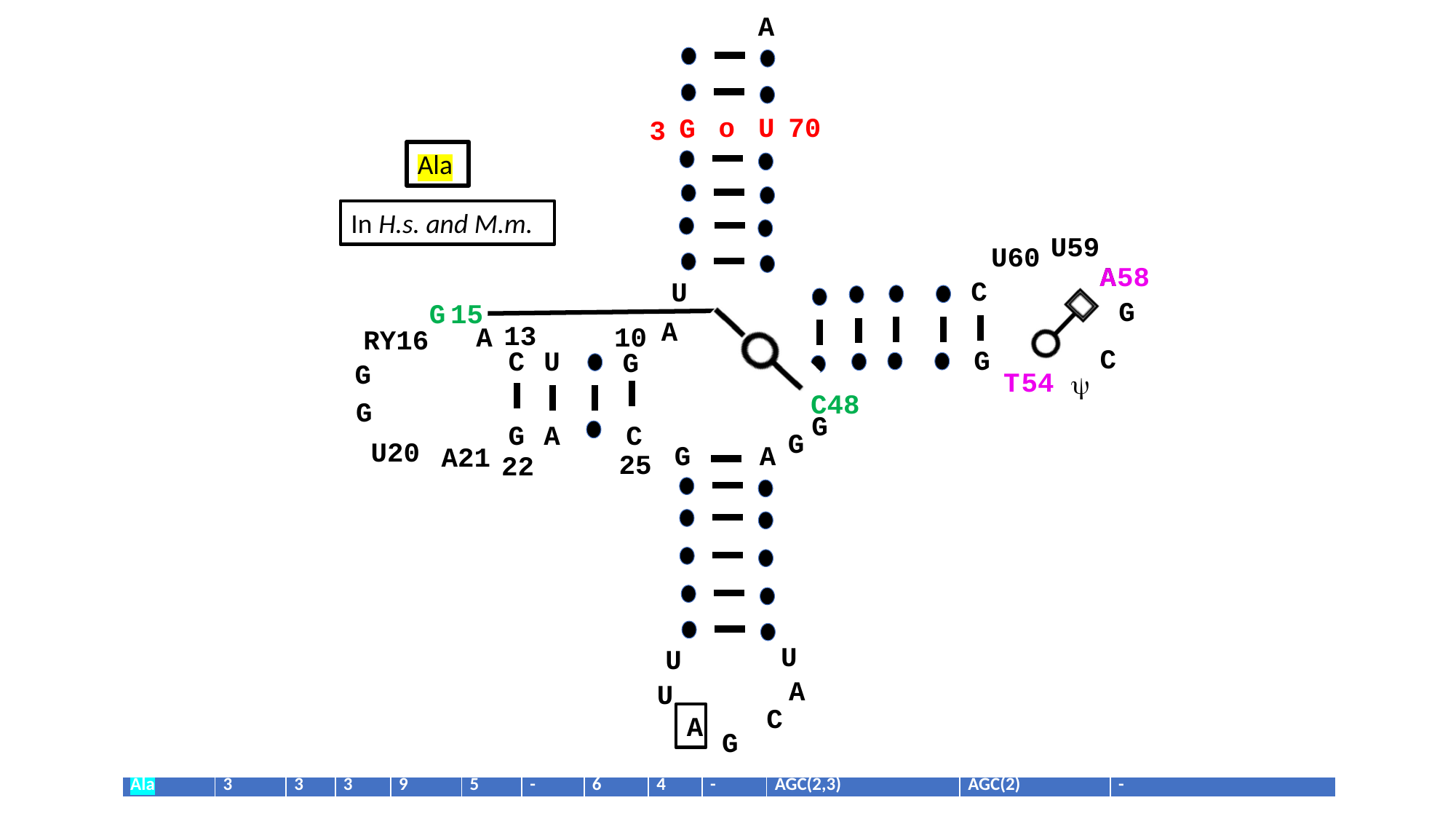

A
o
70
U
G
3
Ala
In H.s. and M.m.
U59
U60
A
58
T
54
A
C
U
G
G
15
C48
A
13
A
10
RY16
©
C
G
C
G
G
y
U
A
G
G
C
G
G
U20
A
G
A21
25
22
U
U
A
U
C
A
G
| Ala | 3 | 3 | 3 | 9 | 5 | - | 6 | 4 | - | AGC(2,3) | AGC(2) | - |
| --- | --- | --- | --- | --- | --- | --- | --- | --- | --- | --- | --- | --- |

## Slide 3
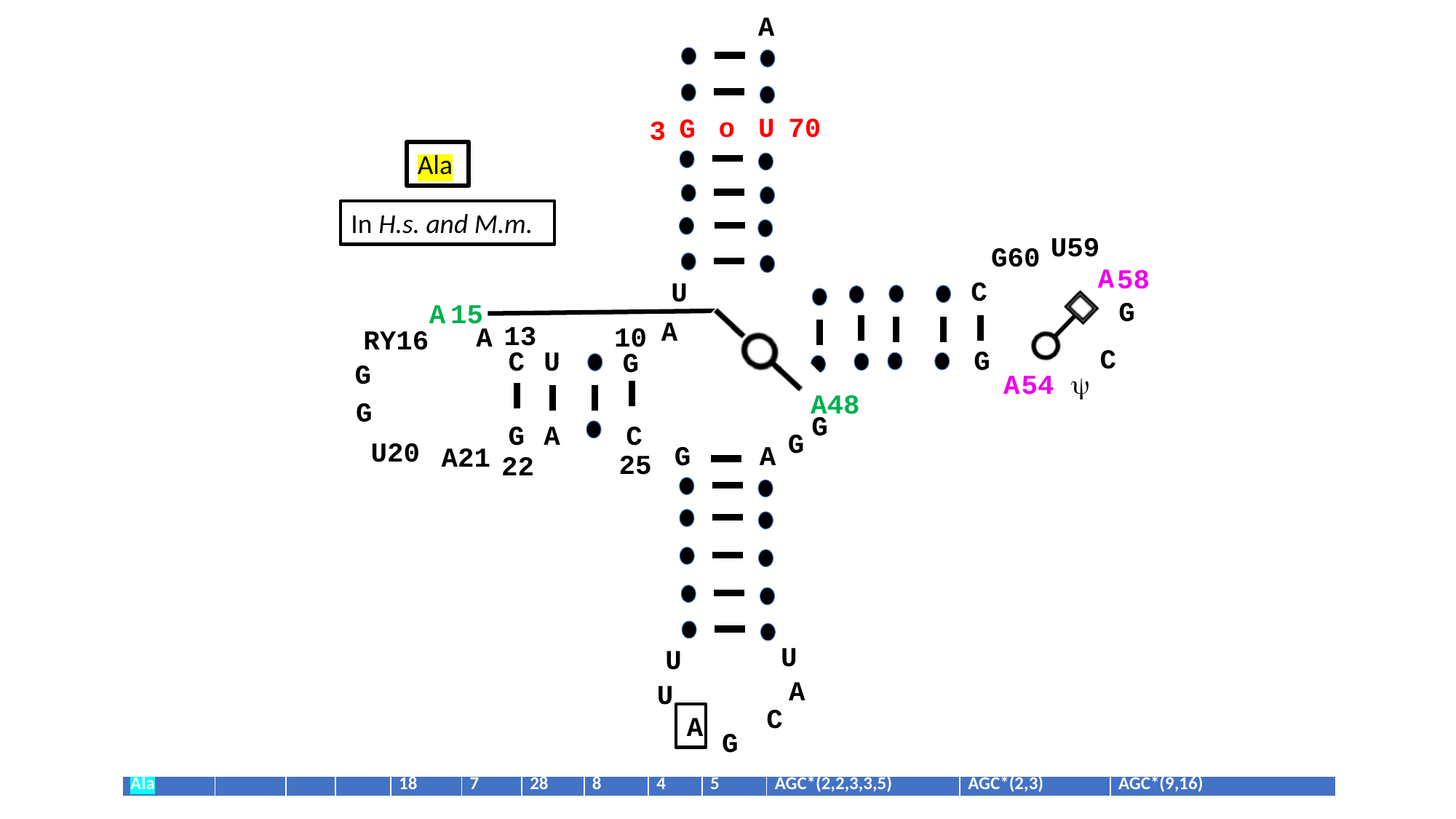

A
o
70
U
G
3
Ala
In H.s. and M.m.
U59
G60
A
58
A
54
C
U
G
A
15
A48
A
13
A
10
RY16
©
C
G
C
G
G
y
U
A
G
G
C
G
G
U20
A
G
A21
25
22
U
U
A
U
C
A
G
| Ala | | | | 18 | 7 | 28 | 8 | 4 | 5 | AGC\*(2,2,3,3,5) | AGC\*(2,3) | AGC\*(9,16) |
| --- | --- | --- | --- | --- | --- | --- | --- | --- | --- | --- | --- | --- |

## Slide 4
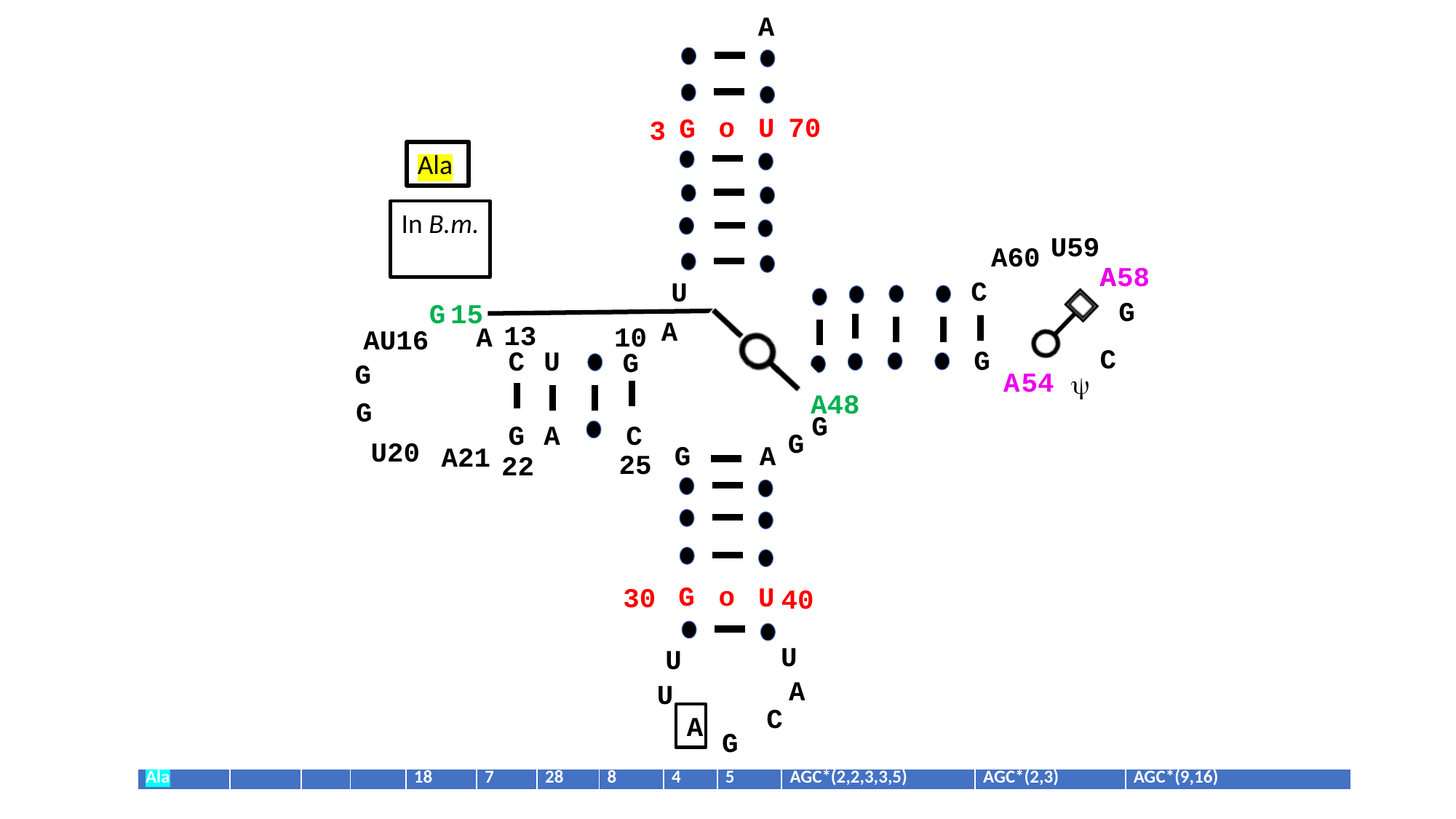

A
o
70
U
G
3
Ala
In B.m.
U59
A60
A
58
A
54
A
C
U
G
G
15
A48
A
13
A
10
AU16
©
C
G
C
G
G
y
U
A
G
G
C
G
G
U20
A
G
A21
25
22
o
G
U
30
40
U
U
A
U
C
A
G
| Ala | | | | 18 | 7 | 28 | 8 | 4 | 5 | AGC\*(2,2,3,3,5) | AGC\*(2,3) | AGC\*(9,16) |
| --- | --- | --- | --- | --- | --- | --- | --- | --- | --- | --- | --- | --- |

## Slide 5
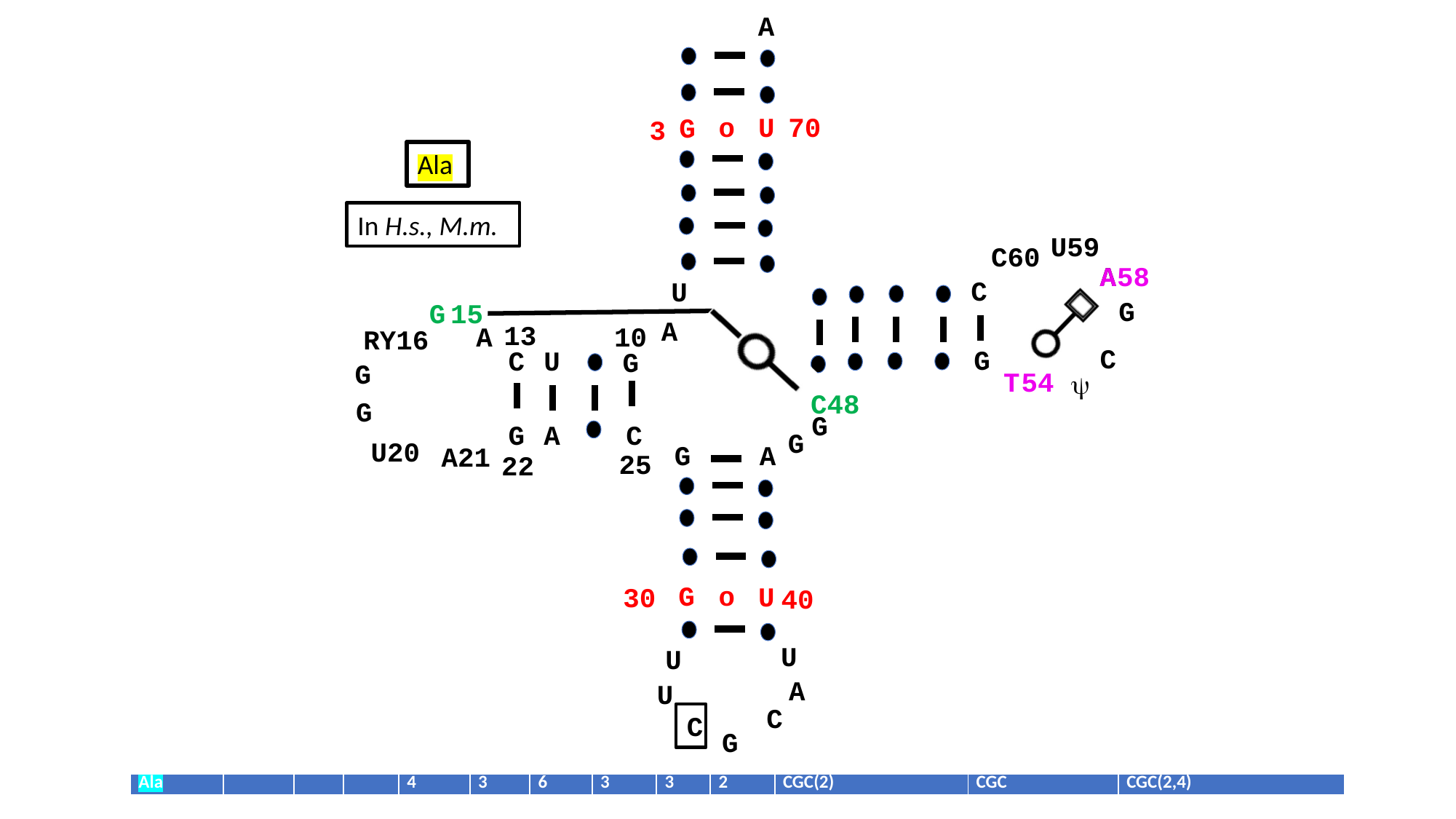

A
o
70
U
G
3
Ala
In H.s., M.m.
U59
C60
A
58
T
54
A
C
U
G
G
15
C48
A
13
A
10
RY16
©
C
G
C
G
G
y
U
A
G
G
C
G
G
U20
A
G
A21
25
22
o
G
U
30
40
U
U
A
U
C
C
G
| Ala | | | | 4 | 3 | 6 | 3 | 3 | 2 | CGC(2) | CGC | CGC(2,4) |
| --- | --- | --- | --- | --- | --- | --- | --- | --- | --- | --- | --- | --- |

## Slide 6
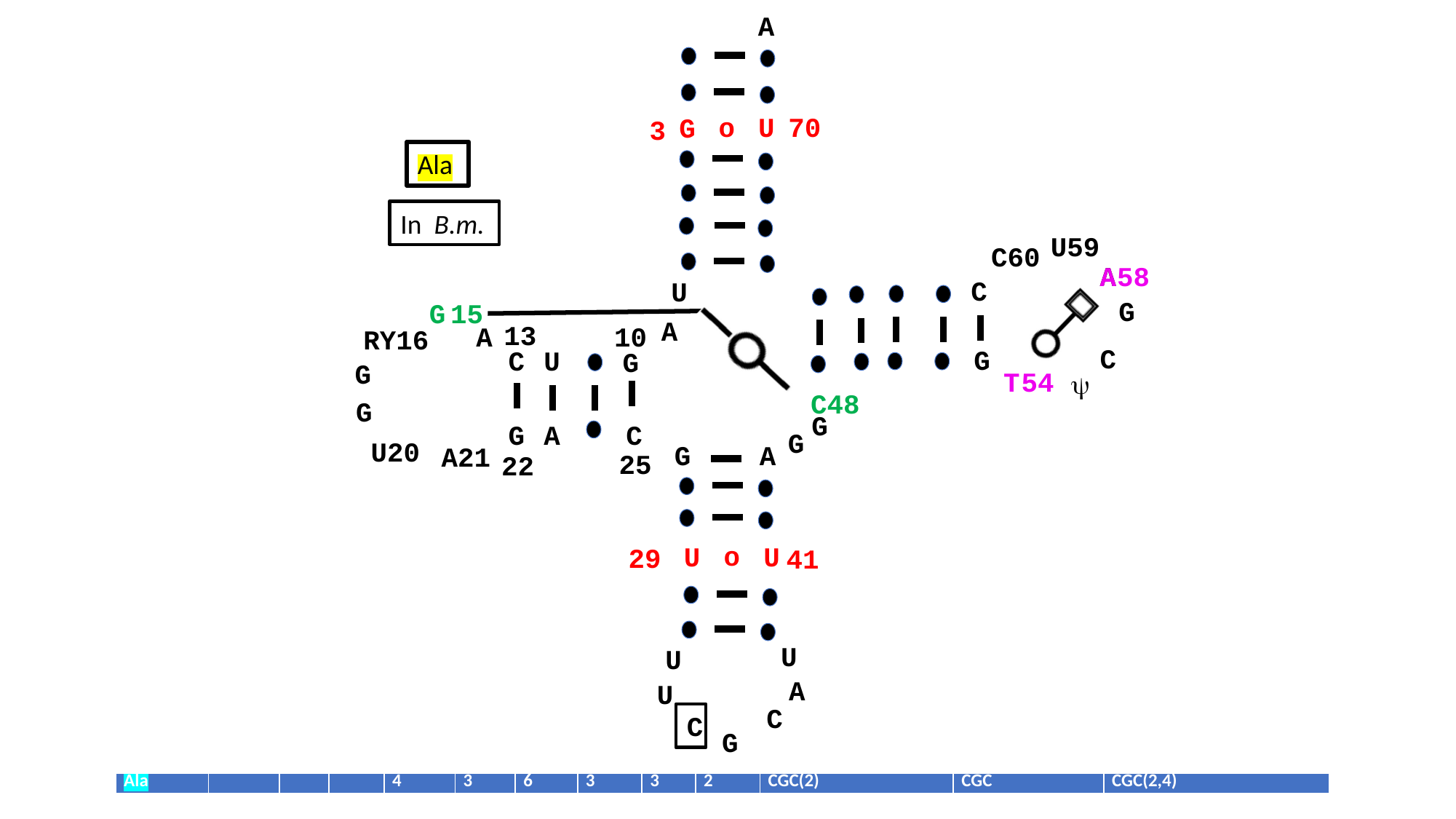

A
o
70
U
G
3
Ala
In B.m.
U59
C60
A
58
T
54
A
C
U
G
G
15
C48
A
13
A
10
RY16
©
C
G
C
G
G
y
U
A
G
G
C
G
G
U20
A
G
A21
25
22
o
U
U
29
41
U
U
A
U
C
C
G
| Ala | | | | 4 | 3 | 6 | 3 | 3 | 2 | CGC(2) | CGC | CGC(2,4) |
| --- | --- | --- | --- | --- | --- | --- | --- | --- | --- | --- | --- | --- |

## Slide 7
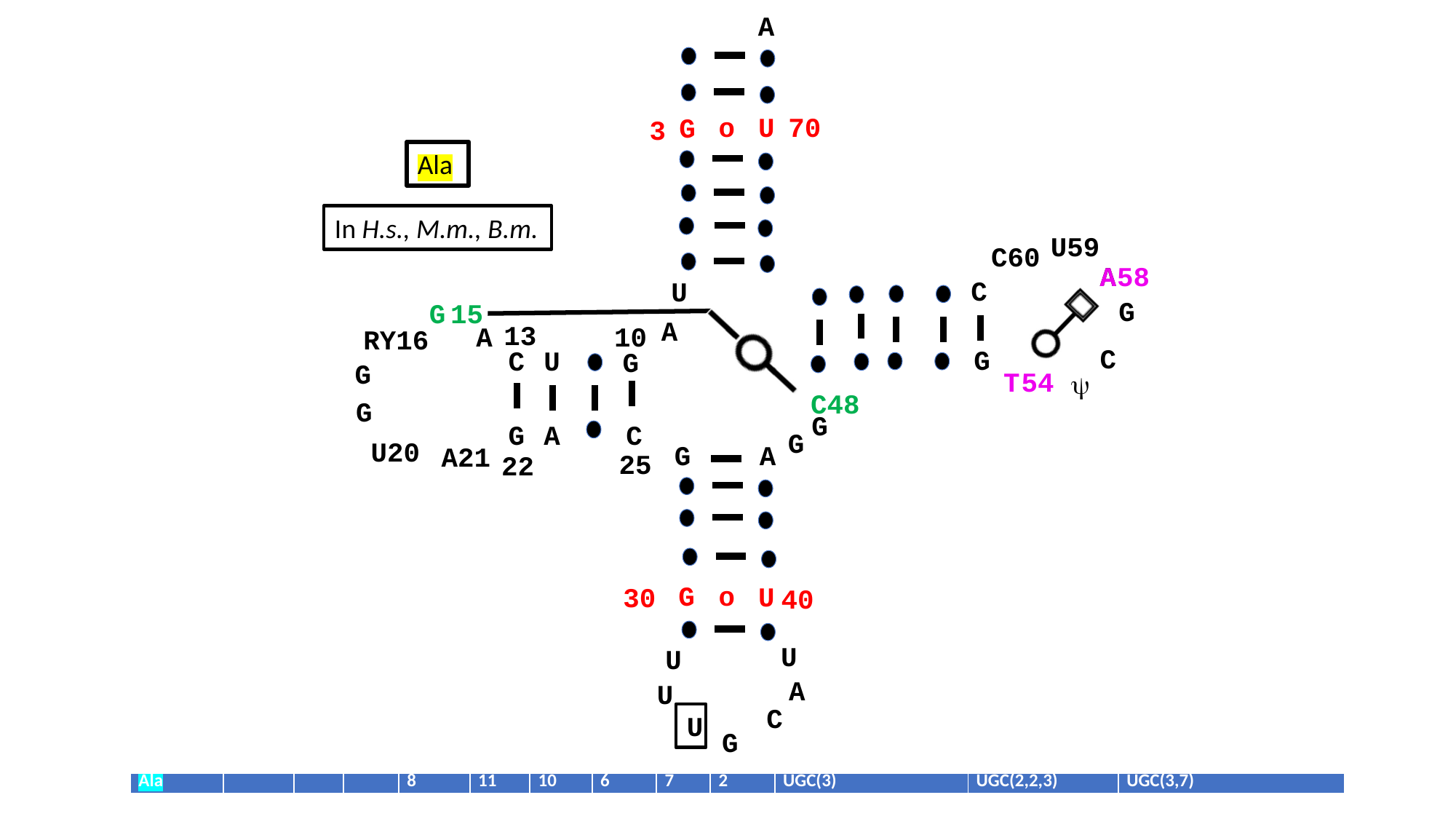

A
o
70
U
G
3
Ala
In H.s., M.m., B.m.
U59
C60
A
58
T
54
A
C
U
G
G
15
C48
A
13
A
10
RY16
©
C
G
C
G
G
y
U
A
G
G
C
G
G
U20
A
G
A21
25
22
o
G
U
30
40
U
U
A
U
C
U
G
| Ala | | | | 8 | 11 | 10 | 6 | 7 | 2 | UGC(3) | UGC(2,2,3) | UGC(3,7) |
| --- | --- | --- | --- | --- | --- | --- | --- | --- | --- | --- | --- | --- |

## Slide 8
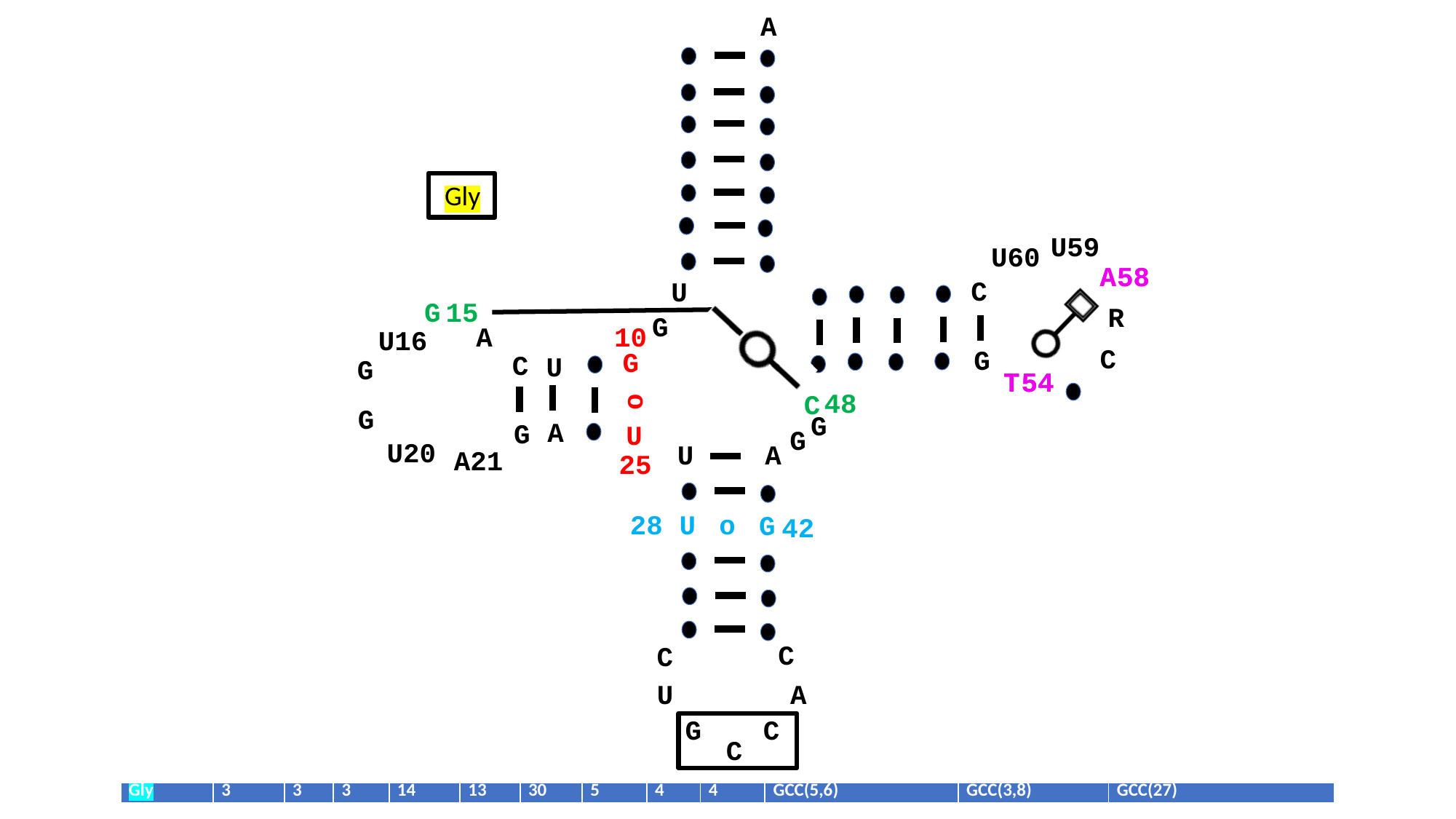

A
Gly
U59
U60
A
58
T
54
A
58
C
U
G
15
R
G
A
10
U16
C
G
G
C
U
G
T
54
48
C
o
G
G
A
G
U
G
U20
U
A
A21
25
o
28
U
G
42
C
C
U
A
C
G
C
| Gly | 3 | 3 | 3 | 14 | 13 | 30 | 5 | 4 | 4 | GCC(5,6) | GCC(3,8) | GCC(27) |
| --- | --- | --- | --- | --- | --- | --- | --- | --- | --- | --- | --- | --- |

## Slide 9
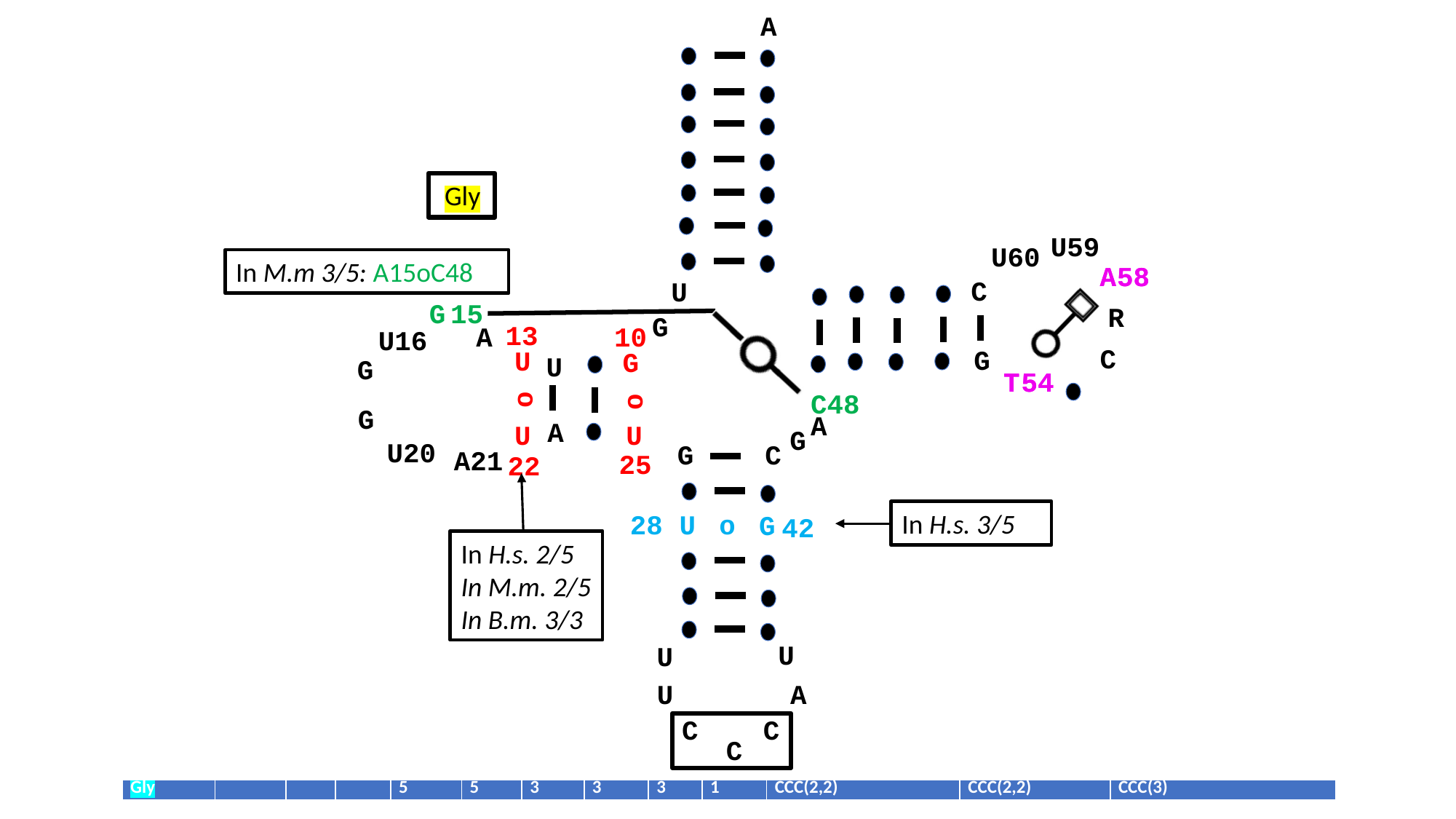

A
Gly
U59
U60
In M.m 3/5: A15oC48
A
58
T
54
A
58
C
U
G
15
C48
R
G
A
10
U16
C
G
G
U
G
T
54
U
U
o
22
13
o
G
A
A
U
G
U20
G
C
A21
25
In H.s. 3/5
o
28
U
G
42
In H.s. 2/5
In M.m. 2/5
In B.m. 3/3
U
U
U
A
C
C
C
| Gly | | | | 5 | 5 | 3 | 3 | 3 | 1 | CCC(2,2) | CCC(2,2) | CCC(3) |
| --- | --- | --- | --- | --- | --- | --- | --- | --- | --- | --- | --- | --- |

## Slide 10
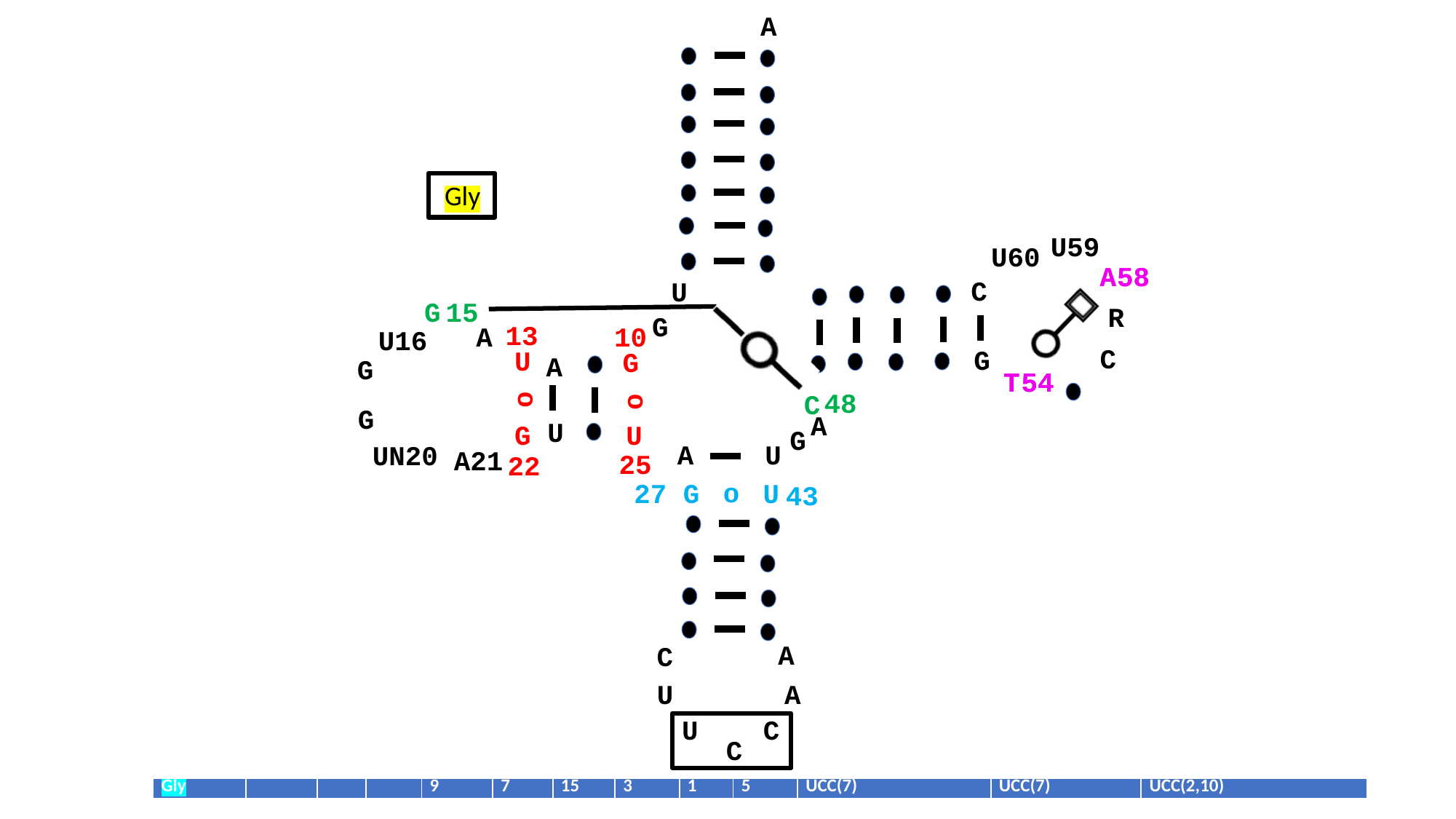

A
Gly
U59
U60
A
58
T
54
A
58
C
U
G
15
R
G
A
10
U16
C
G
G
A
G
T
54
U
G
o
22
13
48
C
o
G
A
U
U
G
A
U
UN20
A21
25
o
27
G
U
43
A
C
U
A
U
C
C
| Gly | | | | 9 | 7 | 15 | 3 | 1 | 5 | UCC(7) | UCC(7) | UCC(2,10) |
| --- | --- | --- | --- | --- | --- | --- | --- | --- | --- | --- | --- | --- |

## Slide 11
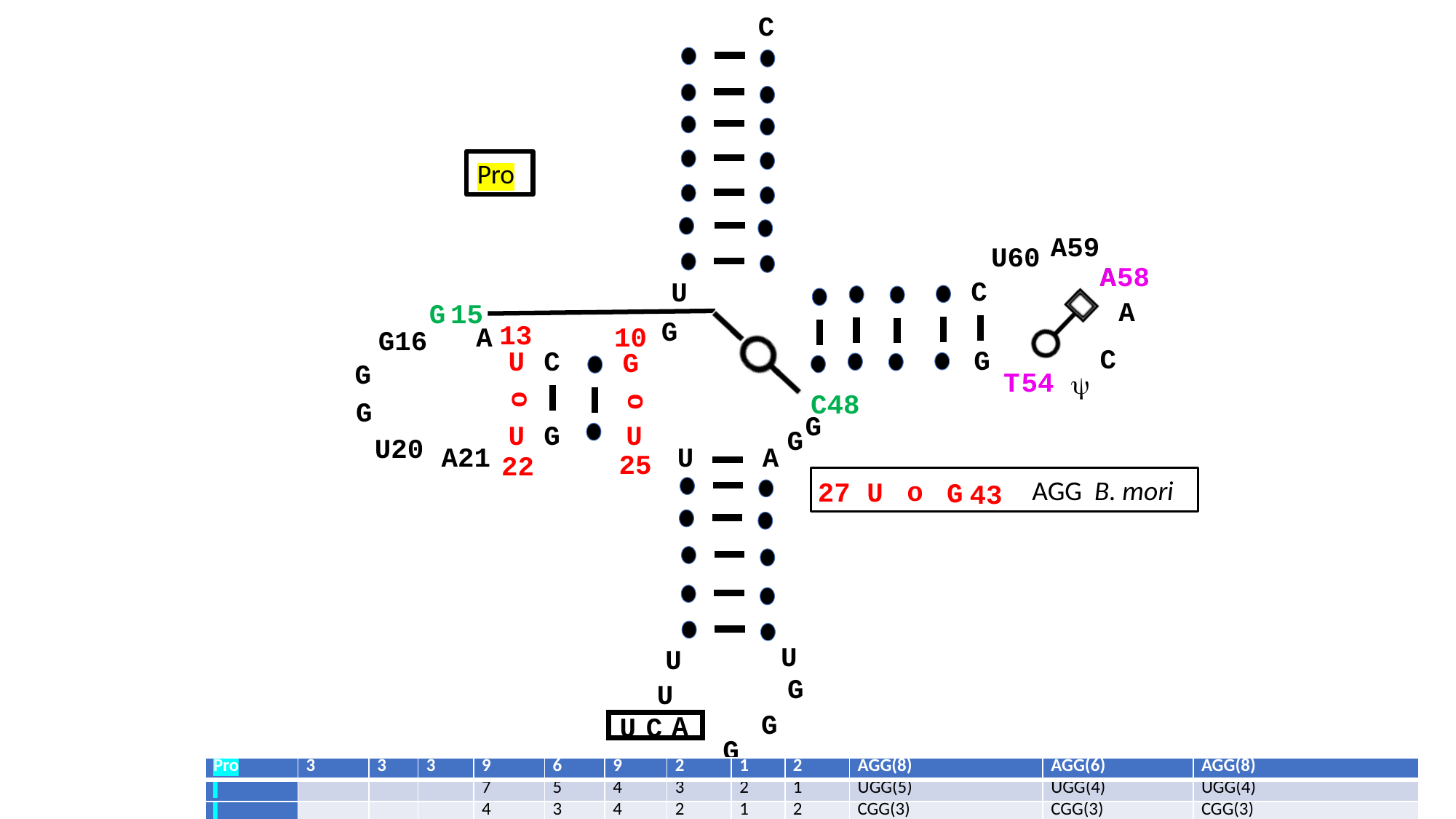

C
Pro
A59
U60
A
58
T
54
A
C
U
A
G
15
C48
G
A
10
G16
©
C
G
G
G
y
U
U
o
22
13
C
G
o
G
G
U
G
U20
A21
A
U
25
 AGG B. mori
o
27
U
G
43
U
U
G
U
G
A
C
U
G
| Pro | 3 | 3 | 3 | 9 | 6 | 9 | 2 | 1 | 2 | AGG(8) | AGG(6) | AGG(8) |
| --- | --- | --- | --- | --- | --- | --- | --- | --- | --- | --- | --- | --- |
| | | | | 7 | 5 | 4 | 3 | 2 | 1 | UGG(5) | UGG(4) | UGG(4) |
| | | | | 4 | 3 | 4 | 2 | 1 | 2 | CGG(3) | CGG(3) | CGG(3) |

## Slide 12
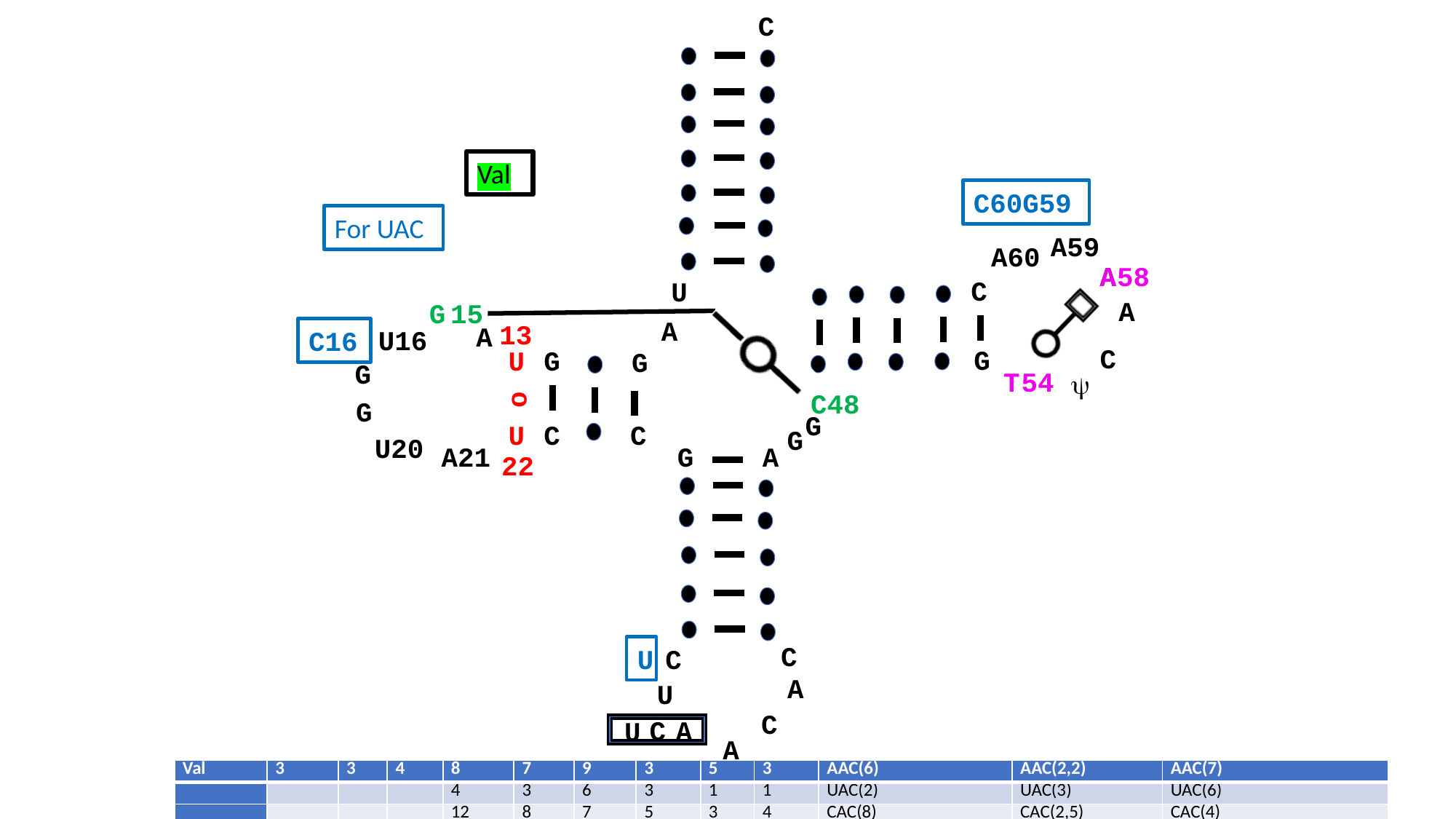

C
Val
C60G59
For UAC
A59
A60
A
58
T
54
A
C
U
A
G
15
C48
A
A
U16
C16
©
C
G
G
G
y
U
U
o
22
13
G
C
G
G
C
G
U20
A21
A
G
C
U
C
A
U
C
C
A
U
A
| Val | 3 | 3 | 4 | 8 | 7 | 9 | 3 | 5 | 3 | AAC(6) | AAC(2,2) | AAC(7) |
| --- | --- | --- | --- | --- | --- | --- | --- | --- | --- | --- | --- | --- |
| | | | | 4 | 3 | 6 | 3 | 1 | 1 | UAC(2) | UAC(3) | UAC(6) |
| | | | | 12 | 8 | 7 | 5 | 3 | 4 | CAC(8) | CAC(2,5) | CAC(4) |
| | | | | - | - | 1 | - | - | 1 | - | - | GAC |

## Slide 13
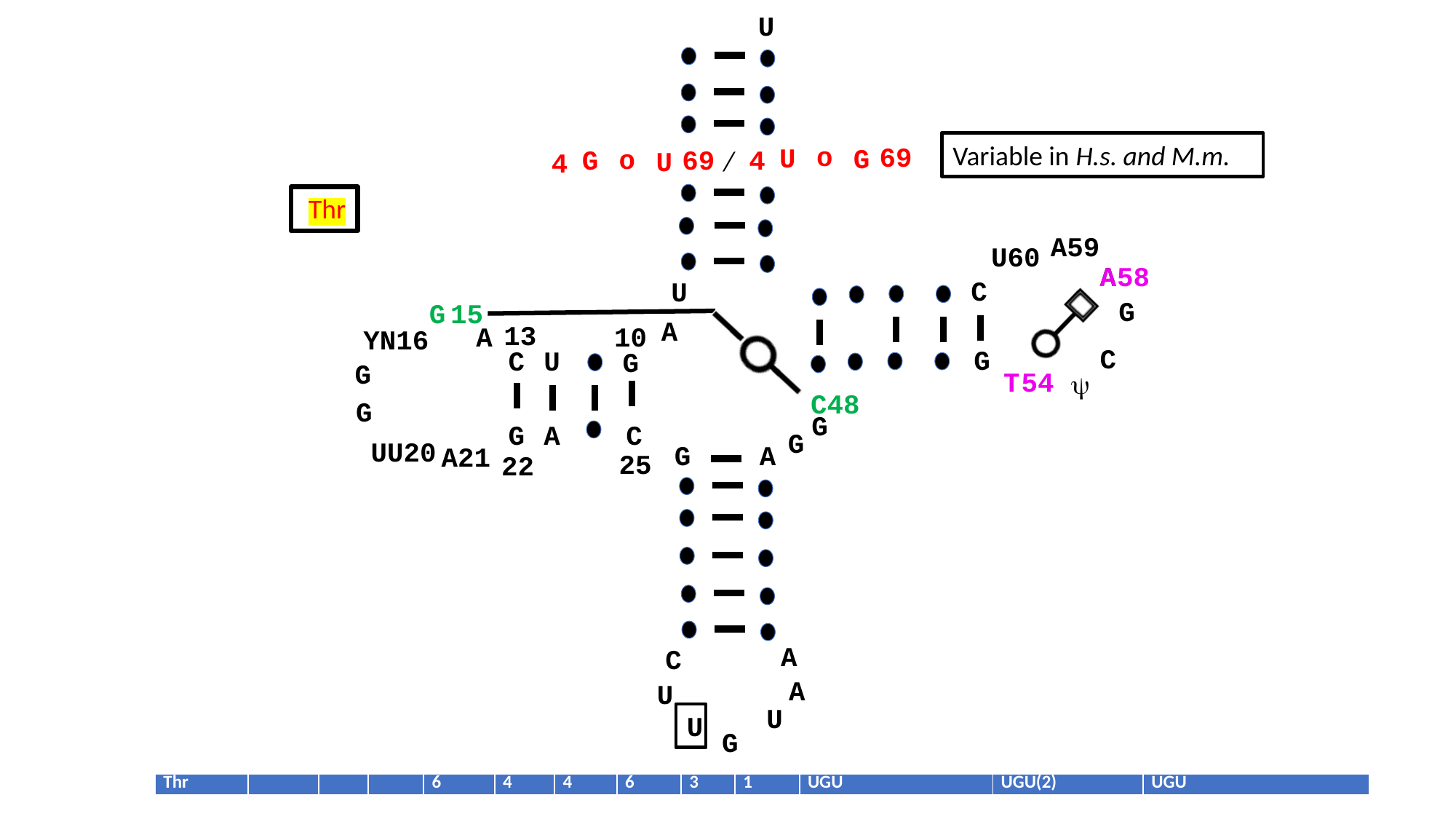

U
Variable in H.s. and M.m.
o
69
U
G
4
o
69
G
U
4
/
Thr
A59
U60
A
58
T
54
A
C
U
G
G
15
C48
A
13
A
10
YN16
©
C
G
C
G
G
y
U
A
G
G
C
G
G
UU20
A
G
A21
25
22
A
C
A
U
U
U
G
| Thr | | | | 6 | 4 | 4 | 6 | 3 | 1 | UGU | UGU(2) | UGU |
| --- | --- | --- | --- | --- | --- | --- | --- | --- | --- | --- | --- | --- |

## Slide 14
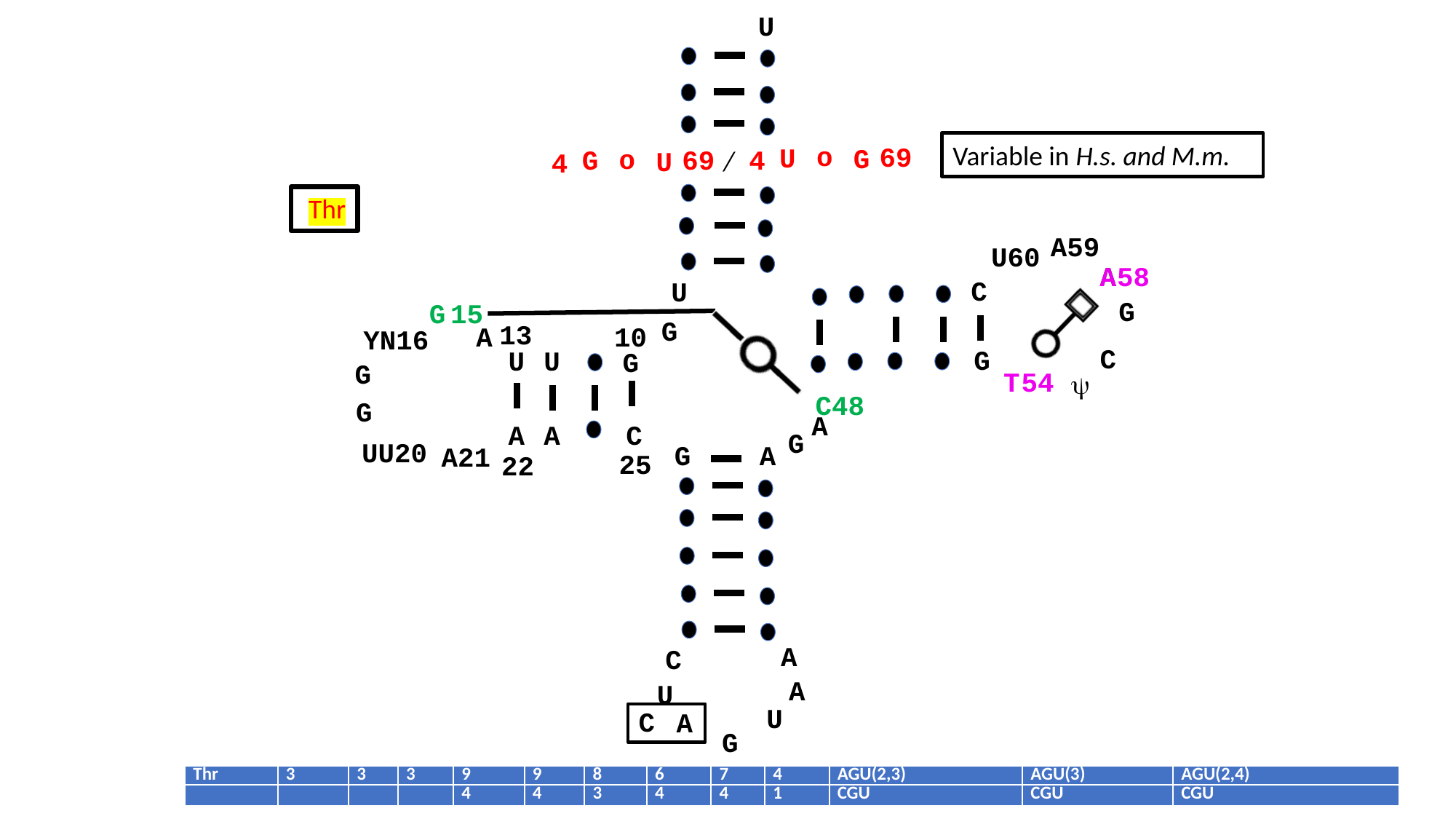

U
Variable in H.s. and M.m.
o
69
U
G
4
o
69
G
U
4
/
Thr
A59
U60
A
58
T
54
A
C
U
G
G
15
C48
G
13
A
10
YN16
©
C
G
U
G
G
y
U
A
G
A
C
A
G
UU20
A
G
A21
25
22
A
C
A
U
U
C
A
G
| Thr | 3 | 3 | 3 | 9 | 9 | 8 | 6 | 7 | 4 | AGU(2,3) | AGU(3) | AGU(2,4) |
| --- | --- | --- | --- | --- | --- | --- | --- | --- | --- | --- | --- | --- |
| | | | | 4 | 4 | 3 | 4 | 4 | 1 | CGU | CGU | CGU |
| --- | --- | --- | --- | --- | --- | --- | --- | --- | --- | --- | --- | --- |

## Slide 15
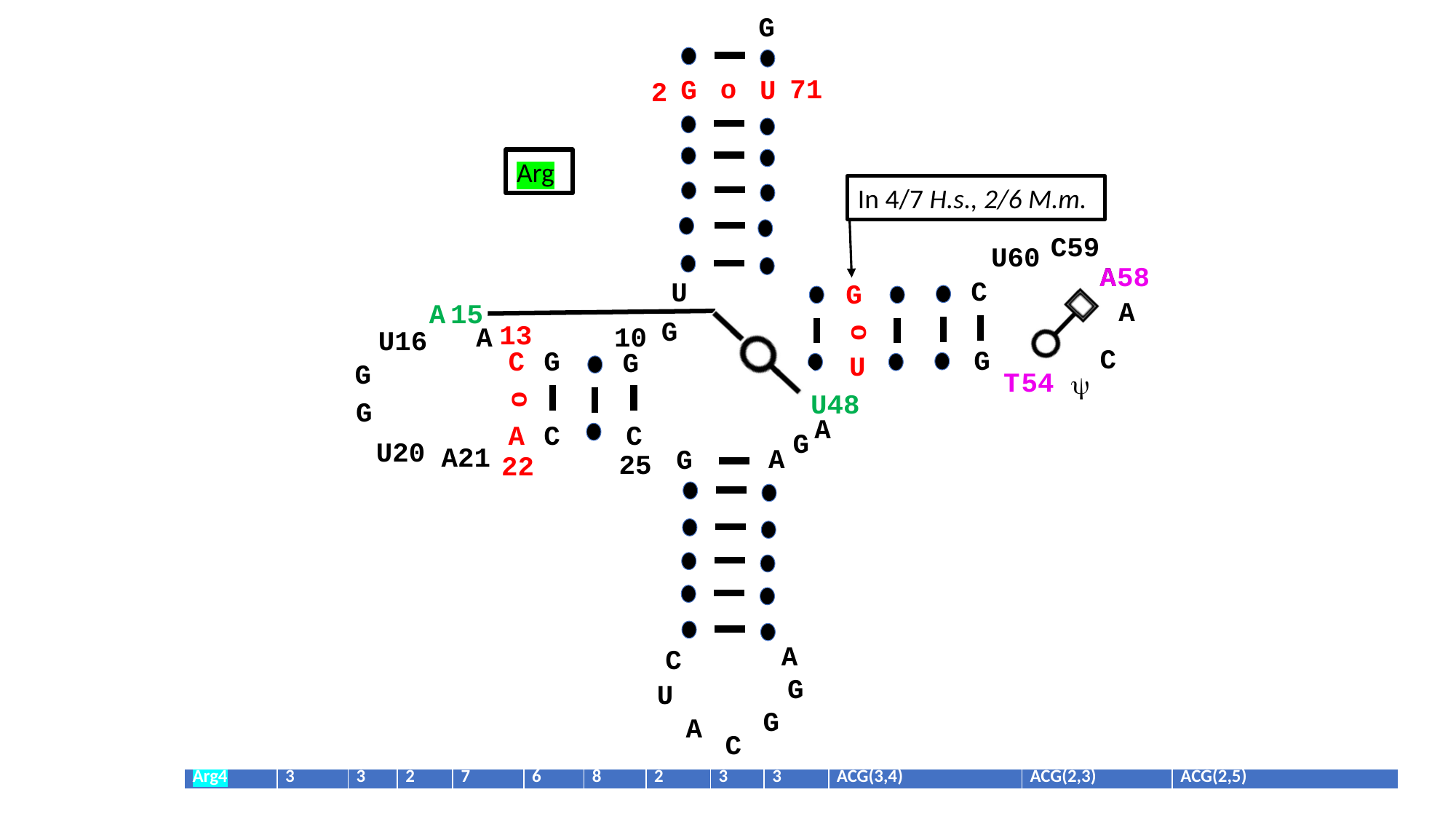

G
o
71
U
G
2
Arg
In 4/7 H.s., 2/6 M.m.
C59
U60
A
58
T
54
A
C
U
G
A
A
15
U48
G
A
10
o
U16
©
C
G
G
U
G
y
C
A
o
22
13
G
C
G
A
C
G
U20
A21
A
G
25
A
C
G
U
G
A
C
| Arg4 | 3 | 3 | 2 | 7 | 6 | 8 | 2 | 3 | 3 | ACG(3,4) | ACG(2,3) | ACG(2,5) |
| --- | --- | --- | --- | --- | --- | --- | --- | --- | --- | --- | --- | --- |

## Slide 16
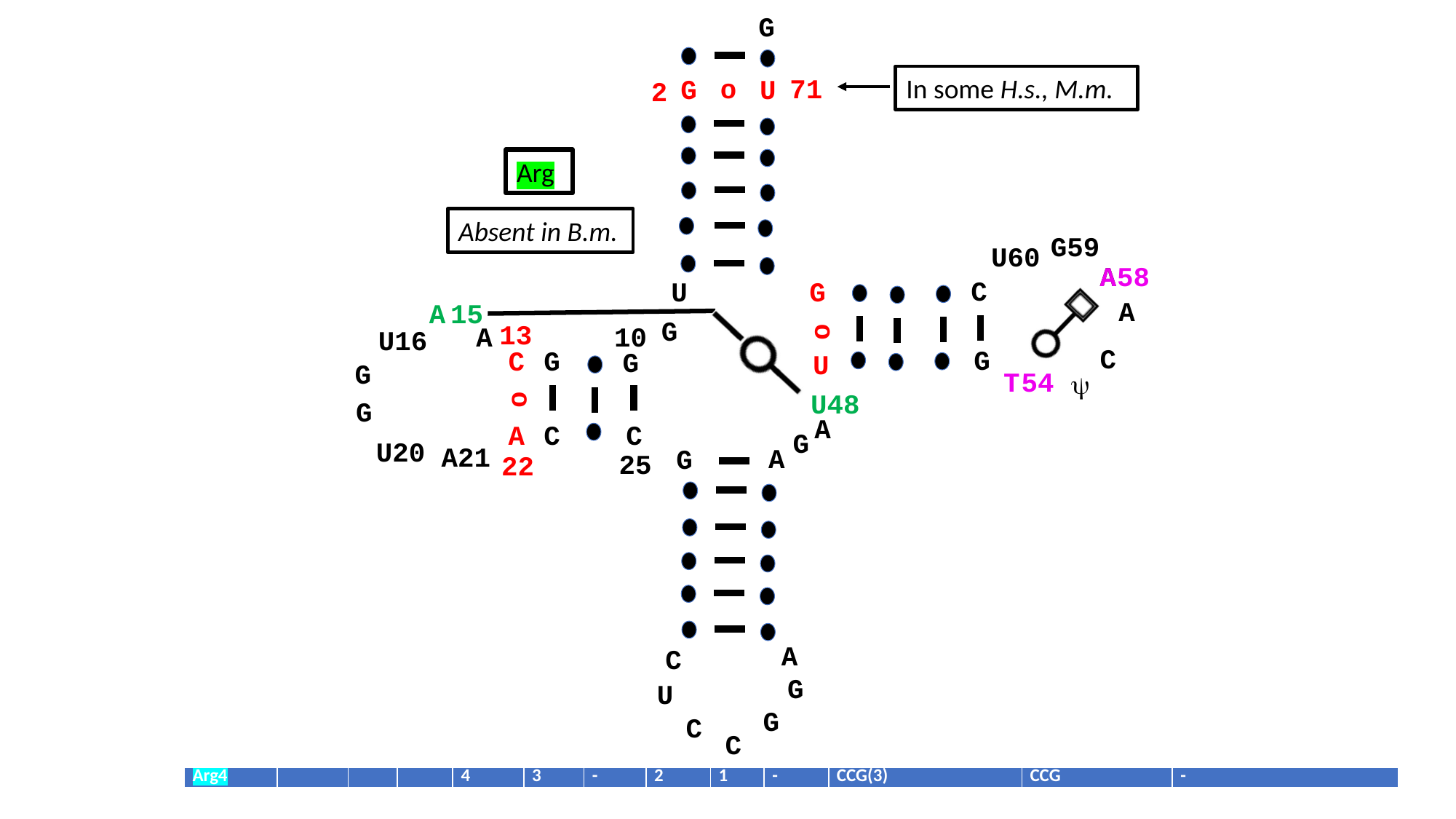

G
o
71
U
G
2
In some H.s., M.m.
Arg
Absent in B.m.
G59
U60
A
58
T
54
A
C
U
G
A
A
15
U48
G
o
A
10
U16
©
C
G
G
U
G
y
C
A
o
22
13
G
C
G
A
C
G
U20
A21
A
G
25
A
C
G
U
G
C
C
| Arg4 | | | | 4 | 3 | - | 2 | 1 | - | CCG(3) | CCG | - |
| --- | --- | --- | --- | --- | --- | --- | --- | --- | --- | --- | --- | --- |

## Slide 17
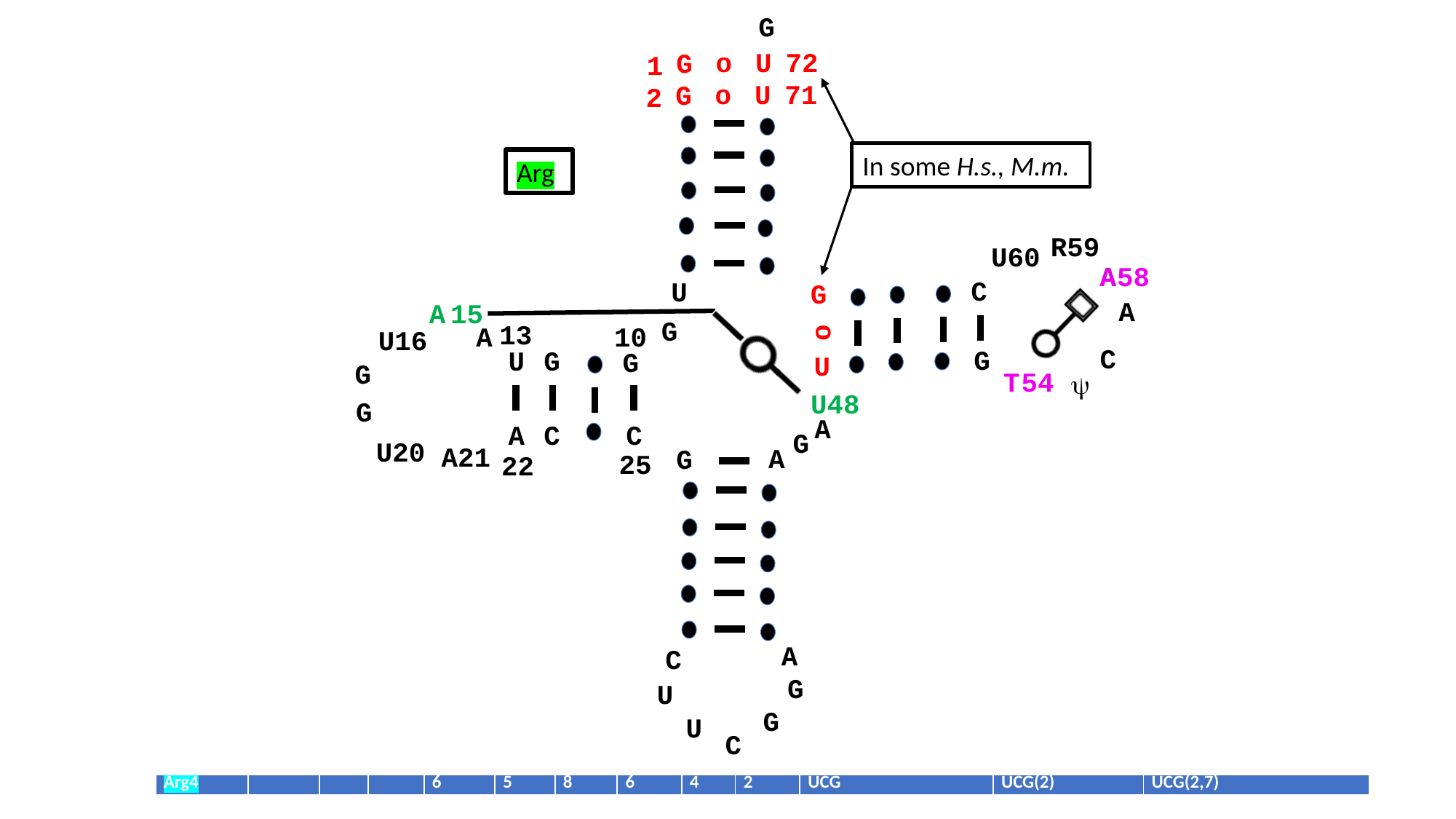

G
o
72
U
G
1
o
71
U
G
2
In some H.s., M.m.
Arg
R59
U60
A
58
T
54
A
C
U
G
A
A
15
U48
G
A
10
o
U16
©
C
G
G
U
G
y
U
A
22
13
G
C
G
A
C
G
U20
A21
A
G
25
A
C
G
U
G
U
C
| Arg4 | | | | 6 | 5 | 8 | 6 | 4 | 2 | UCG | UCG(2) | UCG(2,7) |
| --- | --- | --- | --- | --- | --- | --- | --- | --- | --- | --- | --- | --- |

## Slide 18
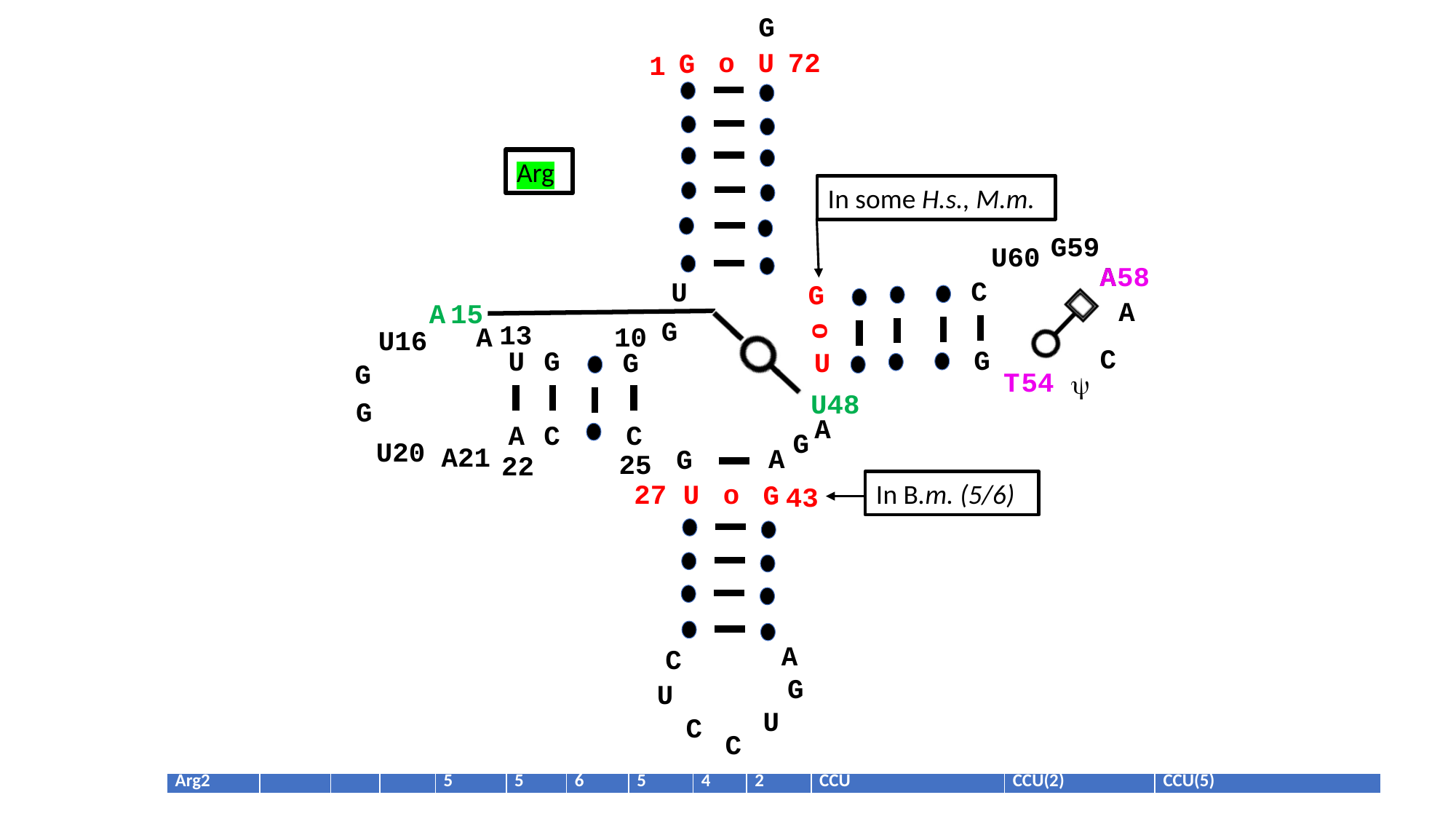

G
o
72
U
G
1
Arg
In some H.s., M.m.
G59
U60
A
58
T
54
A
C
U
G
A
A
15
U48
G
o
A
10
U16
©
C
G
U
G
G
y
U
A
22
13
G
C
G
A
C
G
U20
A21
A
G
25
o
27
U
G
43
In B.m. (5/6)
A
C
G
U
U
C
C
| Arg2 | | | | 5 | 5 | 6 | 5 | 4 | 2 | CCU | CCU(2) | CCU(5) |
| --- | --- | --- | --- | --- | --- | --- | --- | --- | --- | --- | --- | --- |

## Slide 19
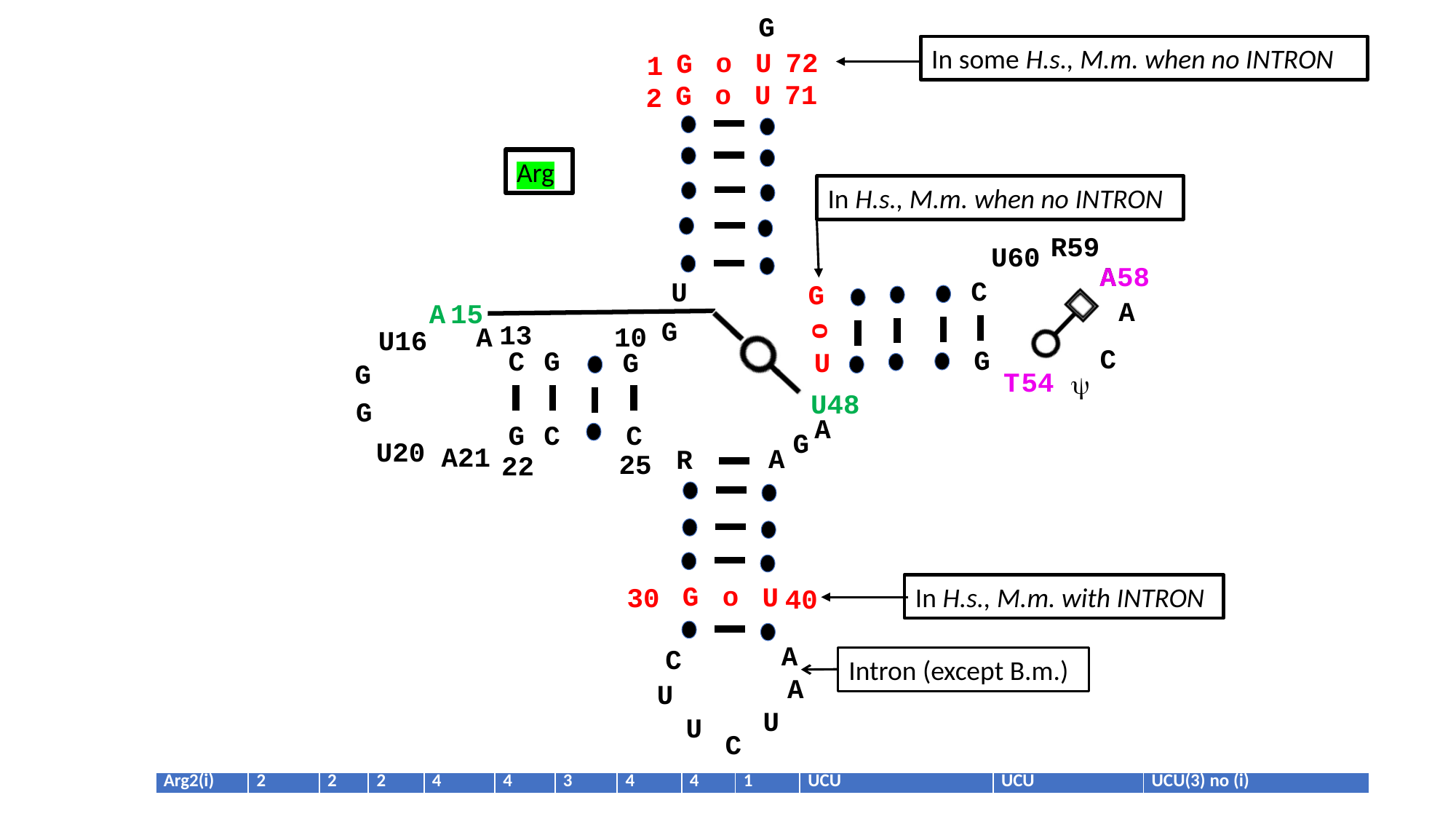

G
In some H.s., M.m. when no INTRON
o
72
U
G
1
o
71
U
G
2
Arg
In H.s., M.m. when no INTRON
R59
U60
A
58
T
54
A
C
U
G
A
A
15
U48
G
o
A
10
U16
©
C
G
U
G
G
y
C
G
22
13
G
C
G
A
C
G
U20
A21
A
R
25
o
G
U
30
40
In H.s., M.m. with INTRON
A
C
Intron (except B.m.)
A
U
U
U
C
| Arg2(i) | 2 | 2 | 2 | 4 | 4 | 3 | 4 | 4 | 1 | UCU | UCU | UCU(3) no (i) |
| --- | --- | --- | --- | --- | --- | --- | --- | --- | --- | --- | --- | --- |

## Slide 20
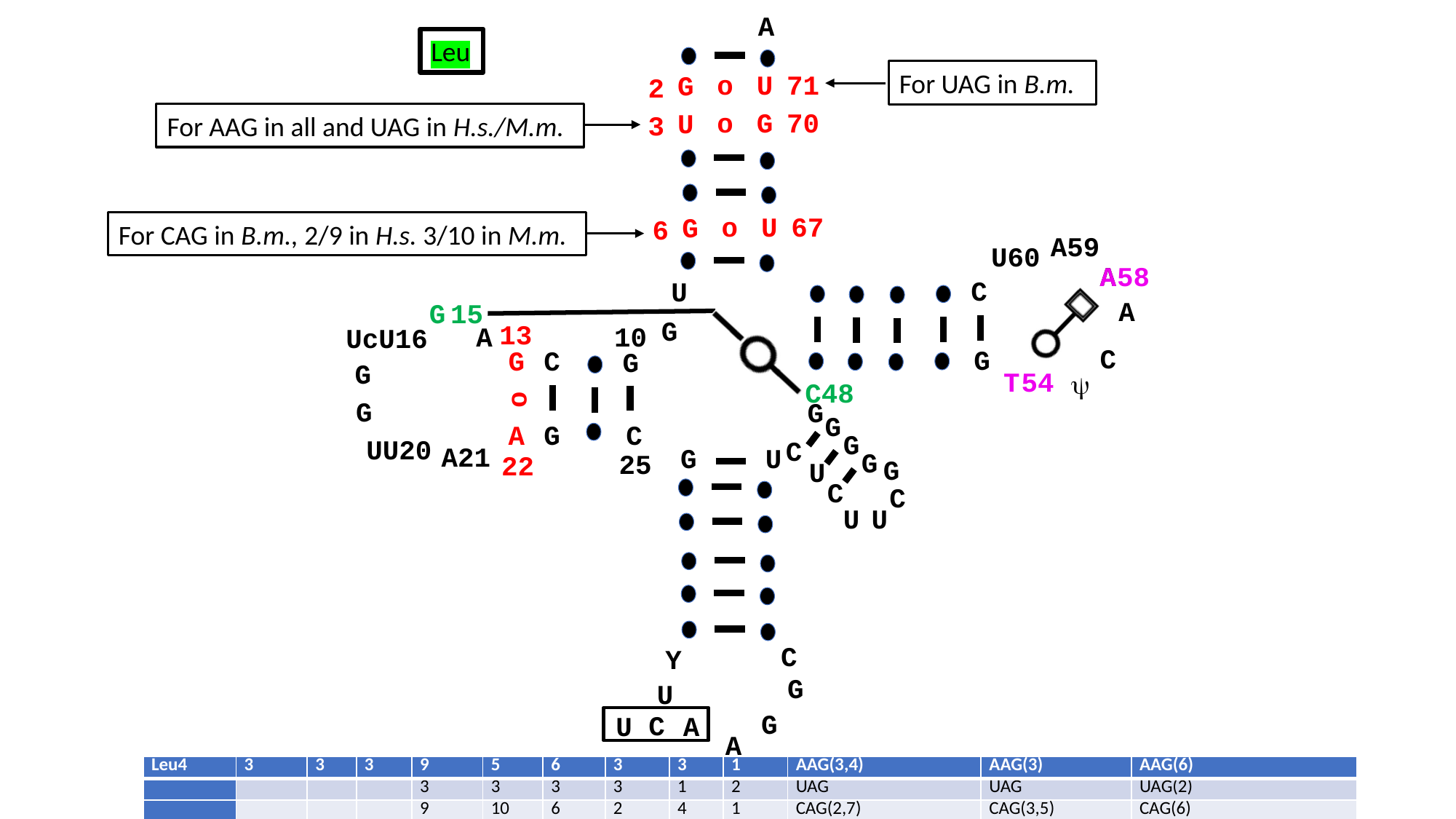

A
Leu
For UAG in B.m.
o
71
U
G
2
o
70
G
U
3
For AAG in all and UAG in H.s./M.m.
o
67
U
G
6
For CAG in B.m., 2/9 in H.s. 3/10 in M.m.
A59
U60
A
58
T
54
A
C
U
A
G
15
C48
G
A
10
UcU16
©
C
G
G
G
y
G
A
o
22
13
C
G
G
G
G
C
G
UU20
C
A21
U
G
G
25
G
U
C
C
U
U
C
Y
G
U
G
C
A
U
A
| Leu4 | 3 | 3 | 3 | 9 | 5 | 6 | 3 | 3 | 1 | AAG(3,4) | AAG(3) | AAG(6) |
| --- | --- | --- | --- | --- | --- | --- | --- | --- | --- | --- | --- | --- |
| | | | | 3 | 3 | 3 | 3 | 1 | 2 | UAG | UAG | UAG(2) |
| | | | | 9 | 10 | 6 | 2 | 4 | 1 | CAG(2,7) | CAG(3,5) | CAG(6) |

## Slide 21
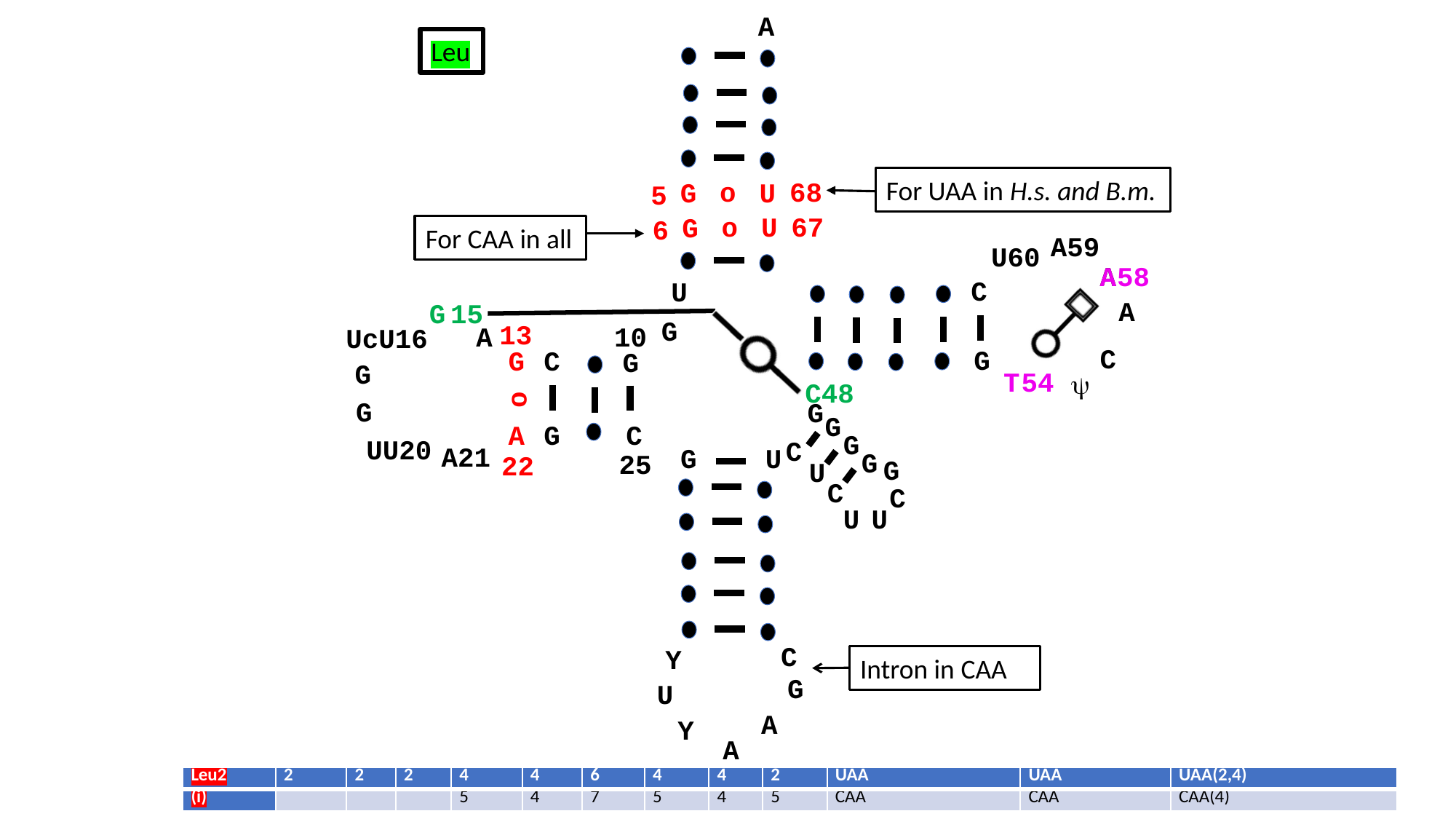

A
Leu
For UAA in H.s. and B.m.
o
68
U
G
5
o
67
U
G
6
For CAA in all
A59
U60
A
58
T
54
A
C
U
A
G
15
C48
G
A
10
UcU16
©
C
G
G
G
y
G
A
o
22
13
C
G
G
G
G
C
G
UU20
C
A21
U
G
G
25
G
U
C
C
U
U
C
Y
Intron in CAA
G
U
A
Y
A
| Leu2 | 2 | 2 | 2 | 4 | 4 | 6 | 4 | 4 | 2 | UAA | UAA | UAA(2,4) |
| --- | --- | --- | --- | --- | --- | --- | --- | --- | --- | --- | --- | --- |
| (i) | | | | 5 | 4 | 7 | 5 | 4 | 5 | CAA | CAA | CAA(4) |

## Slide 22
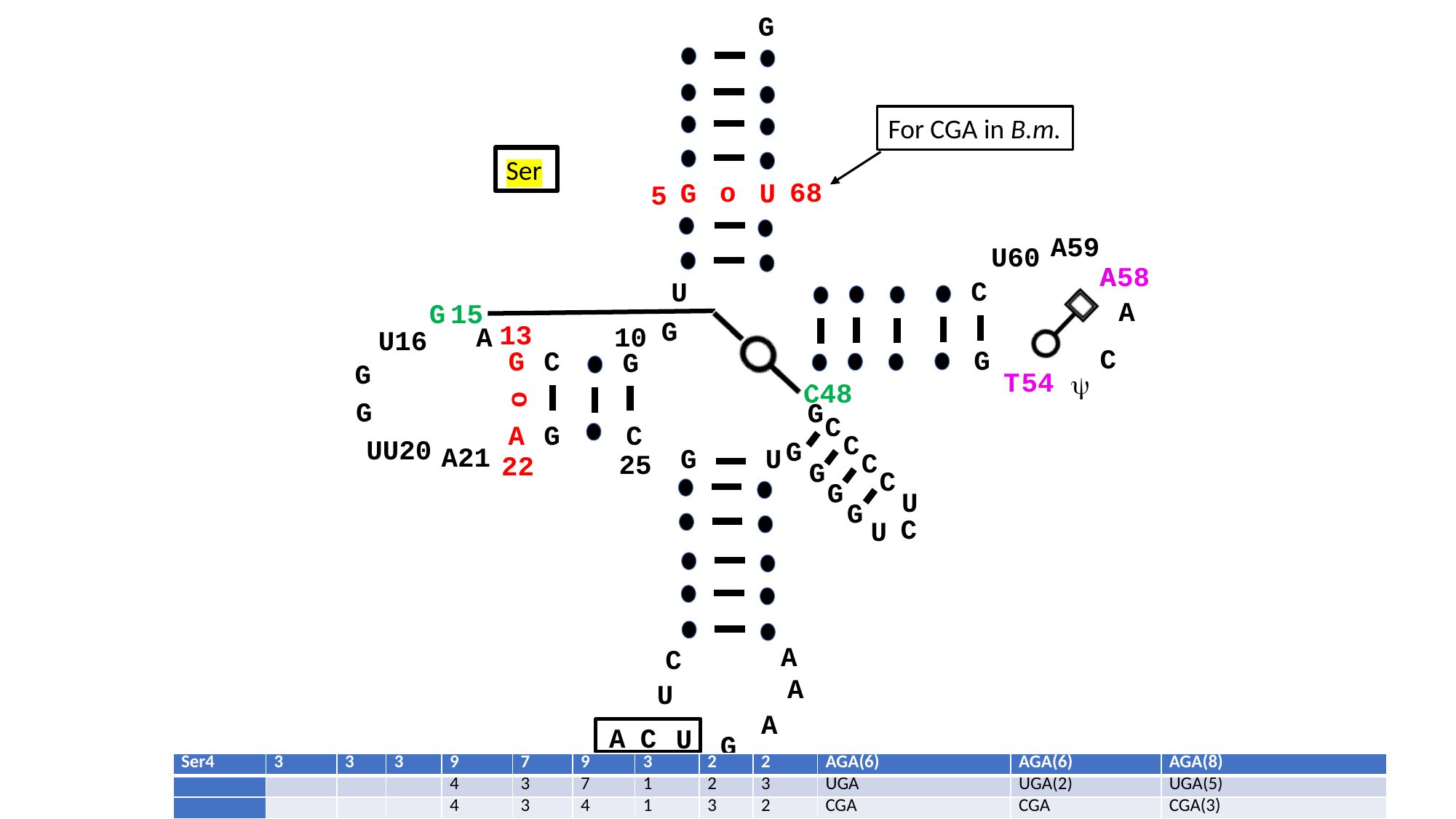

G
For CGA in B.m.
Ser
o
68
U
G
5
A59
U60
A
58
T
54
A
C
U
A
G
15
C48
G
A
10
U16
©
C
G
G
G
y
G
A
o
22
13
C
G
G
G
C
C
C
UU20
G
A21
U
G
C
25
G
C
G
U
G
C
U
A
C
A
U
A
A
C
U
G
| Ser4 | 3 | 3 | 3 | 9 | 7 | 9 | 3 | 2 | 2 | AGA(6) | AGA(6) | AGA(8) |
| --- | --- | --- | --- | --- | --- | --- | --- | --- | --- | --- | --- | --- |
| | | | | 4 | 3 | 7 | 1 | 2 | 3 | UGA | UGA(2) | UGA(5) |
| | | | | 4 | 3 | 4 | 1 | 3 | 2 | CGA | CGA | CGA(3) |

## Slide 23
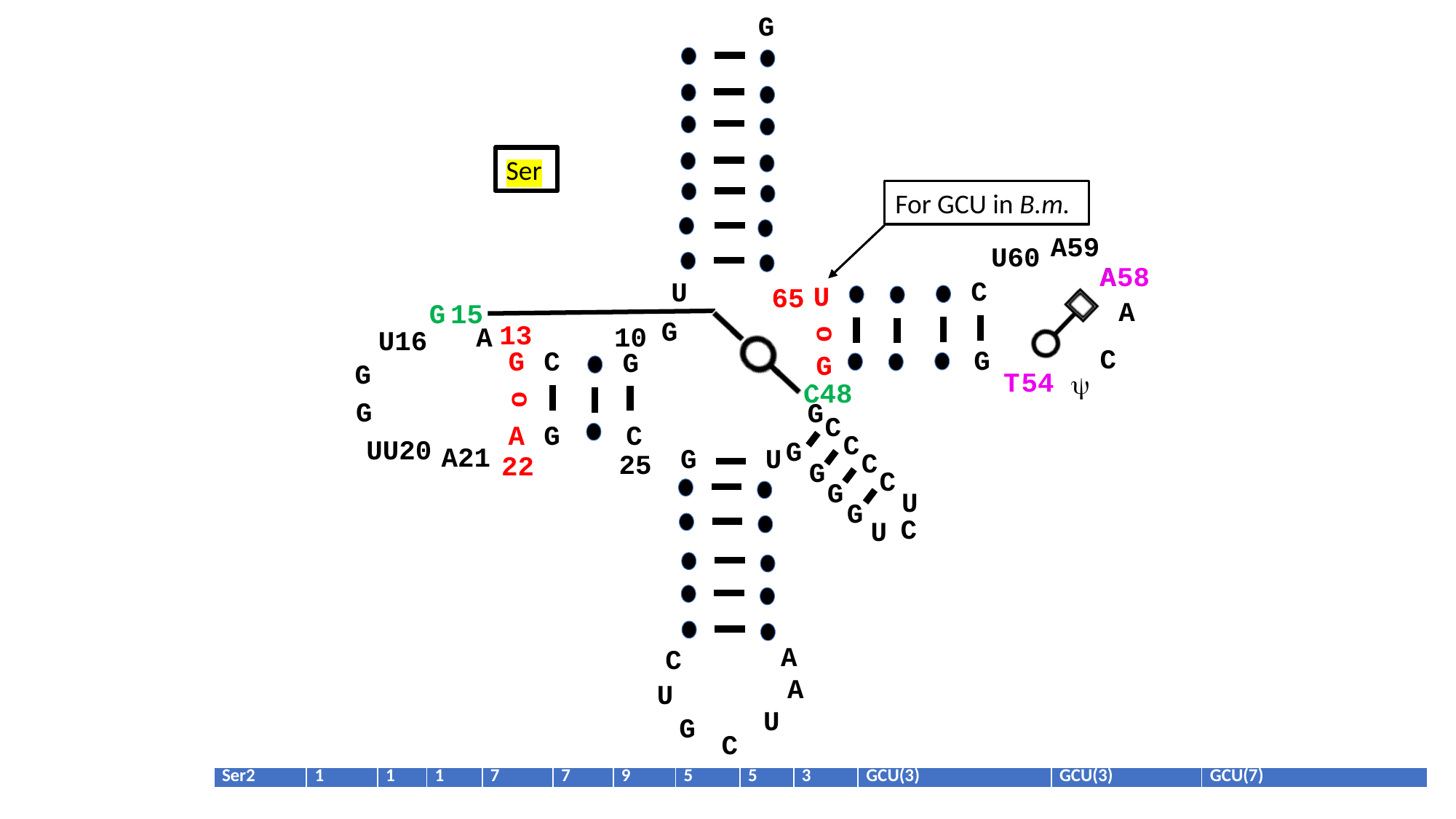

G
Ser
For GCU in B.m.
A59
U60
A
58
T
54
A
C
U
A
65
U
G
o
G
15
C48
G
A
10
U16
©
C
G
G
G
y
G
A
o
22
13
C
G
G
G
C
C
C
UU20
G
A21
U
G
C
25
G
C
G
U
G
C
U
A
C
A
U
U
G
C
| Ser2 | 1 | 1 | 1 | 7 | 7 | 9 | 5 | 5 | 3 | GCU(3) | GCU(3) | GCU(7) |
| --- | --- | --- | --- | --- | --- | --- | --- | --- | --- | --- | --- | --- |

## Slide 24
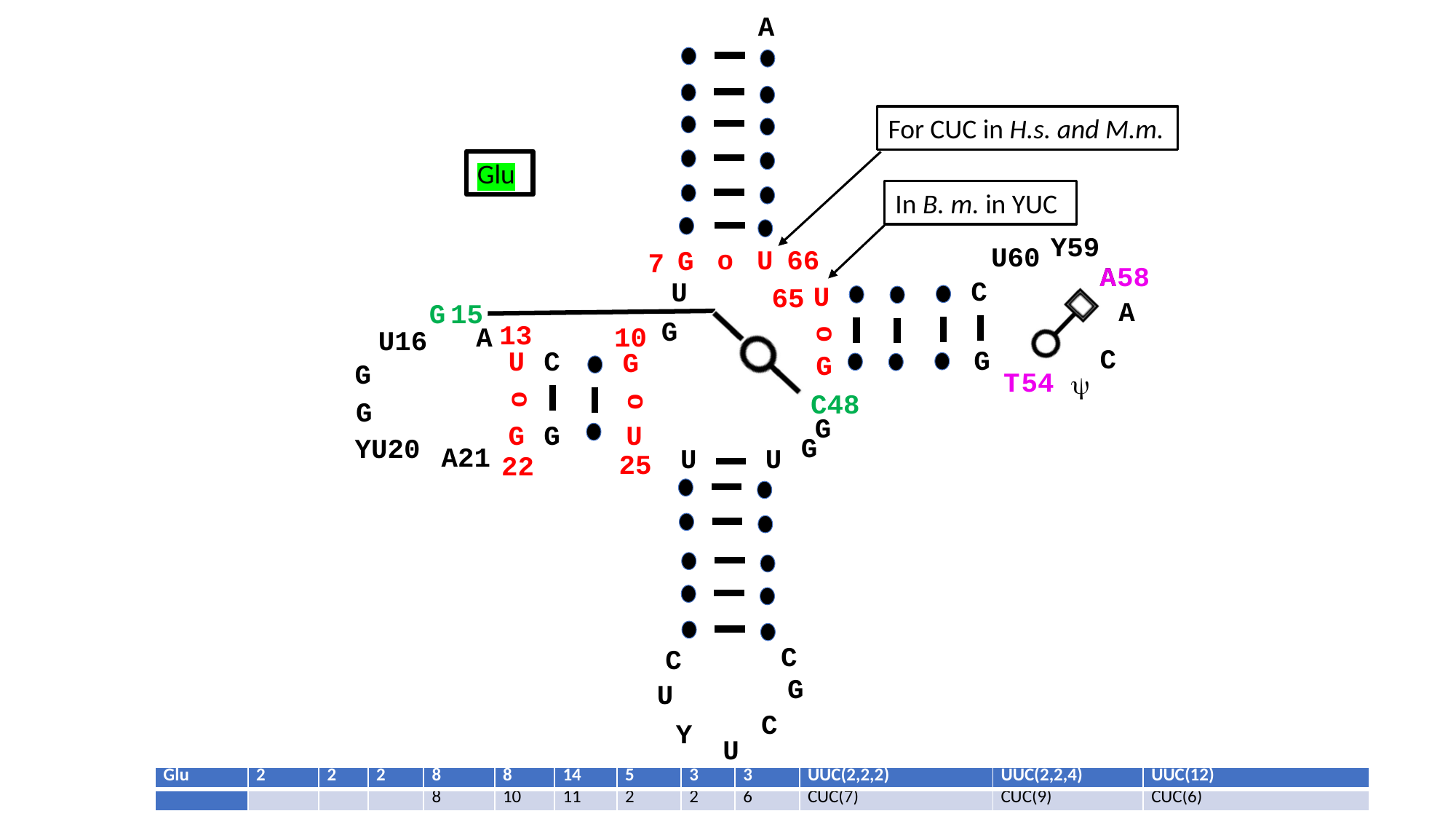

A
For CUC in H.s. and M.m.
Glu
In B. m. in YUC
Y59
U60
o
66
U
G
7
A
58
T
54
A
C
U
A
65
U
G
o
G
15
C48
G
A
10
U16
©
C
G
G
G
y
U
G
o
22
13
C
G
o
G
G
U
G
YU20
A21
U
U
25
C
C
G
U
C
Y
U
| Glu | 2 | 2 | 2 | 8 | 8 | 14 | 5 | 3 | 3 | UUC(2,2,2) | UUC(2,2,4) | UUC(12) |
| --- | --- | --- | --- | --- | --- | --- | --- | --- | --- | --- | --- | --- |
| | | | | 8 | 10 | 11 | 2 | 2 | 6 | CUC(7) | CUC(9) | CUC(6) |

## Slide 25
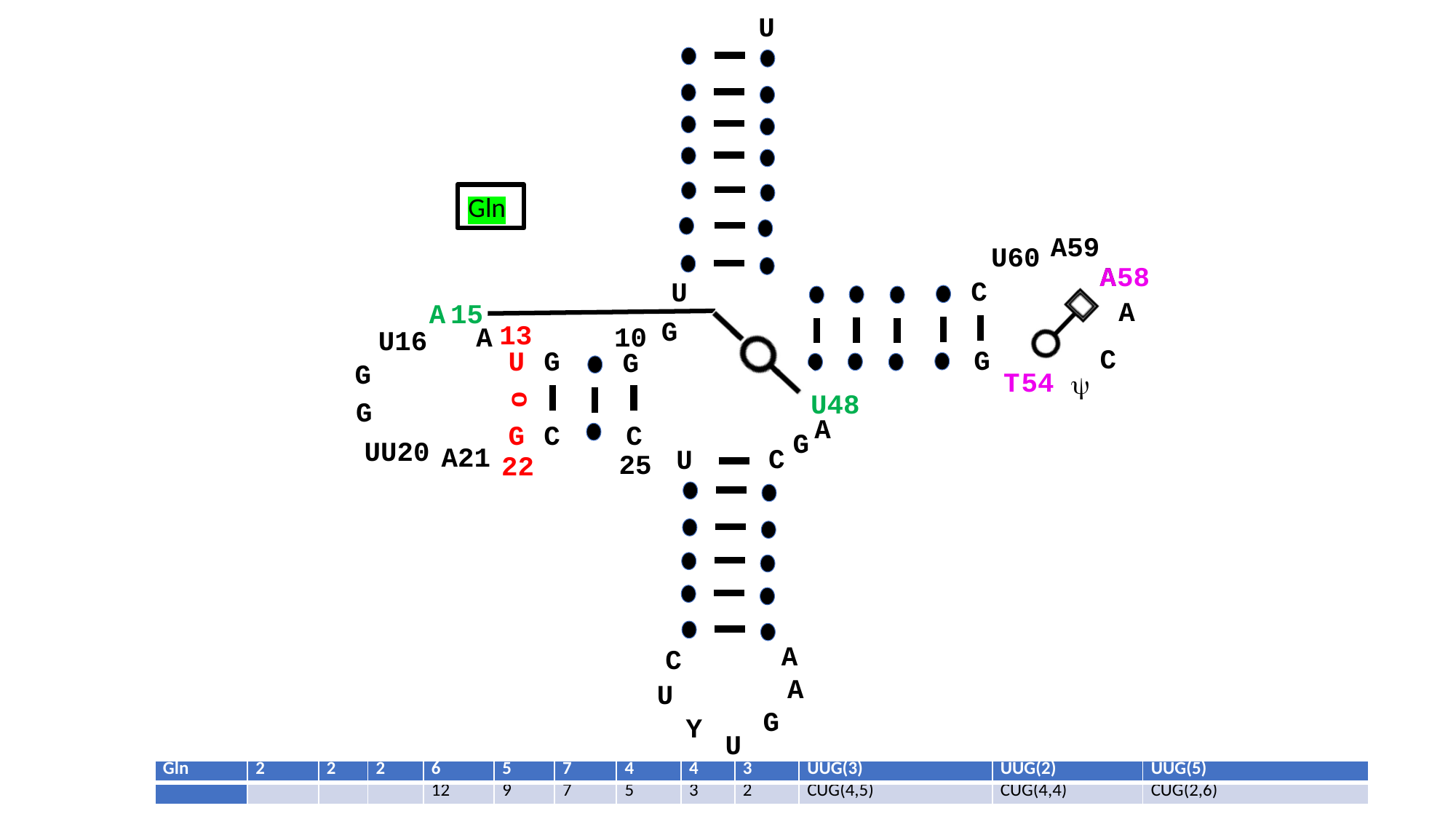

U
Gln
A59
U60
A
58
T
54
A
C
U
A
A
15
U48
G
A
10
U16
©
C
G
G
G
y
U
G
o
22
13
G
C
G
A
C
G
UU20
A21
C
U
25
A
C
A
U
G
Y
U
| Gln | 2 | 2 | 2 | 6 | 5 | 7 | 4 | 4 | 3 | UUG(3) | UUG(2) | UUG(5) |
| --- | --- | --- | --- | --- | --- | --- | --- | --- | --- | --- | --- | --- |
| | | | | 12 | 9 | 7 | 5 | 3 | 2 | CUG(4,5) | CUG(4,4) | CUG(2,6) |

## Slide 26
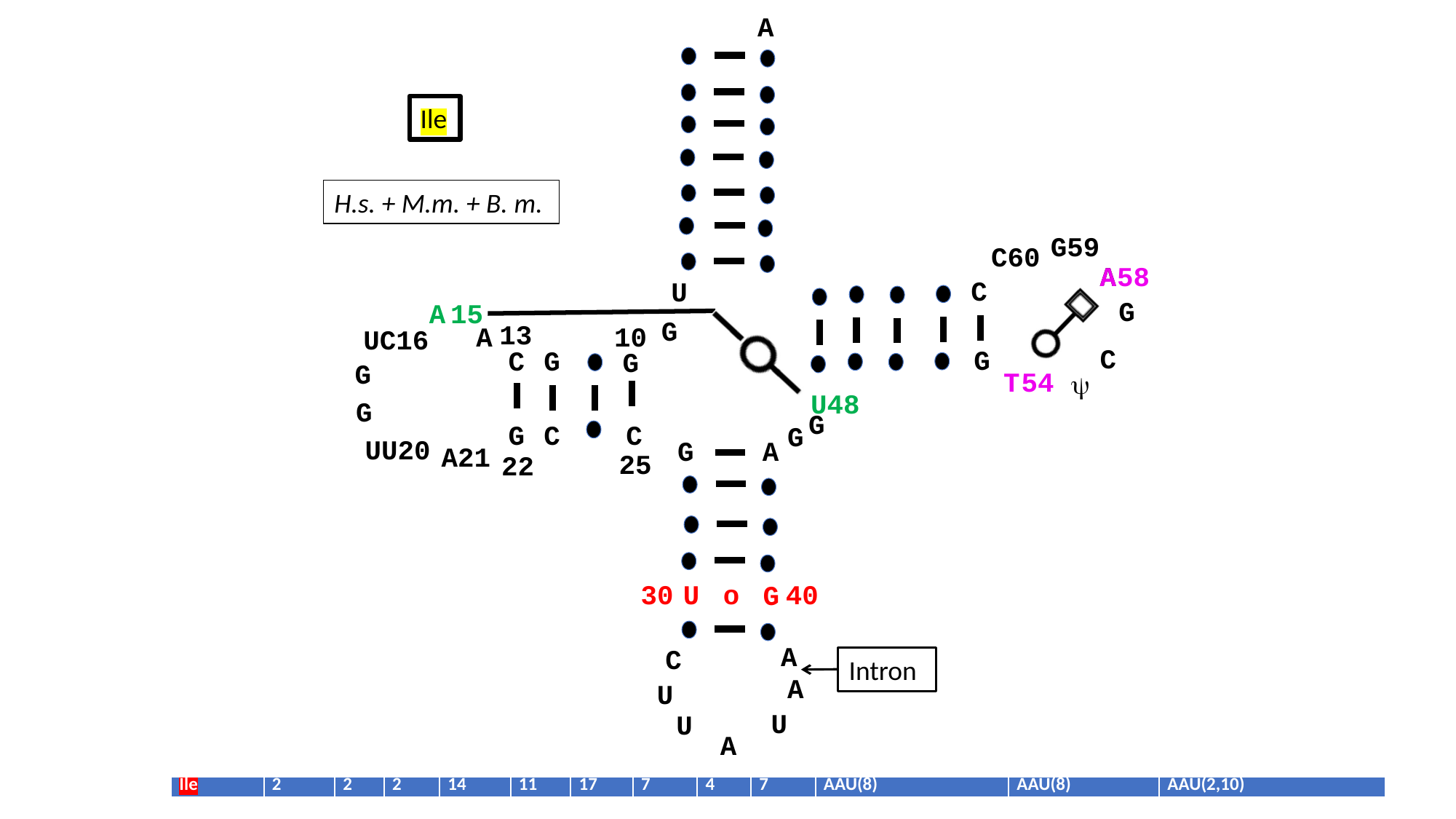

A
Ile
H.s. + M.m. + B. m.
G59
C60
A
58
T
54
A
C
U
G
A
15
U48
G
13
A
10
UC16
©
C
G
C
G
G
y
G
C
G
G
C
G
G
UU20
A
G
A21
25
22
30
o
U
40
G
A
C
Intron
A
U
U
U
A
| Ile | 2 | 2 | 2 | 14 | 11 | 17 | 7 | 4 | 7 | AAU(8) | AAU(8) | AAU(2,10) |
| --- | --- | --- | --- | --- | --- | --- | --- | --- | --- | --- | --- | --- |

## Slide 27
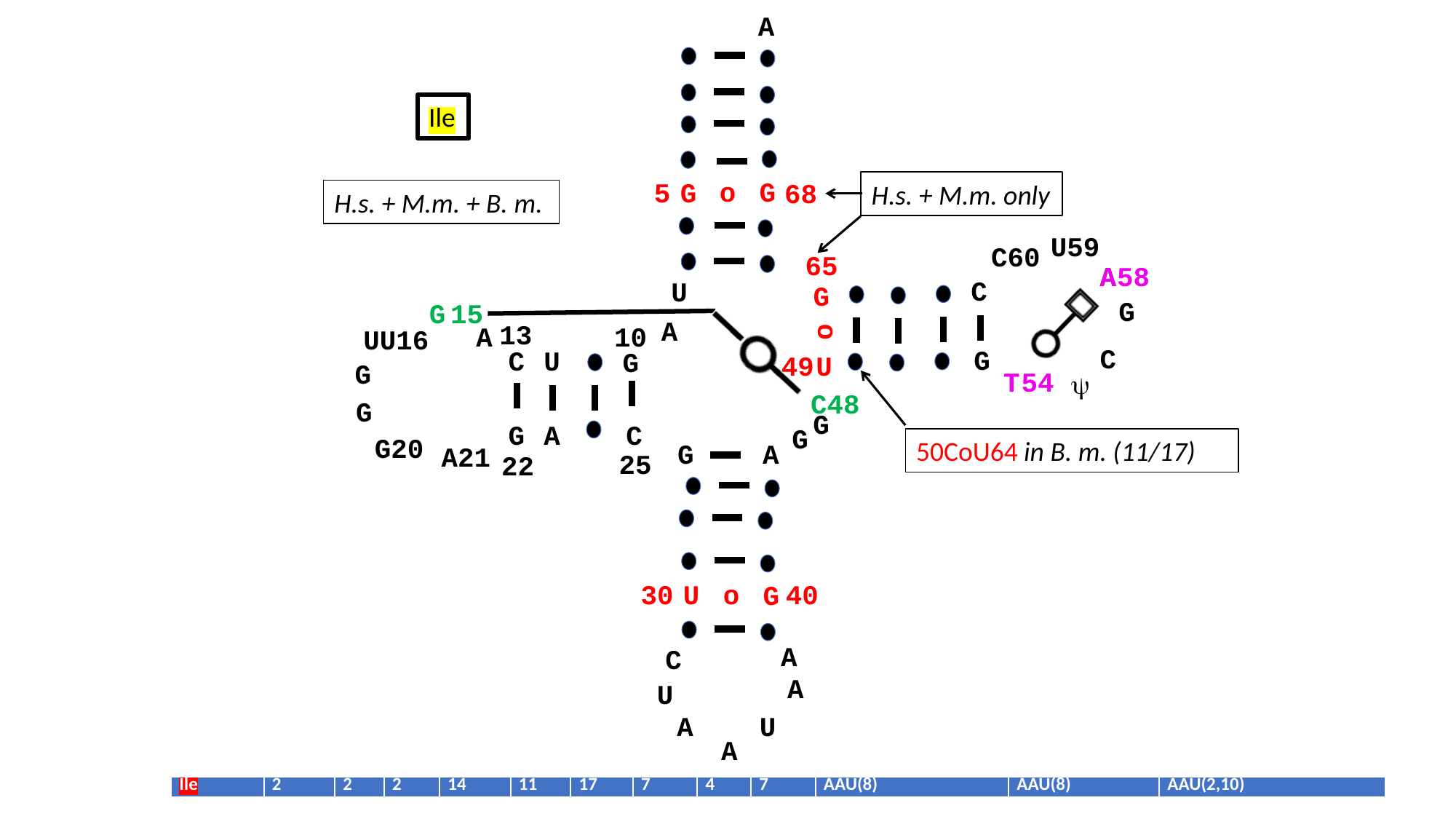

A
Ile
o
G
5
G
68
H.s. + M.m. only
H.s. + M.m. + B. m.
U59
C60
65
A
58
T
54
A
C
U
G
G
G
15
C48
A
13
o
A
10
UU16
©
C
G
C
G
U
49
G
y
U
A
G
G
C
G
G
G20
50CoU64 in B. m. (11/17)
A
G
A21
25
22
30
o
U
40
G
A
C
A
U
A
U
A
| Ile | 2 | 2 | 2 | 14 | 11 | 17 | 7 | 4 | 7 | AAU(8) | AAU(8) | AAU(2,10) |
| --- | --- | --- | --- | --- | --- | --- | --- | --- | --- | --- | --- | --- |

## Slide 28
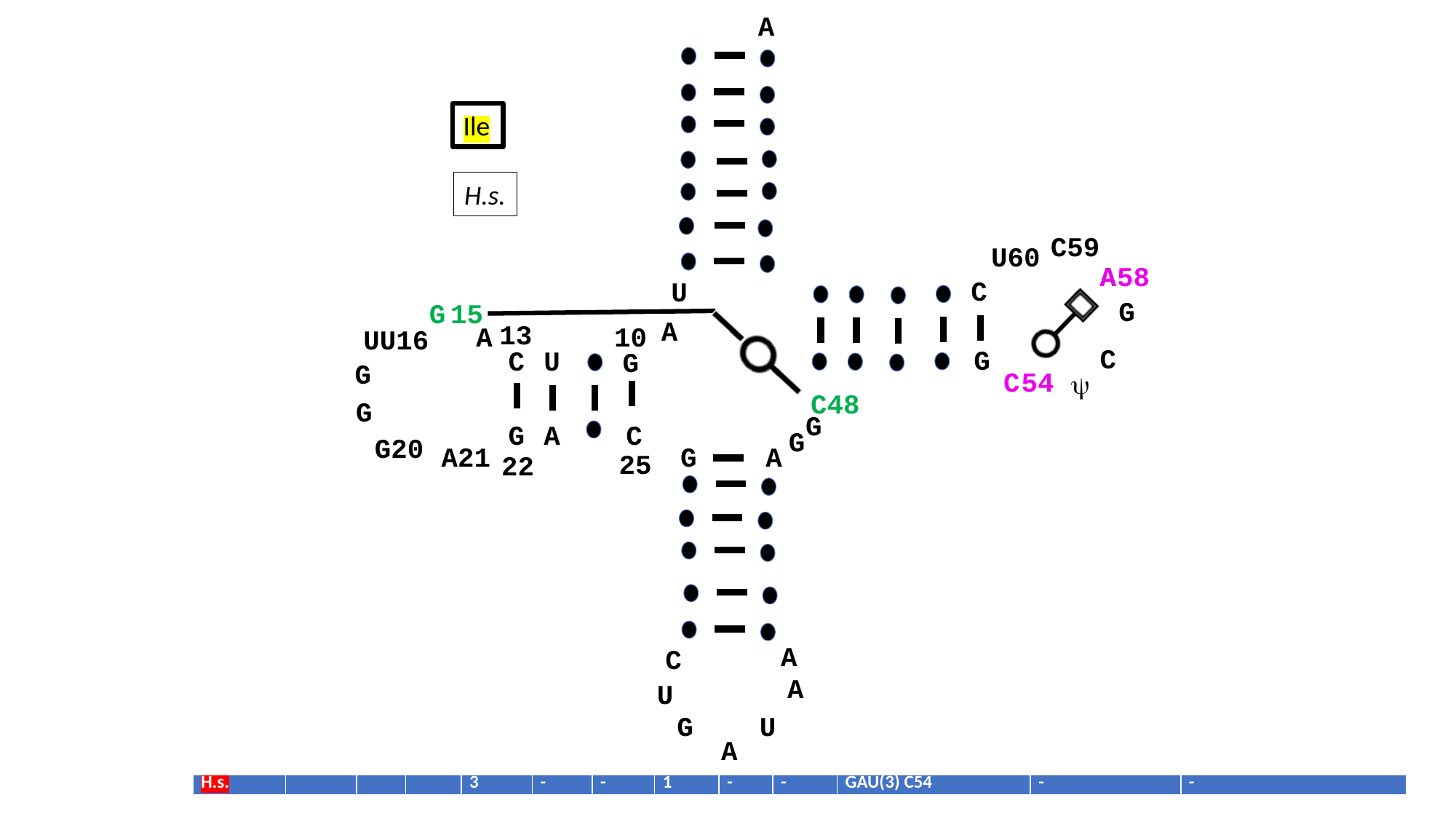

A
Ile
H.s.
C59
U60
A
58
C
54
A
C
U
G
G
15
C48
A
13
A
10
UU16
©
C
G
C
G
G
y
U
A
G
G
C
G
G
G20
A21
A
G
25
22
A
C
A
U
G
U
A
| H.s. | | | | 3 | - | - | 1 | - | - | GAU(3) C54 | - | - |
| --- | --- | --- | --- | --- | --- | --- | --- | --- | --- | --- | --- | --- |

## Slide 29
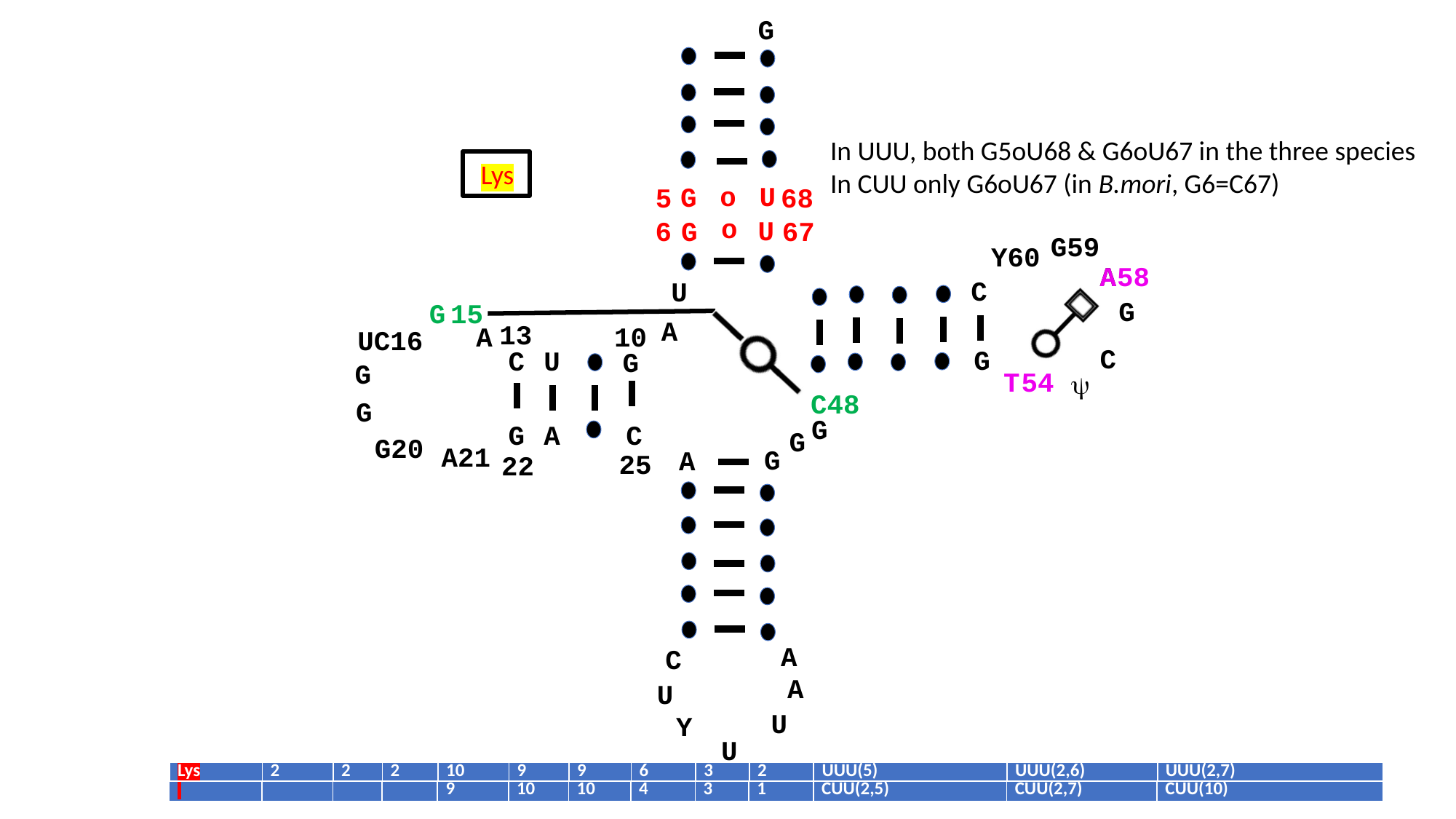

G
In UUU, both G5oU68 & G6oU67 in the three species
In CUU only G6oU67 (in B.mori, G6=C67)
Lys
o
U
G
68
5
o
U
G
6
67
G59
Y60
A
58
T
54
A
C
U
G
G
15
C48
A
13
A
10
UC16
©
C
G
C
G
G
y
U
A
G
G
C
G
G
G20
A21
G
A
25
22
A
C
A
U
U
Y
U
| Lys | 2 | 2 | 2 | 10 | 9 | 9 | 6 | 3 | 2 | UUU(5) | UUU(2,6) | UUU(2,7) |
| --- | --- | --- | --- | --- | --- | --- | --- | --- | --- | --- | --- | --- |
| | | | | 9 | 10 | 10 | 4 | 3 | 1 | CUU(2,5) | CUU(2,7) | CUU(10) |
| --- | --- | --- | --- | --- | --- | --- | --- | --- | --- | --- | --- | --- |

## Slide 30
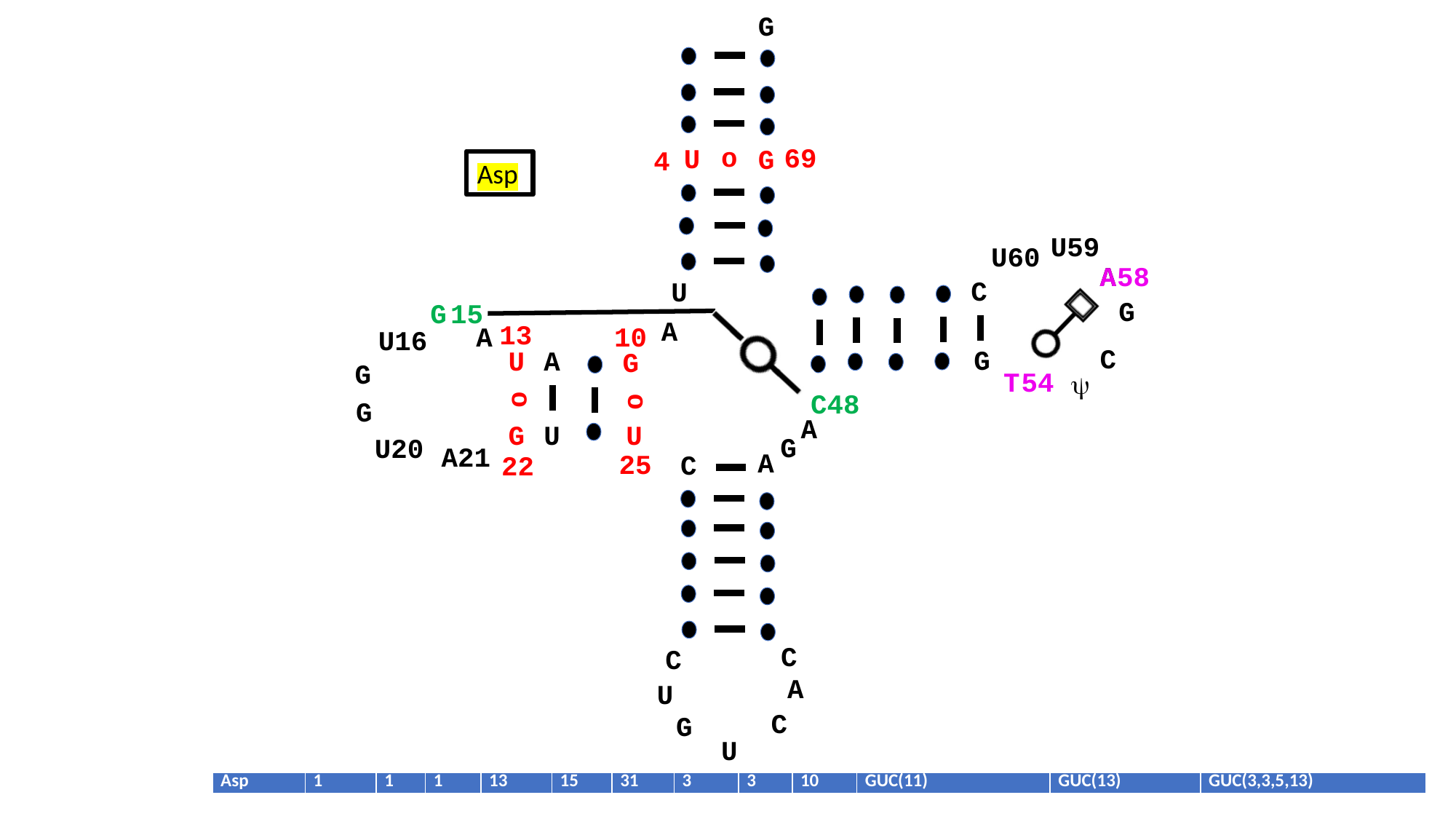

G
o
69
U
G
4
Asp
U59
U60
A
58
T
54
A
C
U
G
G
15
C48
A
A
10
U16
©
C
G
G
G
y
U
G
o
22
13
A
U
o
G
A
U
G
U20
A21
A
25
C
C
C
A
U
C
G
U
| Asp | 1 | 1 | 1 | 13 | 15 | 31 | 3 | 3 | 10 | GUC(11) | GUC(13) | GUC(3,3,5,13) |
| --- | --- | --- | --- | --- | --- | --- | --- | --- | --- | --- | --- | --- |

## Slide 31
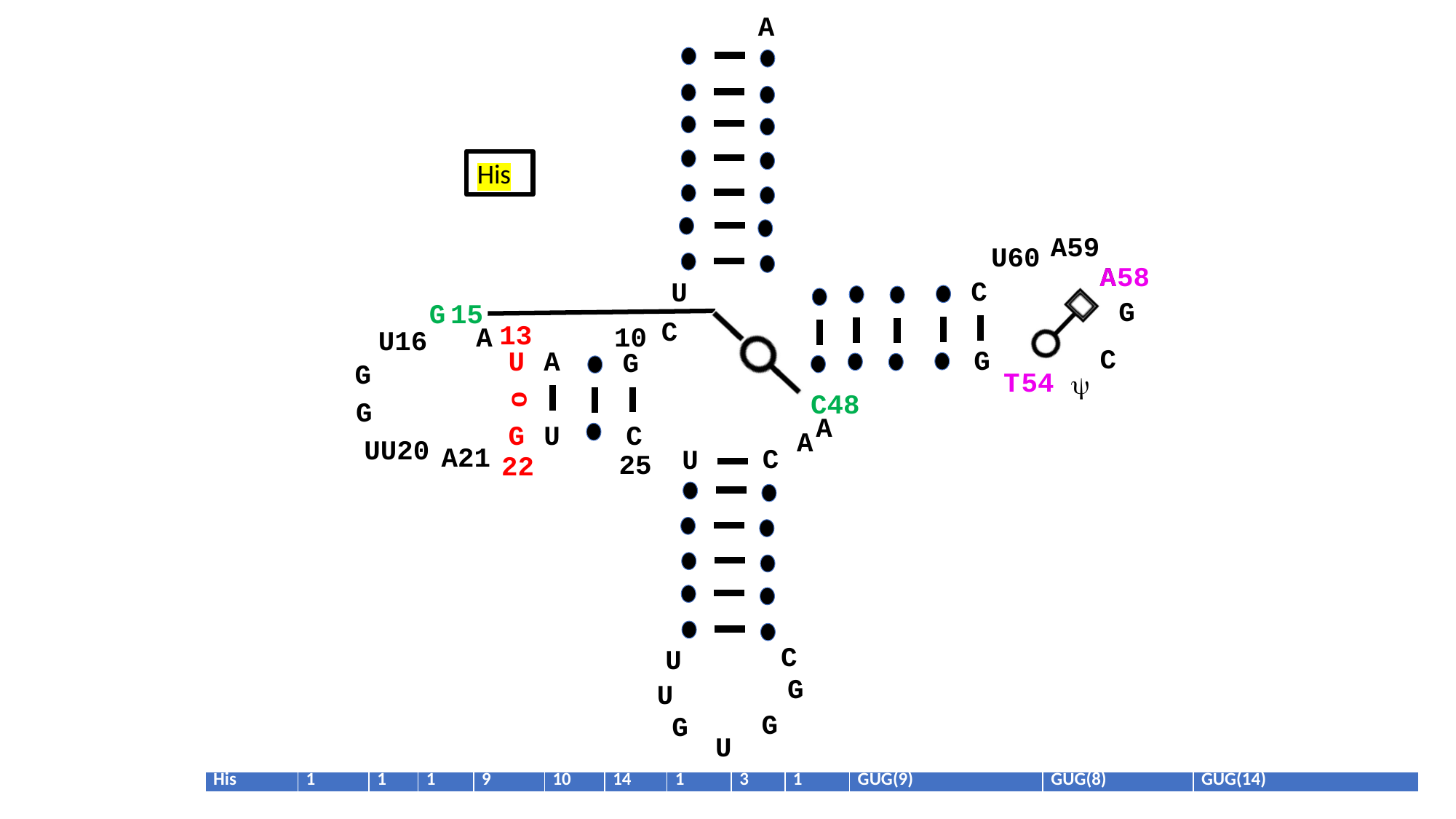

A
His
A59
U60
A
58
T
54
A
C
U
G
G
15
C48
C
A
10
U16
©
C
G
G
G
y
U
G
o
22
13
A
U
G
A
C
A
UU20
A21
C
U
25
C
U
G
U
G
G
U
| His | 1 | 1 | 1 | 9 | 10 | 14 | 1 | 3 | 1 | GUG(9) | GUG(8) | GUG(14) |
| --- | --- | --- | --- | --- | --- | --- | --- | --- | --- | --- | --- | --- |

## Slide 32
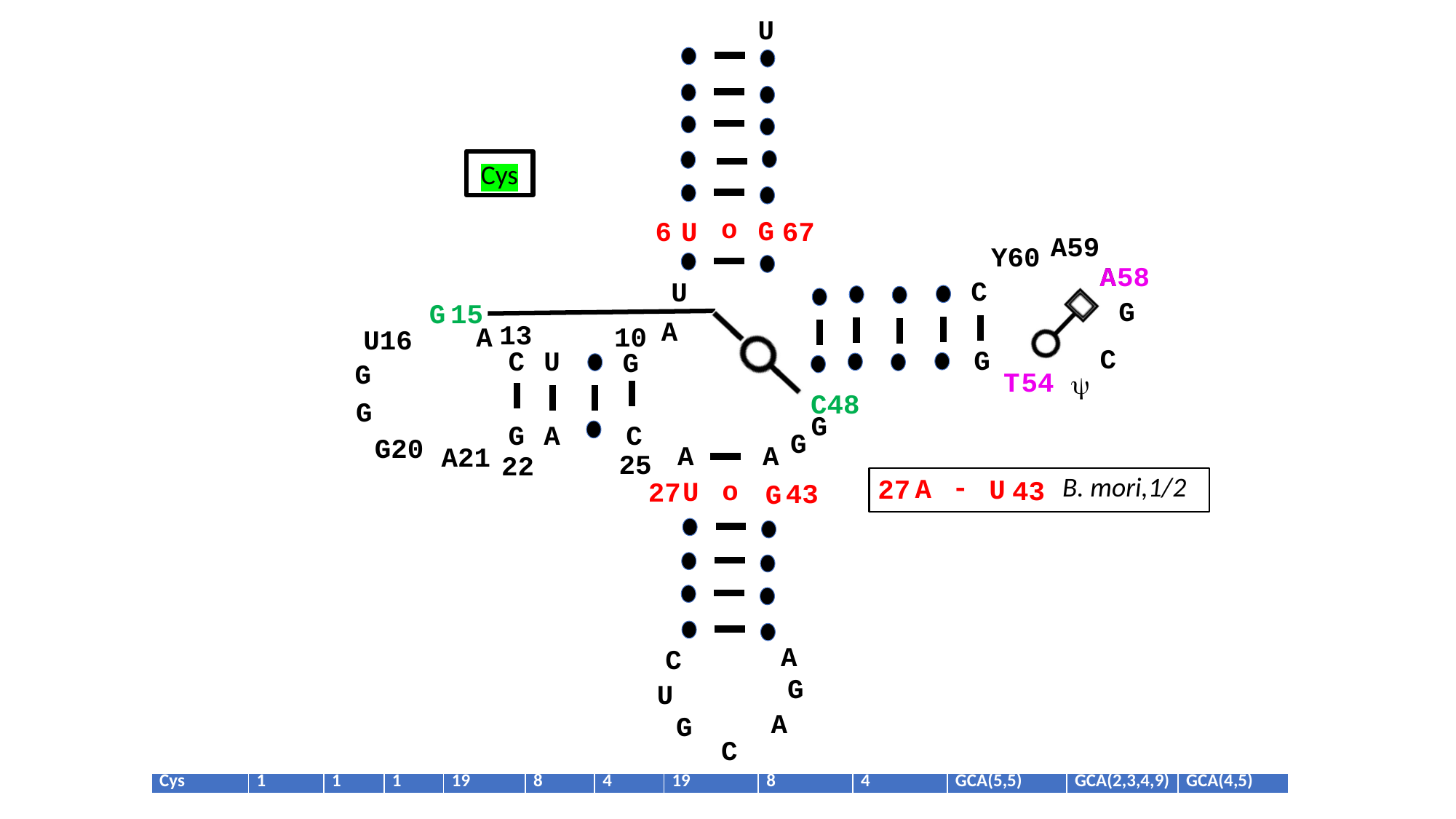

U
Cys
o
G
U
6
67
A59
Y60
A
58
T
54
A
C
U
G
G
15
C48
A
13
A
10
U16
©
C
G
C
G
G
y
U
A
G
G
C
G
G
G20
A
A
A21
25
22
-
A
27
U
43
B. mori,1/2
o
U
27
43
G
A
C
G
U
A
G
C
| Cys | 1 | 1 | 1 | 19 | 8 | 4 | 19 | 8 | 4 | GCA(5,5) | GCA(2,3,4,9) | GCA(4,5) |
| --- | --- | --- | --- | --- | --- | --- | --- | --- | --- | --- | --- | --- |

## Slide 33
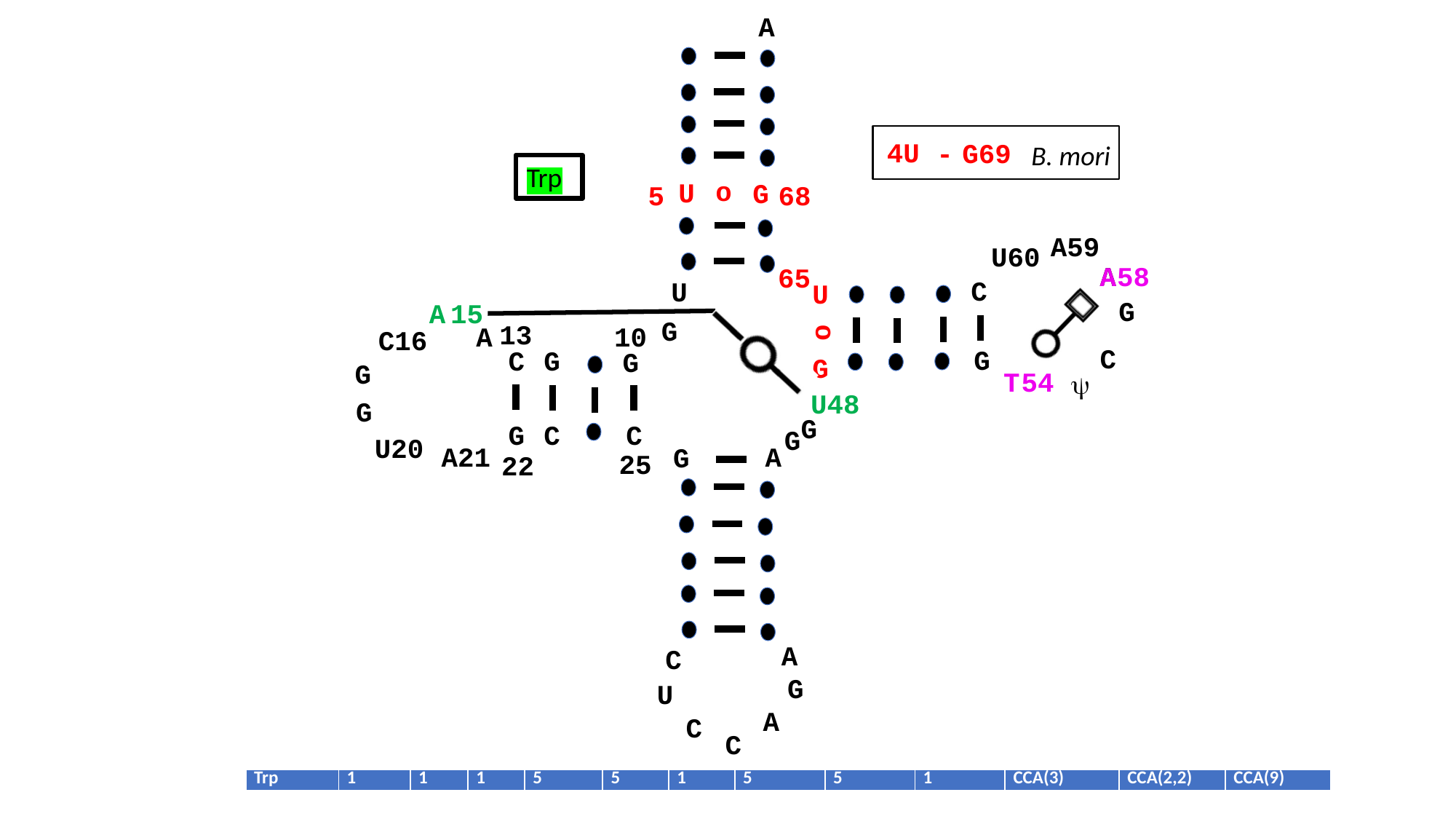

A
4U
G69
-
B. mori
Trp
o
U
G
5
68
A59
U60
A
58
T
54
A
C
U
65
U
G
o
G
A
15
U48
G
A
10
C16
©
C
G
G
G
y
C
G
22
13
G
C
G
G
C
G
U20
A21
A
G
25
A
C
G
U
A
C
C
| Trp | 1 | 1 | 1 | 5 | 5 | 1 | 5 | 5 | 1 | CCA(3) | CCA(2,2) | CCA(9) |
| --- | --- | --- | --- | --- | --- | --- | --- | --- | --- | --- | --- | --- |

## Slide 34
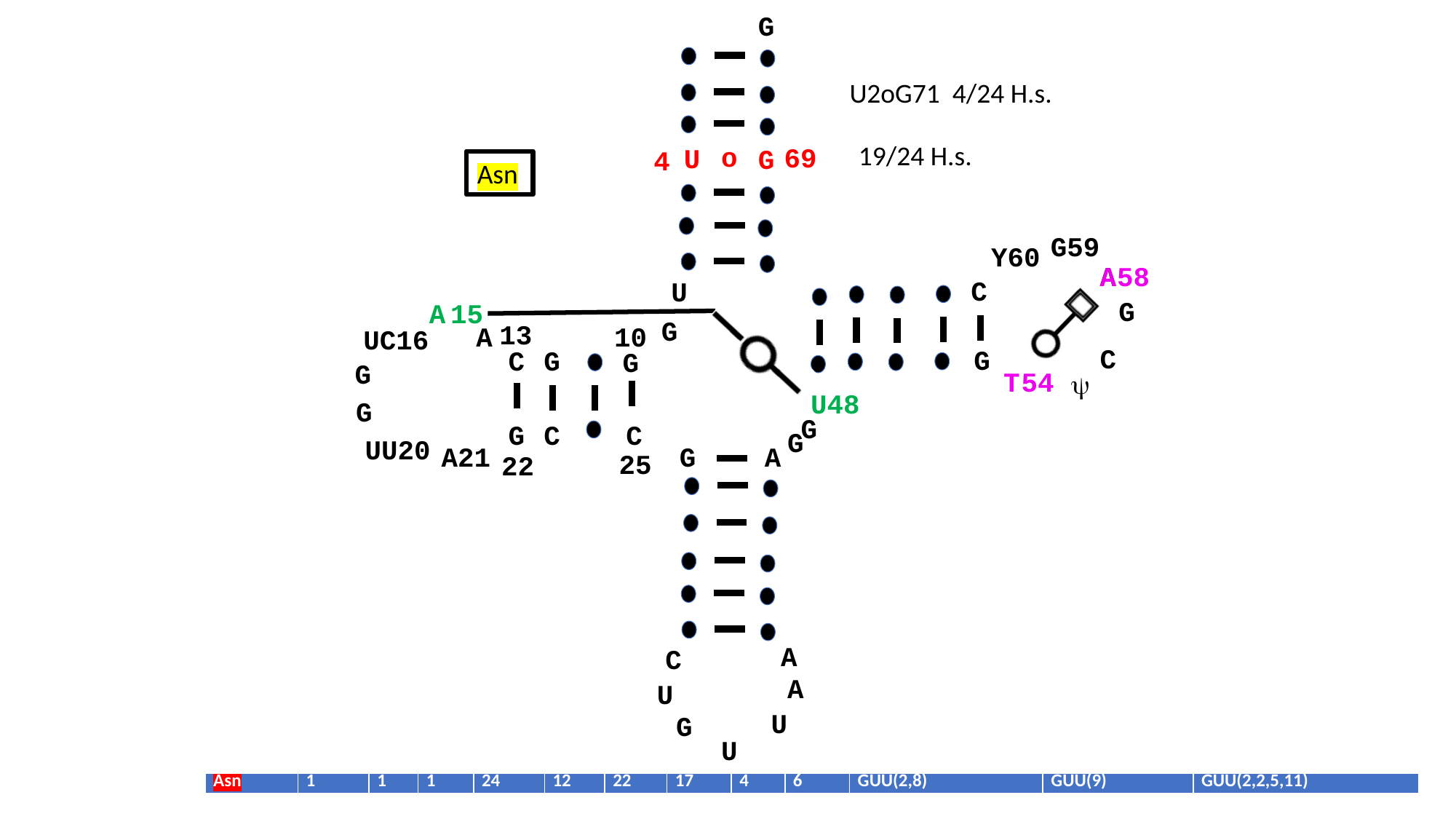

G
U2oG71 4/24 H.s.
19/24 H.s.
o
69
U
G
4
Asn
G59
Y60
A
58
T
54
A
C
U
G
A
15
U48
G
13
A
10
UC16
©
C
G
C
G
G
y
G
C
G
G
C
G
G
UU20
A21
A
G
25
22
A
C
A
U
U
G
U
| Asn | 1 | 1 | 1 | 24 | 12 | 22 | 17 | 4 | 6 | GUU(2,8) | GUU(9) | GUU(2,2,5,11) |
| --- | --- | --- | --- | --- | --- | --- | --- | --- | --- | --- | --- | --- |

## Slide 35
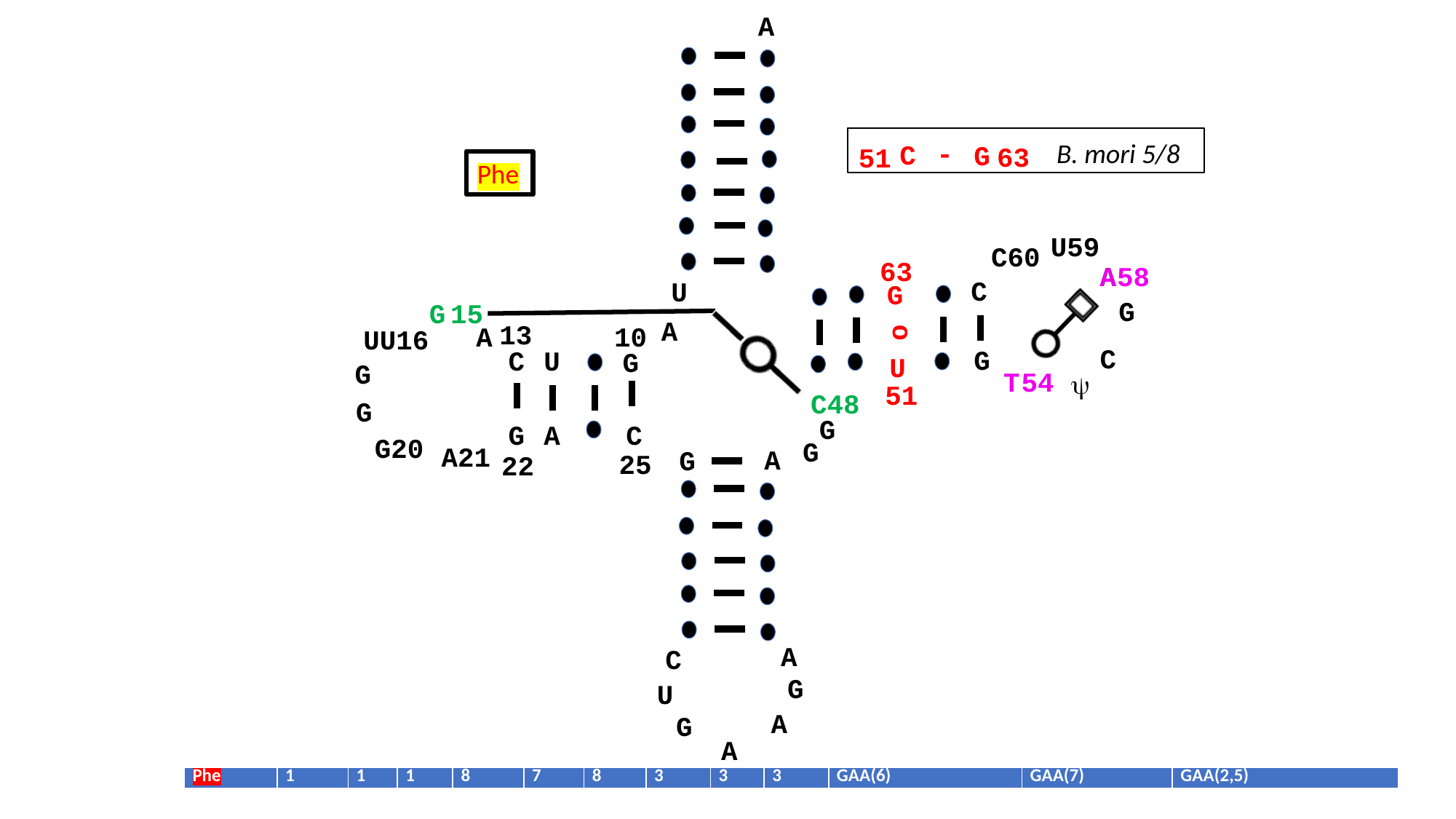

A
B. mori 5/8
-
C
G
63
51
Phe
U59
C60
63
A
58
T
54
A
C
U
G
G
G
15
C48
A
13
A
10
o
UU16
©
C
G
C
G
U
G
y
51
U
A
G
G
C
G
G20
G
A21
A
G
25
22
A
C
G
U
A
G
A
| Phe | 1 | 1 | 1 | 8 | 7 | 8 | 3 | 3 | 3 | GAA(6) | GAA(7) | GAA(2,5) |
| --- | --- | --- | --- | --- | --- | --- | --- | --- | --- | --- | --- | --- |

## Slide 36
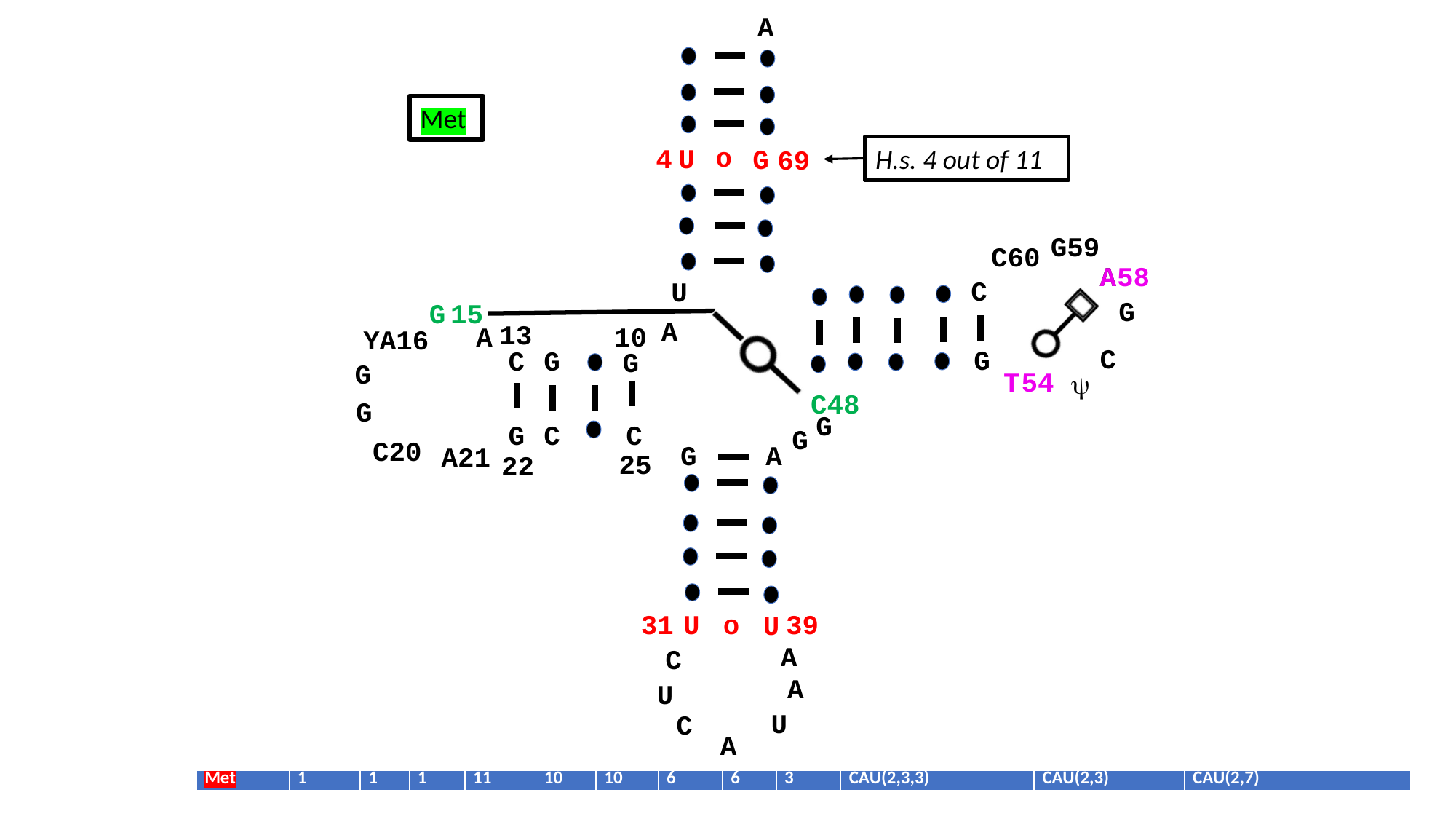

A
Met
o
U
4
G
69
H.s. 4 out of 11
G59
C60
A
58
T
54
A
C
U
G
G
15
C48
A
13
A
10
YA16
©
C
G
C
G
G
y
G
C
G
G
C
G
G
C20
A
G
A21
25
22
31
o
U
39
U
A
C
A
U
U
C
A
| Met | 1 | 1 | 1 | 11 | 10 | 10 | 6 | 6 | 3 | CAU(2,3,3) | CAU(2,3) | CAU(2,7) |
| --- | --- | --- | --- | --- | --- | --- | --- | --- | --- | --- | --- | --- |

## Slide 37
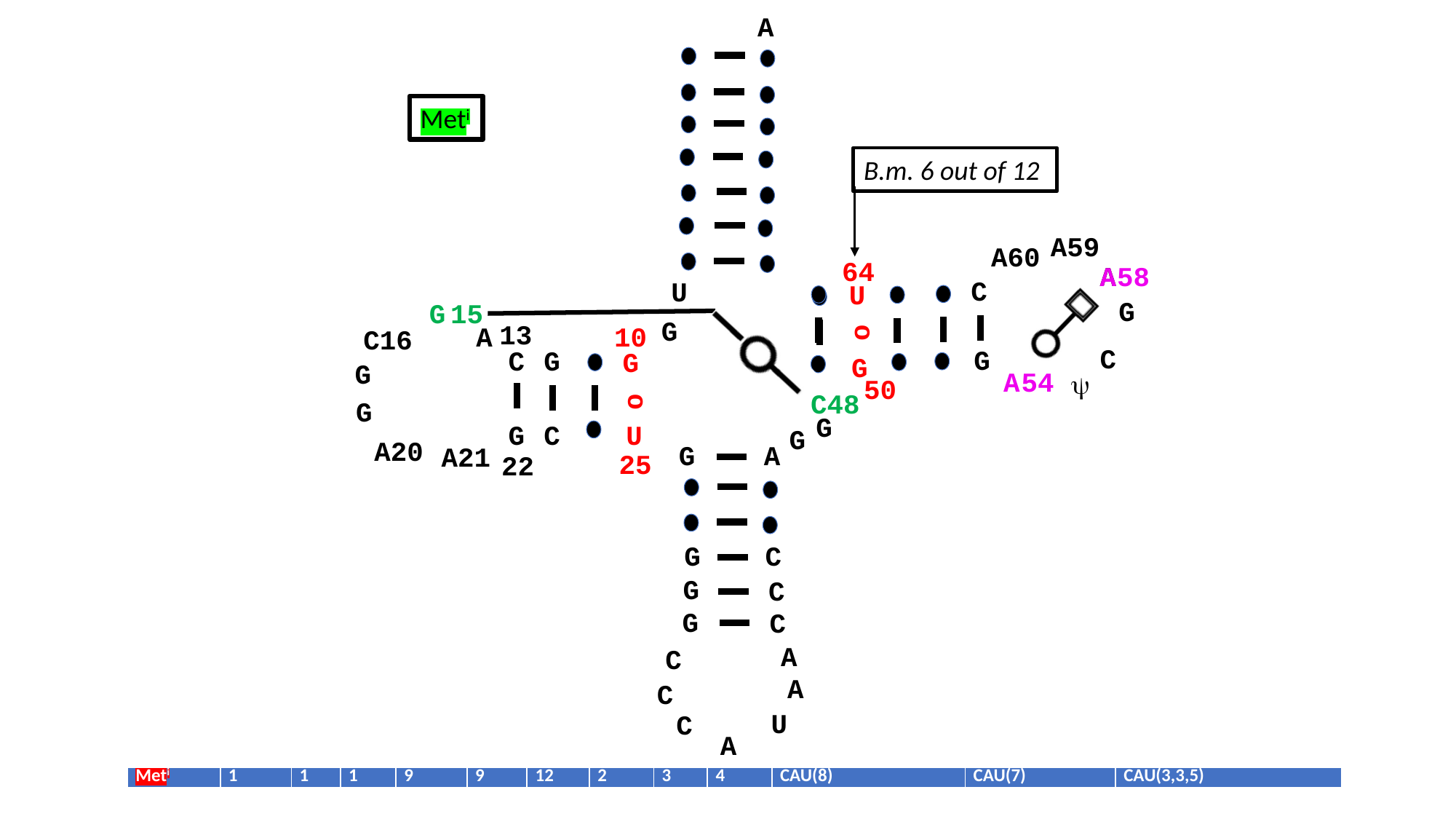

A
Meti
B.m. 6 out of 12
A59
A60
64
A
58
A
54
A
C
U
U
G
G
15
C48
G
13
A
10
o
C16
©
C
G
C
G
G
G
y
50
G
C
o
G
G
U
G
G
A20
A
G
A21
25
22
G
C
G
C
G
C
A
C
A
C
U
C
A
| Meti | 1 | 1 | 1 | 9 | 9 | 12 | 2 | 3 | 4 | CAU(8) | CAU(7) | CAU(3,3,5) |
| --- | --- | --- | --- | --- | --- | --- | --- | --- | --- | --- | --- | --- |

## Slide 38
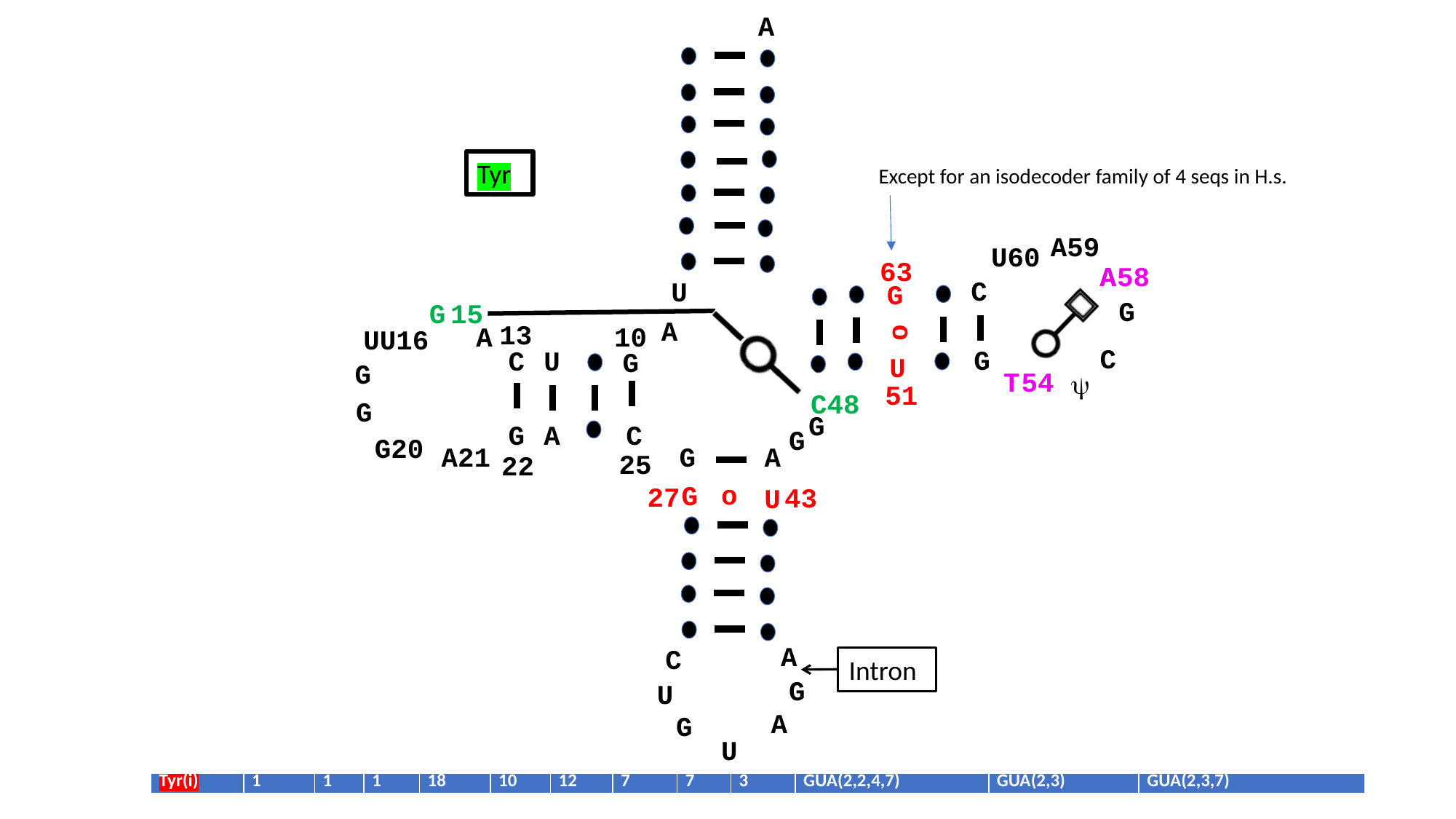

A
Tyr
Except for an isodecoder family of 4 seqs in H.s.
A59
U60
63
A
58
T
54
A
C
U
G
G
G
15
C48
A
13
A
10
o
UU16
©
C
G
C
G
U
G
y
51
U
A
G
G
C
G
G
G20
A21
A
G
25
22
o
G
27
43
U
A
C
Intron
G
U
A
G
U
| Tyr(i) | 1 | 1 | 1 | 18 | 10 | 12 | 7 | 7 | 3 | GUA(2,2,4,7) | GUA(2,3) | GUA(2,3,7) |
| --- | --- | --- | --- | --- | --- | --- | --- | --- | --- | --- | --- | --- |
