# Supplementary material for: Eukaryotic tRNA sequences present conserved and amino acid-specific structural signatures
Source: Nucleic Acids Res. 2022 Apr 5;50(7):4100–12. doi: 10.1093/nar/gkac222 (PMC9023262; doi:10.1093/nar/gkac222)
Supplement: gkac222_Supplemental_Files [file gkac222_supplemental_files.zip › Sup_Data_3 3.zip!/Sup_Data_3/Mammalian/15_48 54_58 Ala AGC 55.pdf]

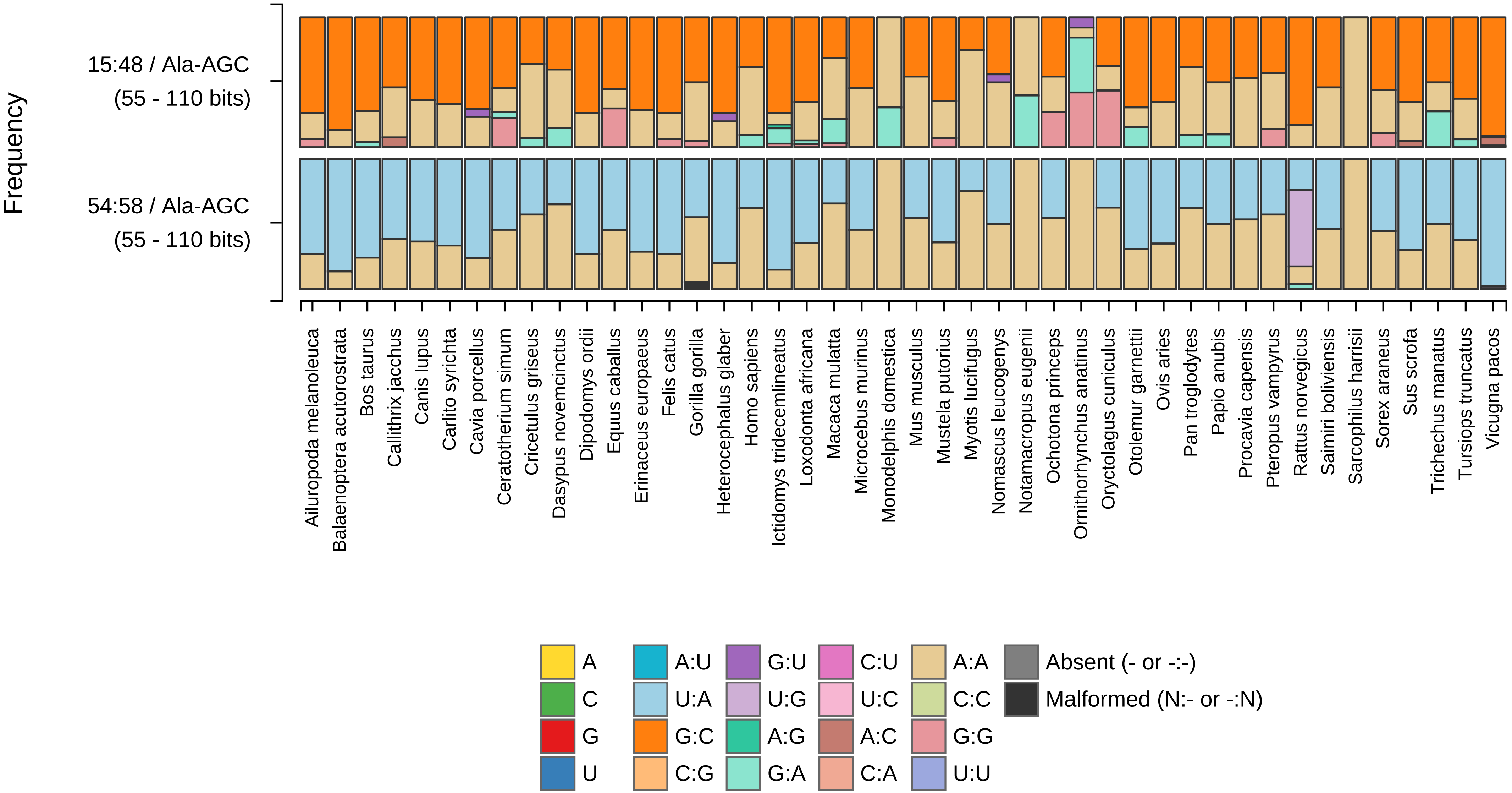
Frequency
15:48 / Ala-AGC
(55 - 110 bits)
54:58 / Ala-AGC
(55 - 110 bits)
Ailuropoda melanoleuca
Balaenoptera acutorostrata
Bos taurus
Callithrix jacchus
Canis lupus
Carlito syrichta
Cavia porcellus
Ceratotherium simum
Cricetulus griseus
Dasypus novemcinctus
Dipodomys ordii
Equus caballus
Erinaceus europaeus
Felis catus
Gorilla gorilla
Heterocephalus glaber
Homo sapiens
Ictidomys tridecemlineatus
Loxodonta africana
Macaca mulatta
Microcebus murinus
Monodelphis domestica
Mus musculus
Mustela putorius
Myotis lucifugus
Nomascus leucogenys
Notamacropus eugenii
Ochotona princeps
Ornithorhynchus anatinus
Oryctolagus cuniculus
Otolemur garnettii
Ovis aries
Pan troglodytes
Papio anubis
Procavia capensis
Pteropus vampyrus
Rattus norvegicus
Saimiri boliviensis
Sarcophilus harrisii
Sorex araneus
Sus scrofa
Trichechus manatus
Tursiops truncatus
Vicugna pacos
A
C
G
U
A:U
U:A
G:C
C:G
G:U
U:G
A:G
G:A
C:U
U:C
A:C
C:A
A:A
C:C
G:G
U:U
Absent (- or -:-)
Malformed (N:- or -:N)
