# Supplementary material for: Eukaryotic tRNA sequences present conserved and amino acid-specific structural signatures
Source: Nucleic Acids Res. 2022 Apr 5;50(7):4100–12. doi: 10.1093/nar/gkac222 (PMC9023262; doi:10.1093/nar/gkac222)
Supplement: gkac222_Supplemental_Files [file gkac222_supplemental_files.zip › Sup_Data_3 3.zip!/Sup_Data_3/Mammalian/5_68_30_40_49_65_Ile GAU.pdf]

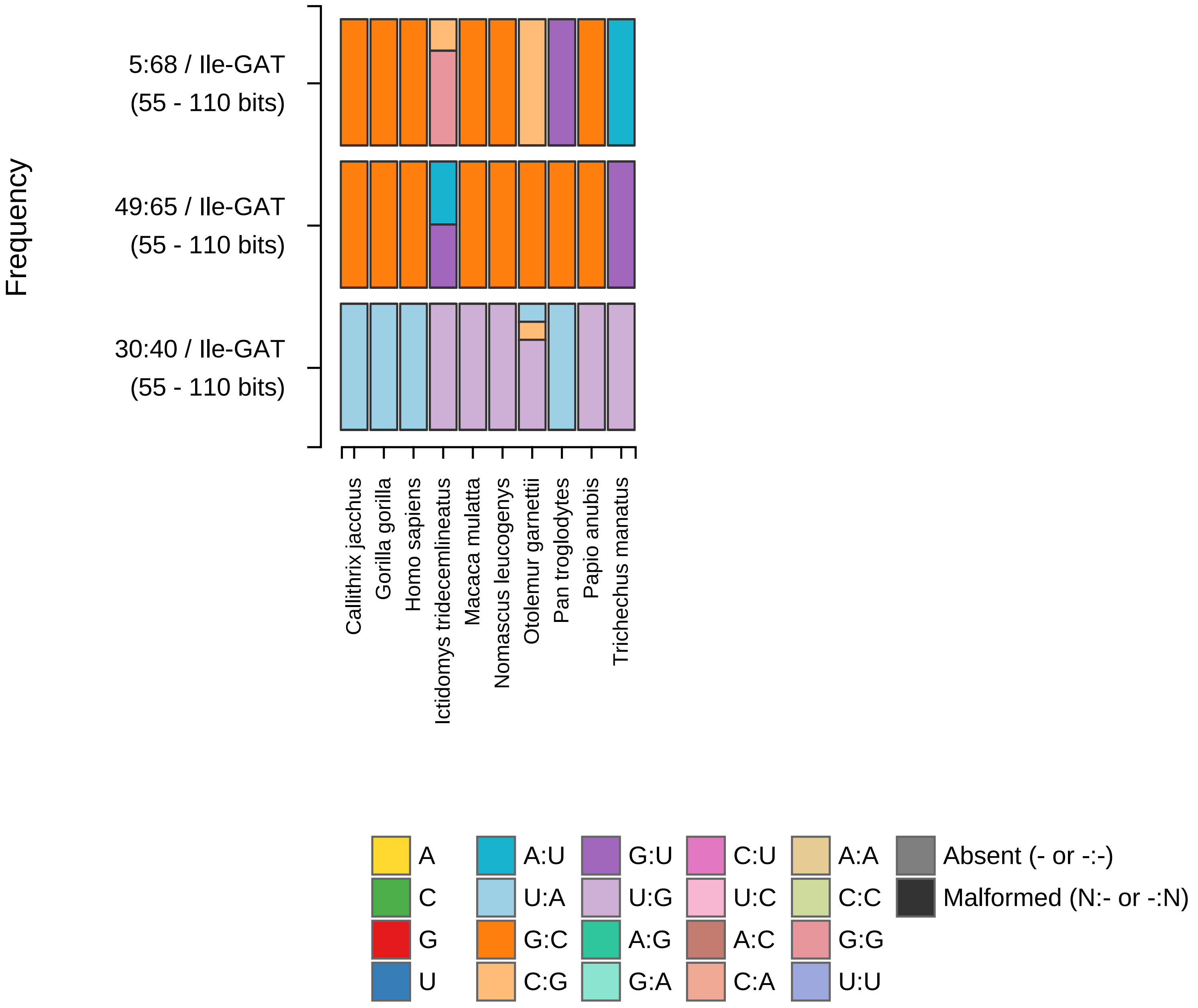
Frequency
5:68 / Ile-GAT
(55 - 110 bits)
49:65 / Ile-GAT
(55 - 110 bits)
30:40 / Ile-GAT
(55 - 110 bits)
Callithrix jacchus
Gorilla gorilla
Homo sapiens
Ictidomys tridecemlineatus
Macaca mulatta
Nomascus leucogenys
Otolemur garnettii
Pan troglodytes
Papio anubis
Trichechus manatus
A
C
G
U
A:U
U:A
G:C
C:G
G:U
U:G
A:G
G:A
C:U
U:C
A:C
C:A
A:A
C:C
G:G
U:U
Absent (- or -:-)
Malformed (N:- or -:N)
