# Supplementary material for: Eukaryotic tRNA sequences present conserved and amino acid-specific structural signatures
Source: Nucleic Acids Res. 2022 Apr 5;50(7):4100–12. doi: 10.1093/nar/gkac222 (PMC9023262; doi:10.1093/nar/gkac222)
Supplement: gkac222_Supplemental_Files [file gkac222_supplemental_files.zip › Sup_Data_3 3.zip!/Sup_Data_3/Insecta/15_48 54_58 30_40 Ala CGC 55.pdf]

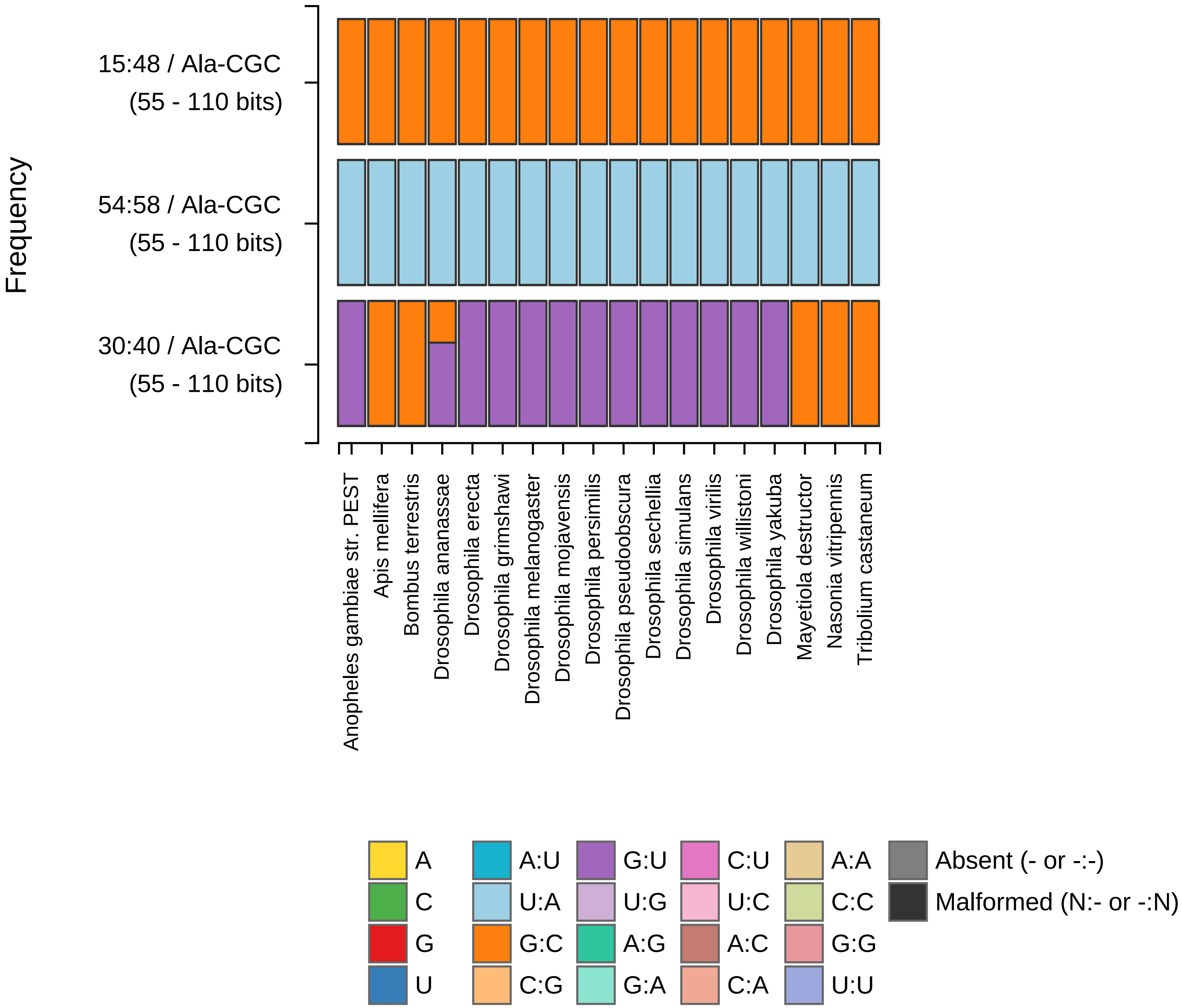
Frequency
15:48 / Ala-CGC
(55 - 110 bits)
54:58 / Ala-CGC
(55 - 110 bits)
30:40 / Ala-CGC
(55 - 110 bits)
Anopheles gambiae str. PEST
Apis mellifera
Bombus terrestris
Drosophila ananassae
Drosophila erecta
Drosophila grimshawi
Drosophila melanogaster
Drosophila mojavensis
Drosophila persimilis
Drosophila pseudoobscura
Drosophila sechellia
Drosophila simulans
Drosophila virilis
Drosophila willistoni
Drosophila yakuba
Mayetiola destructor
Nasonia vitripennis
Tribolium castaneum
A
C
G
U
A:U
U:A
G:C
C:G
G:U
U:G
A:G
G:A
C:U
U:C
A:C
C:A
A:A
C:C
G:G
U:U
Absent (- or -:-)
Malformed (N:- or -:N)
